# The Molecular Processes in the Trabecular Meshwork After Exposure to Corticosteroids and in Corticosteroid-Induced Ocular Hypertension

**DOI:** 10.1167/iovs.61.4.24

**Published:** 2020-04-18

**Authors:** Ilona Liesenborghs, Lars M. T. Eijssen, Martina Kutmon, Theo G. M. F. Gorgels, Chris T. Evelo, Henny J. M. Beckers, Carroll A. B. Webers, Johannes S. A. G. Schouten

**Affiliations:** 1 University Eye Clinic Maastricht, Maastricht University Medical Centre, Maastricht, The Netherlands; 2 Maastricht Centre of Systems Biology (MaCSBio), Maastricht University, Maastricht, The Netherlands; 3 Department of Bioinformatics-BiGCaT, Nutrition and Translational Research in Metabolism (NUTRIM), Maastricht University, Maastricht, The Netherlands; 4 Department of Psychiatry and Neuropsychology, School for Mental Health and Neuroscience, Maastricht University Medical Centre, Maastricht, The Netherlands; 5 Department of Ophthalmology, Canisius Wilhelmina Hospital, Nijmegen, The Netherlands

**Keywords:** corticosteroid-induced ocular hypertension, gene expression, trabecular meshwork, bioinformatics, candidate target genes

## Abstract

**Purpose:**

To identify processes that contribute to corticosteroid-induced ocular hypertension and candidate target genes for treatment.

**Methods:**

A systematic search identified five human microarray datasets investigating the effect of dexamethasone versus a control medium on trabecular meshwork (TM) tissue. After thorough quality control, samples of low quality were removed, and the datasets were integrated. Additionally, a bovine RNA-sequencing dataset allowed to investigate differences in gene expression profiling between cows with and without corticosteroid-induced ocular hypertension (responders vs. nonresponders). The obtained datasets were used as input for parallel pathway analyses. Significantly changed pathways were clustered into functional categories and the results were further investigated. A network visualizing the differences between the responders and nonresponders was created.

**Results:**

Seven functional pathway clusters were found to be significantly changed in TM cells exposed to dexamethasone versus a control medium and in TM cells of responders versus nonresponders: collagen, extracellular matrix, adhesion, WNT-signaling, inflammation, adipogenesis, and glucose metabolism. In addition, cell cycle and senescence were only significantly changed in responders versus nonresponders. The network of the differential gene expression between responders and nonresponders shows many connections between the identified processes via shared genes.

**Conclusions:**

Nine functional pathway clusters synthesize the molecular response to dexamethasone exposure in TM cells and are likely to be involved in the pathogenesis of corticosteroid-induced ocular hypertension.

Corticosteroids are widely used within the field of ophthalmology. However, they induce ocular hypertension (OHT), also known as a corticosteroid response, in approximately 18% to 36% of patients. This percentage can be as high as 92% in patients with primary open angle glaucoma (POAG).^1^ A sustained increase in intraocular pressure (IOP) may cause damage to the optic nerve, leading to visual field loss and eventually blindness. Corticosteroid-induced OHT is likely caused by molecular changes in the trabecular meshwork (TM) that increases the outflow resistance; however, the pathogenesis is not yet fully understood.^2^^–^^5^ The current treatment attempts to lower the IOP by using traditional antiglaucoma drugs or laser treatment. However, these do not target the pathogenic mechanisms of a corticosteroid response. Therefore corticosteroids often have to be reduced or even ceased to lower the IOP, which impedes the use of these clinically valuable drugs.

Multiple omics studies investigated the differential gene expression profiles in the TM after exposure to corticosteroids.^6^^–^^13^ These individual studies revealed that genes involved in processes, such as cell adhesion, cell cycle, extracellular matrix, inflammation, and immune response, might be involved in the pathogenesis of corticosteroid-induced OHT. However, these studies comprised relatively small sample sizes, used various study methods with different cell types, and a diverse duration of exposure and dosage to the used corticosteroid. In addition, most studies did not differentiate between patients with and without a corticosteroid response. It is therefore not known whether the observed processes directly relate to corticosteroid-induced OHT, or only reflect the effects of corticosteroids on the TM.

Within this study, we integrated the publicly available gene expression data investigating the effect of dexamethasone on the TM. In addition, Bermudez et al.^14^ kindly provided a bovine RNA-sequencing dataset in which a distinction between eyes with and without a corticosteroid response was made. Bioinformatics analyses on these datasets identified which processes are significantly changed in the TM after exposure to corticosteroids, and in the TM of bovine responders. The obtained results were visualized and compared, which allowed the identification of specific molecular processes that are likely to be involved in corticosteroid-induced OHT. These processes can be further explored for targeted drug therapies, which specifically influence the molecular pathogenesis of corticosteroid-induced OHT.

## Methods

### Systematic Search

A systematic search was conducted in Gene Expression Omnibus (GEO; http://www.ncbi.nlm.nih.gov/geo/)^15^^,^^16^ and ArrayExpress (http://www.ebi.ac.uk/arrayexpress)^17^ to identify publicly available data of genome-wide expression studies in which the effects of corticosteroids on the gene expression profiles of TM cells had been investigated. The search term used was: “trabecular meshwork AND corticosteroids”. To not miss any eligible studies, the search was repeated with “trabecular meshwork” as the only search term. The search was not restricted on publication status or date of publication and was last updated on June 4, 2019.

Five human microarray studies investigating the differences in gene expression profiles between TM cells exposed to dexamethasone and a control medium were identified. None of these studies specified whether the included individuals were responders or nonresponders.

However, the RNA-sequencing study of Bermudez et al.^14^ investigated the differences in gene expression profiles of TM cells of identified responders and nonresponders. This study allowed us to investigate the differences between responders and nonresponders. In addition, the general effect of dexamethasone on the TM cells, analogous to the human microarray data, could also be investigated with the data from this study.

### Preprocessing and Quality Control of the Human Data

The identified datasets were submitted to a quality control and preprocessing workflow. If necessary, the authors of the datasets were approached to obtain additional information. When available, both the raw and normalized data for each identified human microarray study was downloaded from GEO or ArrayExpress. Based on the availability of the provided data, we used the normalized data of the researchers or performed quantile normalization on the raw data ourselves. Quality control was performed on both the raw and normalized data as described previously.^18^ The quality assessment of the samples and studies was independently performed by two researchers (L.M.T.E and I.L.). Thereafter, the results were compared, and in case of disagreement consensus was achieved by discussion. The results of the performed quality control for each dataset were visualized in different plots. These plots were assessed for homogeneity of the data, the signal strength of the different samples in the study, the correlation of expression, and the way in which the samples cluster (control vs. exposure). Following, in case samples appeared divergent based on combined interpretation of the plots of the quality control or in case a study showed an overall low quality, they were excluded for further analysis. After data preprocessing, statistical analysis to compare TM cells treated with and without dexamethasone was performed using the *limma* package for R (linear regression models) as available from Bioconductor (http://www.bioconductor.org).^19^^,^^20^ The obtained results per dataset comprised the measured genes and their mean expression, log_2_ fold change (LogFC), T-statistic and *P* value of the adapted *t*-test. Because our main goal was to combine all studies, we did not remove genes with a low expression from the datasets to obtain a dataset that was as complete as possible. As dexamethasone was investigated in all included studies, and we wanted to compare studies with a similar study design, only the samples exposed to dexamethasone were used for further analysis.

Systematic application of the earlier-mentioned steps led to high-quality data on differential gene expression for each of the five included studies, which were used for further integrated analyses.

#### Integration of Data

To obtain results that are less dependent on study differences of individual studies, we combined the high-quality and preprocessed gene expression datasets of the included studies. To make the data annotation uniform, we converted the used probe-identifiers within each dataset into Ensembl gene identifiers. If an individual was replicated multiple times within one study, the average value across these samples was computed for each gene to give each individual the same weight in the analysis. If a gene was tested multiple times within one dataset, we used the gene with the highest absolute value for LogFC*-log_10_ (*P* value), as this represents the highest change based on the LogFC and *P* value. This was used in later analysis as well. Therefore only one value was assigned per tested gene and per individual. After preparing the individual sets, we merged them based on matching Ensembl gene identifiers. Then a joint estimate of each gene's LogFC and *P* value was calculated by computing their weighted averages over all datasets. To take differences in study size into account, weights were assigned to each separate dataset based on the total number of individuals included within that dataset. The average weighted LogFC and *P* value were calculated as shown in Formulas 1 and 2.

To ensure robust estimates, only genes that had been tested in at least four out of five studies were kept in the final combined dataset. Also, by only using the genes that had been tested in multiple studies, the potential dominance of larger studies that tested more genes were avoided. The obtained integrated human gene expression dataset, containing an average weighted LogFC and *P* value for each gene, was used as input for pathway analysis.

$$\;\mathrm{Average}\;\mathrm{weighted}\;\mathrm{LogFC}\;=\frac{\sum_{i=1}^{n}\left(logFC_{i}*-\log_{10}p_{i}\right)}{\sum_{i=1}^{n}\left(\text{-}\log_{10}p_{i}\right)}$$

Formula 1. Calculation of the average weighted LogFC, *n* is the number of studies for which the gene was measured.

$$\mathrm{Average}\;\mathrm{weighted}\;P\;-\mathrm{value}\;=\frac{\sum_{i=1}^{n}\left(\text{-}\log_{10}p_{i}\right)}{n}$$

Formula 2. Calculation of the average weighted *P* value, *n* is the number of studies for which the gene was measured.

### Preprocessing of the Bovine Data

Bermudez et al.^14^ kindly provided the complete statistical results of their RNA-sequencing experiment: the averaged Fragments Per Kilobase Million (FPKM) for each tested gene after exposure to dexamethasone and a control medium in both responders and nonresponders.

We processed the data to make the following comparisons: (1) the differences in gene expression between responders and nonresponders, and (2) the differences in gene expression after exposing the TM to dexamethasone and ethanol (control medium).

To make the first comparison, we calculated the LogFC of the responders (LogFC_R_) and nonresponder (LogFC_NR_) as shown in Formula 3. The plus one was added to avoid minus infinity-values. Thereafter, we deducted the LogFC of the responders with the LogFC of the nonresponders (LogFC_R_ – LogFC_NR_).

$$LogFC_{R}=\;log_{2}\left(A+1/B+1\right)$$

$$LogFC_{NR}=\;log_{2}\left(C+1/D+1\right)$$

Formula 3. A = FPKM of responders treated with dexamethasone; B = FPKM of responders treated with ethanol; C = FPKM of nonresponders treated with dexamethasone; D = FPKM of nonresponders treated with ethanol.

To make the second comparison, the mean change in LogFC between TM cells treated with and without dexamethasone was obtained by calculating the average of the LogFC_R_ and the LogFC_NR_ for each gene. As no individual sample measurements were available, we could not calculate a significance value.

### Pathway Overrepresentation Analysis

After performing the earlier-mentioned steps, we performed pathway analysis on the integrated human dataset and the two datasets generated from the bovine RNA-sequencing experiment. A pathway overrepresentation analysis allows the identification of the molecular pathways in which the differentially expressed genes are significantly more present than expected by chance based on the entire dataset. To do so, criteria for a gene to be differentially expressed needs to be defined first. In our case, a gene with an absolute LogFC >0.58 (representing at least a 50% change on original scale of absolute numbers) and a *P* value < 0.05 was defined as differentially expressed for the integrated human dataset. For the bovine set, only the LogFC cutoff was used because no *P* values were available. In the results of the pathway analysis, pathways with a Z-score ≥1.96, a permuted *P* value < 0.05, and >3 changed genes in the pathway were considered significantly changed.

The pathway overrepresentation analysis was performed in PathVisio, which is an open access tool.^21^^,^^22^ To connect measured genes to the corresponding pathway elements, a human identifier mapping database is needed, which was downloaded from www.pathvisio.org (version: Hs_Derby_Ensembl_91.bridge). For the bovine datasets, the gene identifiers provided by the authors were already converted to human HGNC (HUGO Gene Nomenclature Committee) symbols. Therefore we also used the human identifier mapping database and the human pathways to perform pathway analysis on this dataset. As previously described,^18^ we used the pathways of three widely adopted pathway databases WikiPathways,^23^^,^^24^ KEGG,^25^^–^^27^ and Reactome.^28^^,^^29^ Their content was downloaded on December 6, 2018, and combined into one collection to obtain larger pathway coverage. The overrepresentation scores (Z-scores) were calculated in one run for all included pathways of all three databases.

### Clustering of the Pathway Results

The significantly changed pathways of each dataset were clustered into functional categories. This was based on the molecular mechanisms that are captured in the pathways and was performed by human curation after careful investigation of the results.

### Network Analysis

Cytoscape (www.cytoscape.org), an open access tool, was used to perform network analysis in which the genes of the identified functional categories were combined into one network of connected genes.^30^ We created a network for the bovine data comparing responders and nonresponders. To improve visualization, we only showed the differentially expressed genes (absolute LogFC >0.58).

**Figure 1.**
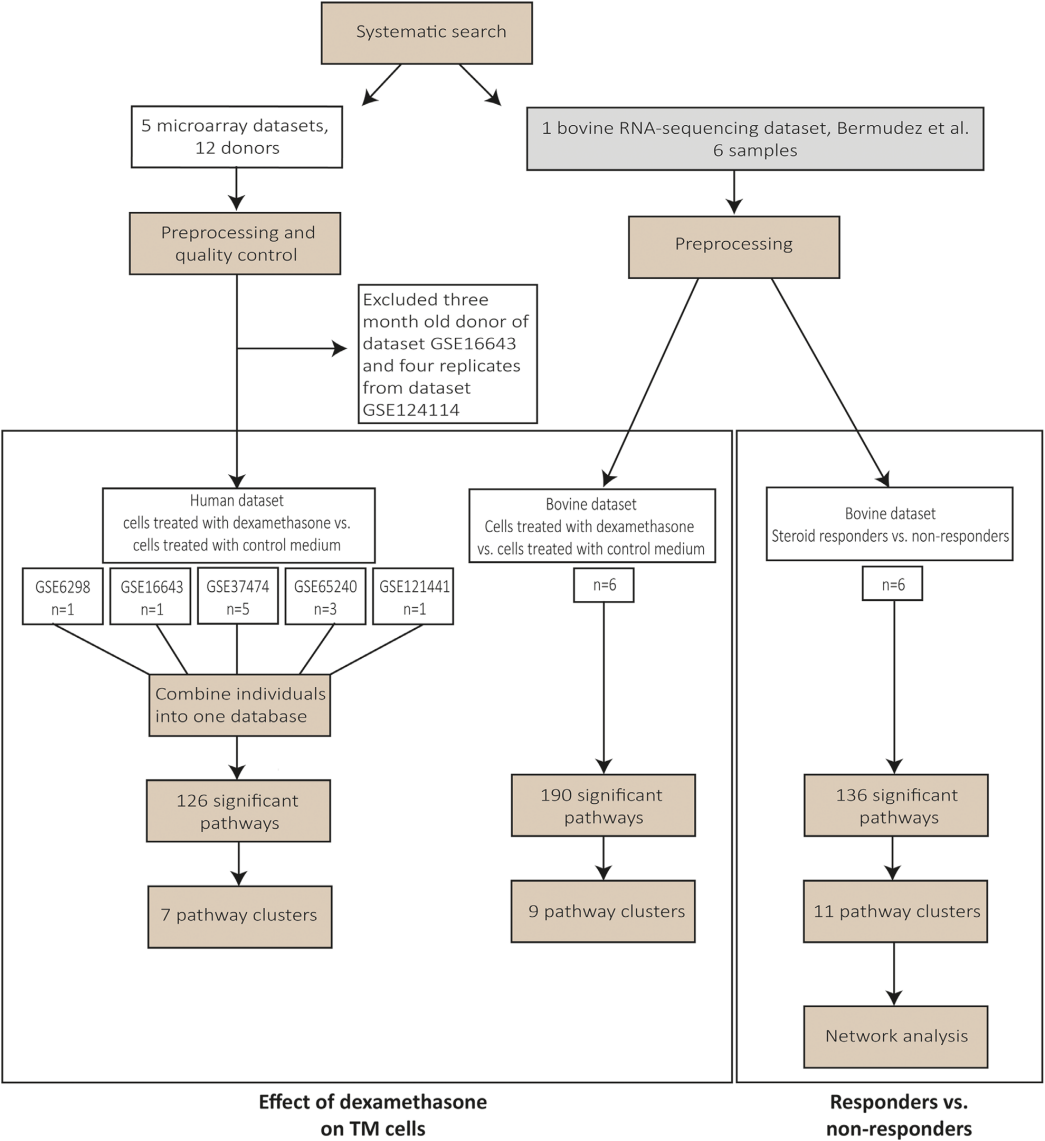
Flowchart of the methods and results.

A flowchart overviewing the methods and results is shown in Figure 1.

(Note: The study was performed in accordance with the tenets of the Declaration of Helsinki.)

## Results

### Systematic Search

The systematic search in GEO identified five studies investigating the effect of corticosteroids on the TM, which were all selected for further analysis. A search with the broader search term “trabecular meshwork” showed 30 datasets, however, none of them were additionally relevant. Both searches were also performed in ArrayExpress revealing no other studies.

### Included Datasets

After the systematic search, the datasets of five different human microarray studies were selected for further analyses: GSE6298 (Fan et al.^6^), GSE16643 (Nehmé et al.^10^), GSE37474 (Kwon et al.,^8^ concept article received after contacting the authors), GSE65240 (Matsuda et al.^9^), and GSE124114 (Faralli et al.^31^) In addition, the statistical results of the bovine RNA-sequencing experiment by Bermudez et al.^14^ were used in the analysis.

The key characteristics of the included studies are listed in Table 1. The most important features are described here. All studies used cells derived from TM tissue, which was exposed to dexamethasone and a control medium. Hereafter, total RNA was extracted and used for microarray analysis, except for the study of Bermudez et al.^14^ in which RNA-sequencing was performed. Most of the included studies used cultured TM cells, however, dataset GSE37474 and Bermudez et al.^14^ used a perfusion organ culture system.

**Table 1.** Characteristics of the Included Studies

	Fan et al.^6^ GSE6298 (2008)	Nehmé et al.^10^ GSE16643 (2009)	Kwon et al.^8^ GSE37474 (2012)	Matsuda et al.^9^ GSE65240 (2015)	Faralli et al.^31^ GSE124114 (2019)	Bermudez et al.^14^ Bovine RNA-sequencing (2017)
**Study design**	Human cultured TM cells	Human cultured TM cells	Perfusion organ culture system with human donor eyes. Dissection of TM cells after exposure	Human cultured TM cells	Two human TM cell cultures from the same donor	Perfusion organ culture system with bovine donor eyes to define responders and nonresponders. Contralateral eye was used for cell isolation and culture
**Use of postmortem tissue**	Yes	Yes	Yes	Yes	Yes	Yes
**Passage cells**	Eighth	Third–sixth	Not specified	Not specified	Second	>3
**Control medium**	0.0025% and 0.025% BA	0.1% (v/v) DMSO	2 µL/min DMEM	DMEM with 10% fetal calf serum	0.1% ethanol	0.1% ethanol
**Number of included donors**	1 donor	2 donors	5 donors (pair eyes)	3 donors	1 donor	6 donors (pair eyes)
**Replicates**	3 replicates	3 replicates	No replicates	No replicates	18 replicates	No replicates
**Age**	52 years	3 months and 35 years	72 year (mean)	Not specified	27 years	Adult cows
**History of glaucoma**	No	Not specified	No	Not specified	Not specified	Not specified
**Steroid response**	Not specified	Not specified	Unclear	Not specified	Not specified	Yes
**Dexamethasone**	100 nM Medium changed every other day	1 µM = 1000 nM Medium not changed	100 nM Prefusion system	100 nM Medium changed every 2 days	500 nM Medium change not defined	100 nM Medium changed every other day
**Duration of exposure (days)**	7	1 (24 hours)	10	14	6	7
**Micro** **array/RNA-seq** **uenc** **ing**	Stanford Human cDNA SHEW	Agilent-014850 Whole Human Genome Microarray 4 × 44K G4112F	Affymetrix Human Genome U133 Plus 2.0 Array	Agilent-028004 SurePrint G3 Human GE 8 × 60K Microarray Agilent-039494 SurePrint G3 Human GE v2 8 × 60K Microarray 039381	Affymetrix Human Gene 1.0 ST Array	Illumina Human Methylation 450K Chips

BA, benzyl alcohol; DMEM, Dulbecco's modified Eagle's medium; DMSO, dimethyl sulfoxide.

### Quality Control of the Human Data

The complete reports of the quality control of the five human microarray datasets are available in Supplementary File S1. The quality control showed that all datasets were of good quality. At sample level, one donor of dataset GSE16643 and four replicates of dataset GSE124114 were identified as outliers and removed for further analysis (see Supplementary File S1). The remaining high-quality data of the five datasets was combined into one dataset. This resulted in an integrated dataset consisting of 17,705 unique genes.

### Preprocessing of the Bovine Data

Within the bovine data, we first investigated the differential gene expression in the TM after exposure to dexamethasone compared with a control medium. Second, we investigated the differential gene expression in the TM between responders and nonresponders. Both obtained datasets comprised 25,794 unique genes.

### Pathway Overrepresentation Analysis

Pathway analysis was performed on the three datasets, which were obtained after performing the quality control and preprocessing steps. The integrated human dataset revealed 133 significantly changed pathways. The number of genes that fulfilled the criteria for significance (i.e., an absolute LogFC >0.58 and a *P* value < 0.05) was 829. The bovine dataset comparing TM cells treated with and without dexamethasone showed 190 significantly changed pathways, and the bovine dataset comparing responder and nonresponder TM-cells showed 136 significantly changed pathways. The complete results of the pathway analyses are shown respectively in Supplementary Files S2, S3, and S4.

### Clustering of the Pathway Results

The significantly changed pathways of each of the three performed pathway analyses were clustered into multiple functional categories. The clusters collagen, extracellular matrix (ECM), adhesion, WNT-signaling, inflammation, adipogenesis, and glocuse metabolism were found in the three datasets. For nuclear factor kappa-light-chain-enhancer of activated B cells (NF-κB), apoptosis, G protein-coupled receptor (GPCR), and oxidative stress (Table 2), it should be noted that multiple pathways involved in these clusters were just below the significance threshold in the other datasets. However, we should consider that the chosen threshold is an arbitrary cutoff value and should not be seen as a hard cutoff. Pathways just below the threshold are not per definition not involved and may still contain relevant changes or may have not been retrieved in each dataset due to a lack of power (Table 2). For example, some of the pathways aggregate information of multiple cellular processes. If only part of these processes are not involved in the pathogenesis of the investigated tissue, the complete pathway might be just below the chosen threshold level. However, specific pathways that contain very low numbers of measured genes may be just below the cutoff for significance due to a lower power. Cell cycle and senescence were only found in the responder versus nonresponder datasets, and not in the human or bovine dataset in which the effect of dexamethasone on the TM cells was investigated. The pathways involved in the categories for each of the analyses are presented in Supplementary File S5 (sheet 1–3).

**Table 2.** Overview of the Clusters Between the Different Datasets, with Additional Visualization for Clusters that were just Below the Cutoff

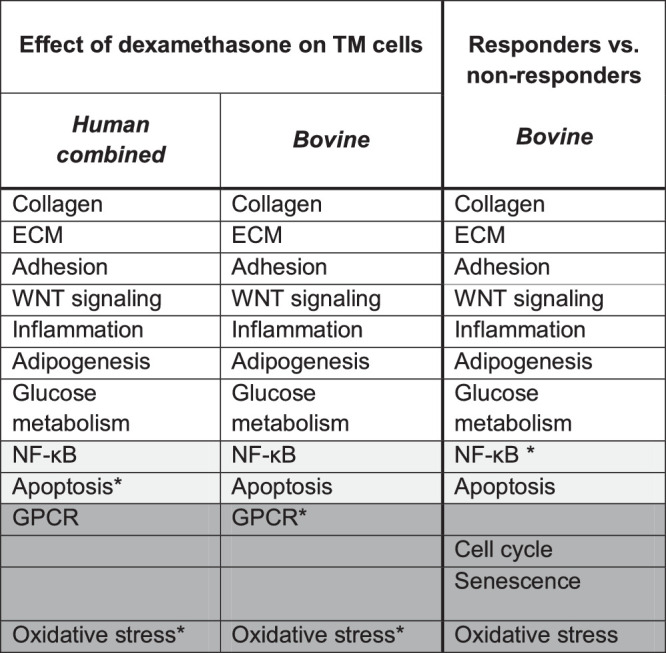		

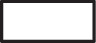
 Pathway clusters present in the three datasets.

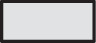
 Pathway clusters present in two out of three datasets.

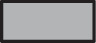
 Pathway clusters present in one out of three datasets.

* Clusters that were just below the cutoff for significance in the respective dataset to form a cluster but could be overall involved.

### Network Analysis

**Figure 2.**
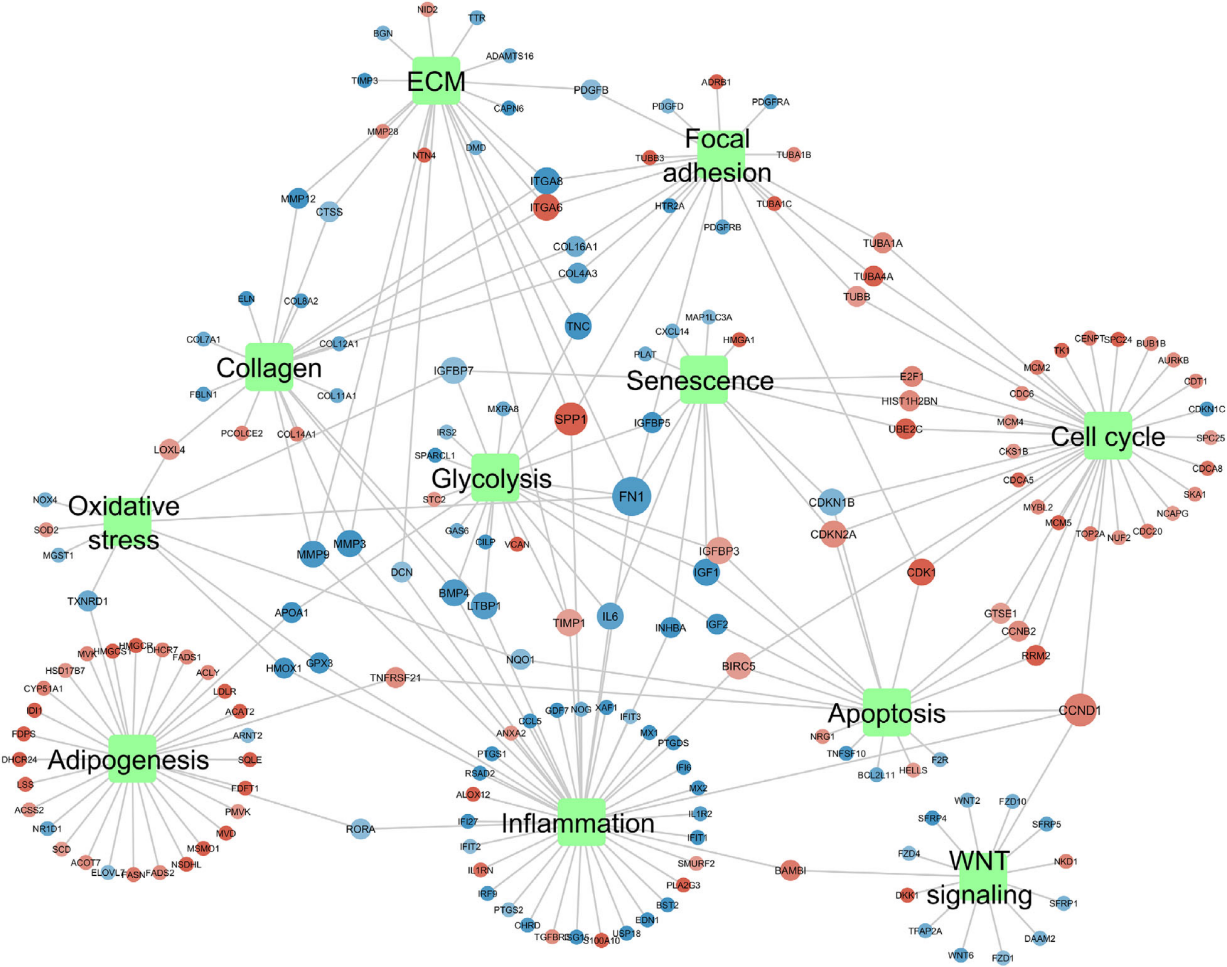
Network of the bovine data comparing TM cells of responders and nonresponders. *Green quadrangles* indicate the functional categories; each node represents a gene. *Red color* indicates upregulation, *blue color* indicates downregulation. The larger the node, the more clusters the gene is represented in.

To visualize the results after comparing the bovine responders and nonresponders, we created a network based on the genes within each of the pathways per functional cluster (Fig. 2). Only genes with an absolute logFC >0.58 are shown. The network illustrates that all the identified functional clusters are connected with each other. Multiple genes are shared between multiple clusters. An overview of the genes that are present in at least two clusters is shown in Table 3.

**Table 3.** Overview of the Functional Clusters Between the Different Datasets

Gene Symbol	Number of Clusters the Gene is Represented in	Involved Clusters
FN1	6	Inflammation, ECM, senescence, focal adhesion, oxidative stress, glucose metabolism
SPP1	4	Inflammation, ECM, focal adhesion, glucose metabolism
CCND1	4	Inflammation, cell cycle, apoptosis, WNT-signaling
IGFBP3	3	Senescence, apoptosis, glucose metabolism
IGF1	3	Senescence, apoptosis, glucose metabolism
MMP3	3	Inflammation, collagen, ECM
MMP9	3	Inflammation, collagen, ECM
LTBP1	3	Inflammation, collagen, glucose metabolism
BMP4	3	Inflammation, collagen, glucose metabolism
ITGA8	3	Collagen, ECM, focal adhesion
ITGA6	3	Collagen, ECM, focal adhesion
CDKN2A	3	Cell cycle, senescence, apoptosis
CDKN1B	3	Cell cycle, senescence, apoptosis
TIMP1	3	Inflammation, ECM, glucose metabolism
TNC	3	ECM, focal adhesion, glucose metabolism
CDK1	3	Cell cycle, apoptosis, focal adhesion
BIRC5	3	Inflammation, cell cycle, apoptosis
IGFBP7	3	Senescence, oxidative stress, glucose metabolism
IL6	3	Inflammation, senescence, glucose metabolism
E2F1	2	Cell cycle, senescence
HIST1H2BN	2	Cell cycle, senescence
UBE2C	2	Cell cycle, senescence
TUBA1A	2	Cell cycle, focal adhesion
TUBB	2	Cell cycle, focal adhesion
TUBA4A	2	Cell cycle, focal adhesion
RRM2	2	Cell cycle, apoptosis
GTSE1	2	Cell cycle, apoptosis
CCNB2	2	Cell cycle, apoptosis
COL4A3	2	Collagen, focal adhesion
COL16A1	2	Collagen, focal adhesion
CTSS	2	ECM, collagen
MMP12	2	ECM, collagen
GPX3	2	Inflammation, oxidative stress
HMOX1	2	Inflammation, oxidative stress
IGF2	2	Apoptosis, glucose metabolism
IGFBP5	2	Senescence, glucose metabolism
TNFRSF21	2	Apoptosis, adipogenesis
PDGFB	2	ECM, focal adhesion
LOXL4	2	Collagen, oxidative stress
DCN	2	Inflammation, ECM
NQO1	2	Apoptosis, oxidative stress
BAMBI	2	Inflammation, WNT-signaling
TXNRD1	2	Adipogenesis, oxidative stress
RORA	2	Adipogenesis, inflammation
INHBA	2	Inflammation, senescence
APOA1	2	Adipogenesis, glucose metabolism

## Discussion

Within this study, we identified seven functional pathway clusters that were significantly changed in both TM cells exposed to dexamethasone versus a control medium, as well as in TM cells of responders versus nonresponders: collagen, ECM, adhesion, WNT-signaling, inflammation, adipogenesis, and glucose metabolism. In addition, the functional clusters of NF-κB, apoptosis, GPCR, and oxidative stress were just below the cutoff for significance in some of the datasets, but this does not rule out their potentially relevant involvement. In contrast, it is remarkable that the pathways within the functional categories cell cycle and senescence were highly significant in the bovine responder versus nonresponder data, and nonsignificant in the other datasets. Therefore these functional clusters are discussed in more detail later.

**Figure 3.**
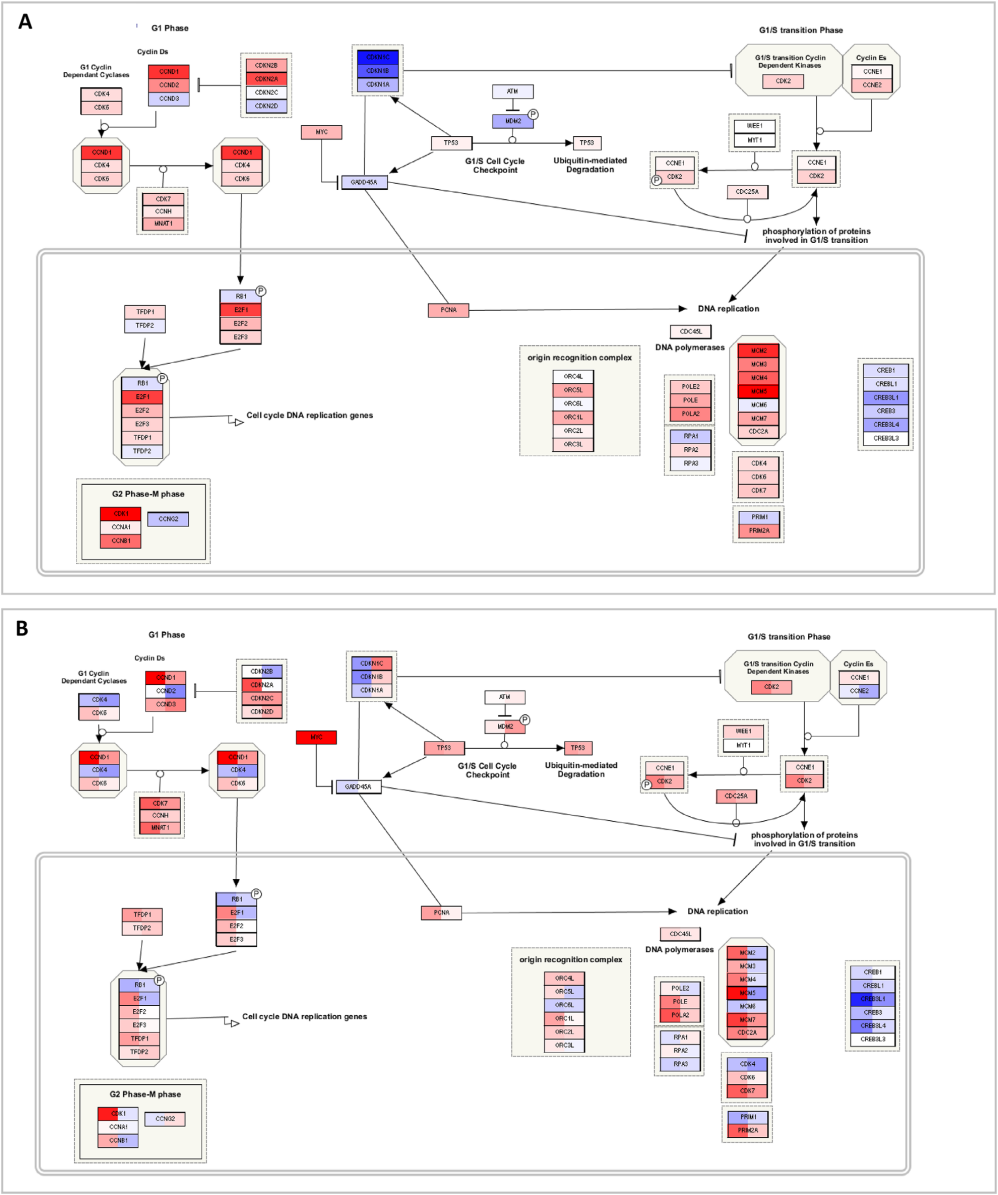
Visualization of the cell cycle pathway from WikiPathways. (**A**) Visualizing the results after comparing the bovine responders versus nonresponders. Every *colored rectangle* represents a gene that had been measured in the bovine dataset. *Red color* indicates upregulation, *blue color* indicates downregulation. The higher the intensity of the color, the higher the up- or downregulation. (**B**) Visualizing the LogFC of the responders and the nonresponders as calculated in Formula 1. Every *colored rectangle* represents a gene that had been measured in the bovine dataset. The first part of the rectangle represents the responders, and the second part of the rectangle represents the nonresponders. *Red color* indicates upregulation, *blue color* indicates downregulation. The higher the intensity of the color, the higher the up- or downregulation.

The network shows that most genes involved in the category cell cycle are upregulated after comparing responders and nonresponders. To obtain a better understanding, we investigated the pathways involved in this cluster separately (see for example Fig. 3A). Within this pathway CCND1 and CCND2, known to drive the G1/S phase transition by binding with multiple cyclin-dependent kinases (CDKs), are upregulated. CDKs are necessary to regulate the progression through the cell cycle and are also upregulated. In addition, CDKN1A, CDKN1B, and CDKN1C normally inhibit CDKs but are here downregulated. Therefore the gene expression of the mentioned genes suggest an increased activity of the cell cycle. As cell cycle was only significantly changed after comparing responders and nonresponders and not after comparing exposure of dexamethasone versus a control medium, this might suggest that the pathways or multiple genes within this functional cluster behave in opposite ways in responders and nonresponders. To check this hypothesis, we visualized the cell cycle pathway with the gene expression values of responders and nonresponders, as calculated in Formula 1 (Fig. 3B). As expected, the genes within these pathways are expressed in the opposite direction or have relatively large differences in their level of expression.

It is known that corticosteroids influence the cell cycle. However, the effects have been suggested to be cell specific and differ between the dosage and types of corticosteroids. For example a study investigating the effect of different types of corticosteroids on hyperproliferant keratinocytes found that all types of corticosteroids reduced the cell proliferation.^32^ However, cultured corneal epithelial cells showed an increased cell proliferation when exposed to dexamethasone less than 10^−6^ M, and inhibition of the proliferation at concentrations of more than 10^−4^.^33^ In addition, we now found that the activation of the cell cycle in the TM is different between responders and nonresponders.

Previous studies showed that cell proliferation is inhibited in TM cells after exposure to corticosteroids. In this study, we found this process not to be significantly changed after exposure to corticosteroids. However, as stated earlier, we now investigated the difference between responders and nonresponders and found a clear upregulation of cell proliferation in responders compared with nonresponders. This suggests that there is a different reaction to corticosteroids in responders compared with nonresponders and shows the need of further investigation with human TM tissue of identified responders and nonresponders instead of solely TM cells exposed to corticosteroids.

**Figure 4.**
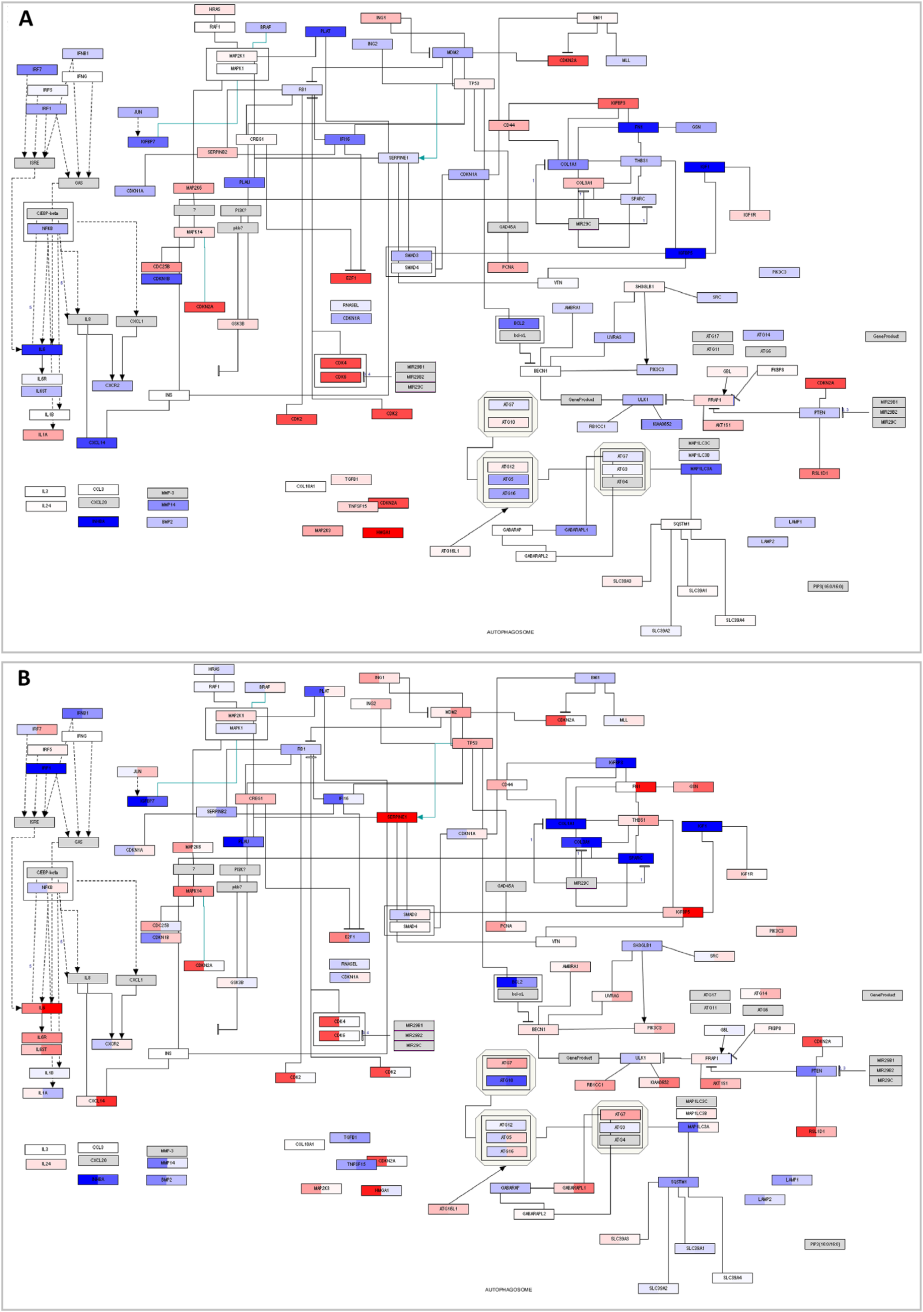
Visualization of the senescence pathway from WikiPathways. (**A**) Visualizing the results after comparing the bovine responders versus nonresponders; every *colored rectangle* represents a gene that had been measured in the bovine dataset. *Red color* indicates upregulation, *blue color* indicates downregulation. The higher the intensity of the color, the higher the up- or downregulation. (**B**) Visualizing the LogFC of the responders and the nonresponders as calculated in Formula 1; every *colored rectangle* represents a gene that had been measured in the bovine dataset. The first part of the rectangle represents the responders, and the second part of the rectangle represents the nonresponders. *Red color* indicates upregulation, *blue color* indicates downregulation. The higher the intensity of the color, the higher the up- or downregulation.

Senescence was also found to be involved in corticosteroid-induced OHT. Corticosteroids have been shown to have enhancing or inhibiting effects on senescence.^34^^–^^36^ In contrast to the cell cycle, it is difficult to define a certain overall up- or downregulation of the identified pathways that are involved in this functional category (Fig. 4A). However, when visualizing the LogFC of the responders and nonresponders on these pathways, multiple genes are expressed in the opposite direction (Fig. 4B) or show large differences in gene expression. A gene of particular interest might be UBE2C, as it is not only significantly upregulated in responders and downregulated in nonresponders, it is also shared by the cell cycle cluster, as shown in the network.

Furthermore, we found the functional category senescence to be involved in the molecular pathogenesis of POAG as well.^18^ It is known that patients with POAG are more susceptible to develop corticosteroid-induced OHT. The other way around, patients who had a corticosteroid response in the past are at risk to develop POAG.^1^^,^^6^^,^^37^^–^^40^

Multiple processes, such as ECM, focal adhesion, collagen, and WNT-signaling, have been described to be involved in the pathogenesis of corticosteroid-induced glaucoma.^2^^,^^6^^–^^13^^,^^41^

**Figure 5.**
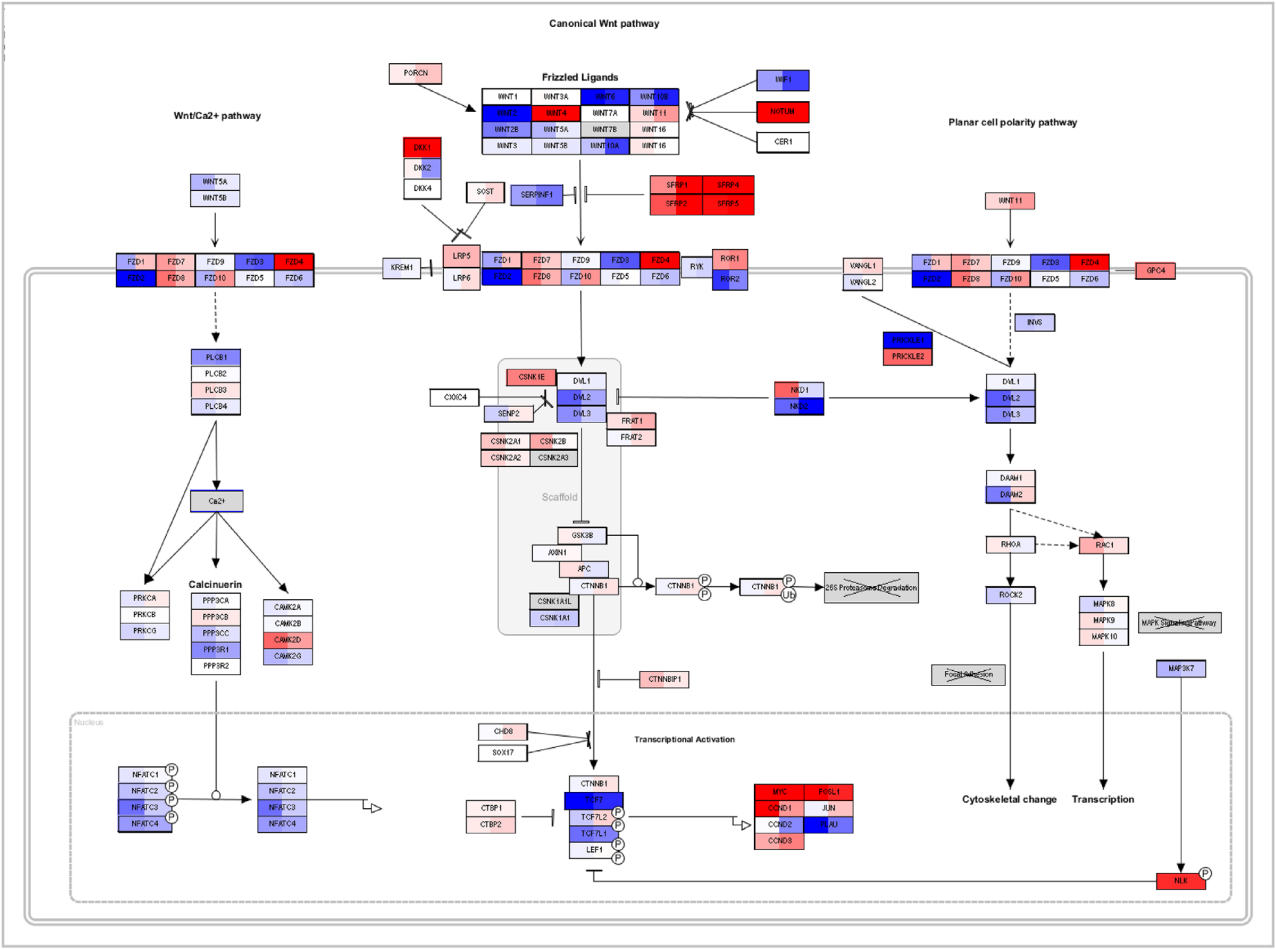
WNT-signaling pathway from WikiPathways after visualizing the LogFC of the responders and the nonresponders as calculated in Formula 1; every *colored rectangle* represents a gene that had been measured in the bovine dataset. The first part of the rectangle represents the responders, and the second part of the rectangle represents the nonresponders. *Red color* indicates upregulation, *blue color* indicates downregulation. The higher the intensity of the color, the higher the up- or downregulation.

We found these clusters to be significantly changed in all three datasets. This indicates that the differences in gene expression profiles, for these clusters, between responders and nonresponders are not as extensive, as seen in cell cycle and senescence. Indeed, most genes involved in these pathways do not show opposites in gene expression (up- or downregulation) but differed in the intensity of the gene expression (Fig. 5). Nevertheless, despite the fact that these clusters show differences in gene expression rather than opposites, they are also likely to be involved in the pathogenesis of corticosteroid-induced glaucoma and need further investigation.

We created a network comparing bovine eyes with and without a corticosteroid response. Notably, the visualized genes involved in the separate clusters are largely homogeneous up- or downregulated. Multiple genes are shared between multiple clusters and might be of special interest. However, fibronectin 1 (FN1) is shared by the largest number of clusters. Previous research showed that the gene expression of FN1 is increased in human and bovine TM cells after exposure to dexamethasone. Interestingly, in the responders FN1 is almost not expressed, and in the nonresponders it is significantly upregulated. Consequently, after comparing responders and nonresponders, as shown in the network, FN1 gene is downregulated. Bermudez et al.^14^ already addressed this finding and performed a western blot on FN1. They found a significant higher expression in responders compared with nonresponders after exposure to dexamethasone.

Raghunathan et al.^3^ performed immunocytochemistry on human TM cells treated with dexamethasone and found fibronectin to be deposited as an organized fibrillar sheet. This was not observed in human TM cells exposed to a control medium. In another study of the group of Raghunathan et al.^42^ the same results were reported. Li et al.^43^ found similar results after exposing the TM of mice to dexamethasone. In contrast to this finding, Raghunathan et al.^3^ reported no significant difference in the protein expression of fibronectin between human TM cells exposed to dexamethasone or those exposed to a control medium. In a similar experiment by Shan et al.,^44^ the protein expression of fibronectin was also not significantly changed in human TM cells exposed to dexamethasone when compared with TM cells exposed to a control medium. However, they reported that the protein expression of fibronectin was decreased in human TM cells exposed to prednisolone when compared with controls. Bollinger et al.^45^ investigated both glaucomatous and nonglaucomatous human TM samples. They also found that the average relative protein abundance over all dexamethasone-treated TM cell samples (glaucomatous and nonglaucomatous together) did not show a significant change in fibronectin protein expression. In contrast, Honjo et al.,^46^ Peng et al.,^47^ Filla et al.,^48^ and Zhou et al.^49^ reported that the protein expression of fibronectin was significantly increased after exposing human TM cells to dexamethasone. Additionally, Li et al.^50^ reported the fibronectin protein secretion to be significantly increased in four human TM cell strains but to be decreased in one human TM cell strain after exposure to dexamethasone (both after 1 and 4 weeks of exposure). Steely et al.^51^ investigated the fibronectin gene expression in one glaucomatous and three nonglaucomatous human TM cell strains. They found a significant elevation of fibronectin gene expression in the glaucomatous TM cells exposed to dexamethasone and in two of the nonglaucomatous cell strains but no increase in one of the nonglaucomatous strains. Additionally to human protein expression studies, Patel et al.^52^ performed an immunohistochemical analysis on the TM of wild type mice after treatment with periocular dexamethasone-acetate injections and found an increased protein expression of fibronectin when compared with wild type mice treated with a control medium. Wang et al.^53^ exposed TM cells of rats to different concentrations of dexamethasone and found the protein expression of fibronectin to be increased as well when compared with controls. Based on the results of the earlier-mentioned studies, the effect of dexamethasone-treatment of TM cells on the protein expression of fibronectin is incongruent.

In addition, multiple human transcriptomics studies also investigated the gene expression of FN1 after exposing TM cells to corticosteroids: Fan et al.,^6^ Nehmé et al.,^10^ Kwon et al.,^8^ Matsuda et al.,^9^ and Faralli et al.^31^ They all found fibronectin to be upregulated. As these studies were included in our study, the average logFC of fibronectin after exposing TM cells to corticosteroids was significantly upregulated (average LogFC: 0.68; average weight: 4.33; average *P* value: 0.00005).

Raghunathan et al.^3^ stated that an increased activation of contractility machinery and perhaps altered integrin binding in dexamethasone-treated cells may contribute to the reorganization of deposited fibronectin. Therefore in accordance with the suggestion by Bermudez et al.,^14^ the differences might be caused by posttranslational processes.

Additionally, it is remarkable that in both the study of Li et al.^50^ and Steely et al.,^51^ some of the included strains showed an increased fibronectin protein expression and others a decreased expression or even no change in expression. As a steroid response is very common (one out of three patients), it is possible that the steroid response status of a patient might explain these differences. Currently, to our best knowledge, only two studies investigated the protein expression of fibronectin in steroid responders versus nonresponders. Both studies used bovine TM tissue.^14^^,^^54^ The study of Bermudez et al.^14^ reported an increased expression of fibronectin protein levels in responders but not in nonresponders.^14^ The study of Mao et al.^54^ used an anterior segment perfusion system and found the fibronectin protein expression to be induced in the TM of three out of eight responders and in one out of six nonresponders, this difference, however, was not statistically significant (*P* > 0.5). Therefore the role of responders and nonresponders in the protein expression of fibronectin needs further investigation.

One of the strengths of this study is that we performed a systematic method in which all relevant publicly available gene expression data were used. This enabled us to integrate and build on existing knowledge. However, as in a meta-analysis, a critical appraisal of the included studies and their quality is necessary. A recent review of Keller et al.^55^ defined the induction of myocilin (MYOC) after exposure to dexamethasone to be a reliable marker for TM tissue.^56^ We found MYOC to be highly expressed in each of the five included human studies, which indicates that the investigated tissue indeed is TM tissue. In the bovine study, MYOC was not highly expressed. However, multiple studies found contradictive results regarding the expression of MYOC after exposing bovine TM tissue to dexamethasone.^13^^,^^57^ Differences in breed have been suggested to play a role.^55^

Furthermore, the age of the donors in the study ranged between 3 months and 72 years (see characteristics in Table 1). The consensus of Keller et al.^55^ recommends using donors younger than age 60 years, however, donors older than 60 years may also provide adequate primary TM cell cultures. Within this study, only one donor was older than 60 years and included for further analysis. We did, however, exclude a 3-month-old donor (dataset GSE16643) as the development of the TM continues in the postnatal period.^58^^–^^60^

Some issues need to be addressed. Ideally, gene expression data of TM tissue specifically derived from human corticosteroid responders would be the tissue of primary choice. However, to our best knowledge, these data are not available. Therefore we used the gene expression data of bovine TM cells of responders and nonresponders. The bovine TM outflow tissue is known to be morphologically different from human TM tissue.^61^ However, bovine eyes are known to develop a corticosteroid response after treatment with corticosteroids, and similar to human subjects this response declines after discontinuation of the corticosteroids.^62^ The development of OHT after exposure to dexamethasone was also observed in perfusion-cultured bovine anterior segments, which was used by Bermudez et al.^14^ Furthermore, the physiology of the aqueous humor formation resembles that of human subjects, as both have higher concentrations of chloride compared with plasma, and the chloride transport is in both species inhibited by carbonic anhydrase inhibitors.^63^^,^^64^ The earlier described findings imply that bovine eyes and the perfusion-cultured model used by Bermudez et al.^14^ are suitable for investigating corticosteroid-induced glaucoma. In addition, the functional clusters we identified for the human and bovine data after investigation of the effect of corticosteroids on the TM are the same that validates the used model.

We used the human pathway collections of WikiPathways, KEGG, and Reactome for pathway analysis of the bovine data instead of the bovine (*Bos taurus*) pathways. This was done as available bovine pathways are commonly converted from human pathways and are therefore not likely to add any new information. The human pathway collection is also more extensive than the bovine collection. Additionally, consistently using the human pathway collection allowed comparing the results of the human and bovine data with each other.

We could not alter the cutoff values for a corticosteroid response. Bermudez et al.^14^ defined a corticosteroid response as the average IOP minus the baseline IOP to be equal or higher than 2.82 mm Hg. IOP was recorded every minute, and the average was calculated every 24 hours. Multiple definitions for corticosteroid response have been defined but one of the most frequently used definitions is a one-time increase of 6 mm Hg over baseline.^37^ It is, however, known that diurnal IOP fluctuations can cause this difference in IOP, which might result in the unjustified diagnosis of a corticosteroid response.^65^^–^^67^ Bermudez et al.^14^ used a less stringent cutoff, however, they averaged the IOP over 24 hours, which might make the elevation more robust. Also, because the exposure period was short, a lower cutoff is more valuable because the 6 mm Hg or higher also reflects long-term exposure to corticosteroids. A low but early increase in IOP could identify these subjects more correctly. In addition, the results show clear differences in responders and nonresponders based on gene expression and molecular processes, which strengthens the fact that responders and nonresponders were identified correctly. Nevertheless, it is of value to study the molecular processes that cause early or late corticosteroid response.

Additionally, there are some differences on study level between the included datasets. Different control mediums were used, and the time of exposure to corticosteroids ranged between 1 and 14 days across the included datasets (Table 1). It is likely that both the use of different control mediums and a different time of exposure to corticosteroids cause differences in the transcriptome response. However, the other way around, consistently using the same control medium, without the exact knowledge on how this could affect gene expression, or the same time of exposure within every study, could also mask some of the results as the transcriptome response might consistently under- or overexpress some genes. Nonetheless, as we were concerned that the differences between datasets could influence the results, we performed pathway analyses on the gene expression data of the separate datasets (not shown). This showed no major differences between the identified significantly changed pathways and processes between the different datasets. This indicates that the differences between the studies did not influence the results on pathway and process level. Nonetheless, to obtain results that are less dependent on study differences of individual studies and concise, we combined the high-quality and preprocessed gene expression datasets of the included studies for the reported analyses.

## Conclusions

The systematically performed approach allowed the identification of the functional processes of cell cycle and senescence to be highly likely involved in the pathogenesis of corticosteroid-induced OHT. Other processes, such as collagen, ECM, adhesion, and WNT-signaling, behave differently between responders and nonresponders as well. However, as these differences are mainly based on differences in intensities of gene expression rather than opposites, further investigation of these processes are needed. These pathways and their involved genes, and maybe especially the genes shared between the identified processes after comparing responders and nonresponders, are of interest for drug targeting.

## Supplementary Material

Supplement 1

Supplement 2

Supplement 3

Supplement 4

Supplement 5
