# Supplementary material for: The Molecular Processes in the Trabecular Meshwork After Exposure to Corticosteroids and in Corticosteroid-Induced Ocular Hypertension
Source: Invest Ophthalmol Vis Sci. 2020 Apr 18;61(4):24. doi: 10.1167/iovs.61.4.24 (PMC7401422; doi:10.1167/iovs.61.4.24)
Supplement: Supplement 1 [file iovs-61-4-24_s001.pdf]

Appendix 1. Results of the quality control of the five included microarray datasets

Fan et al. (GSE6298)

Two-color cDNA microarrays were used causing the control data to be embedded in the data presented in the dataset. Therefore, vector-ratios (instead of intensities) were used during the quality control and Euclidean was used instead of Pearson. We first performed a quality control on the raw data as provided by the authors; however, based on the results of the quality control, we concluded that this data was already normalized. Therefore, only the normalized data were available and subsequently the quality before and after normalization couldn't be compared. The boxplot (Supplementary figure 1A) showed similar intensities between the different samples, however the intensities of sample TA1_2 en TA1_3 are respectively slightly lower and higher compared to the other samples. The density histogram (Supplementary figure 1B) of signal intensities also showed some small differences between the samples. In the hierarchical clustering (Supplementary figure 1C) and the PCA-plot (Supplementary figure 1D), the samples treated with dexamethasone clustered together. However, the triamcinolone samples clustered in a mixed way. As the dexamethasone samples were consistently correlated with each other and showed similar intensities in all the plots, these samples could be used for further analysis.

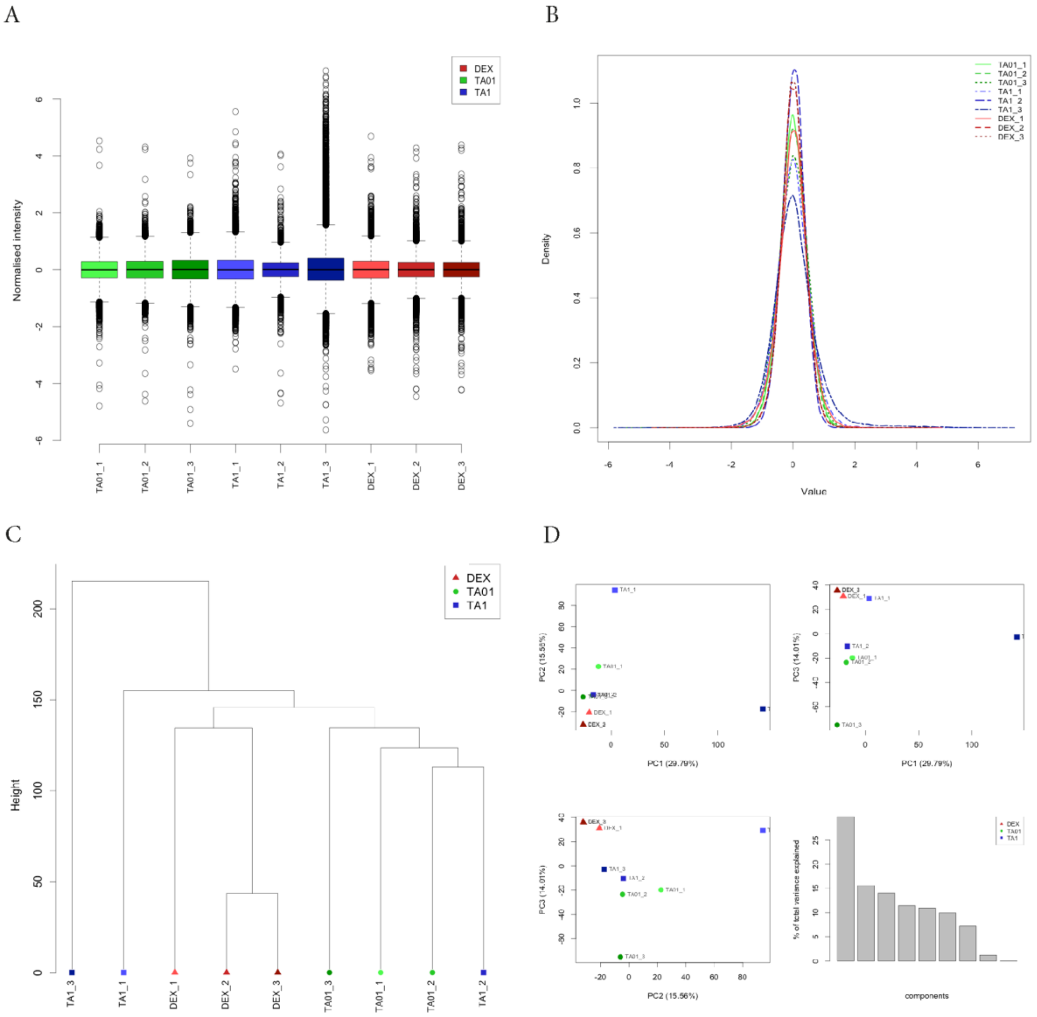

Supplemental figure 1. Quality control of dataset GSE6298

A. Boxplot of the signal intensities; B. Density histogram of the signal intensities; C. Cluster dendrogram; D. PCA-analysis

Nehmé et al. (GSE16643)

For this study, only the normalized data was provided by the researchers. However, preprocessing steps showed that it was not normalized yet and is therefore referred to as raw data in the following section. In order to normalize the data, we performed quantile normalization. In the plots of the raw data, sample FA_93_A clearly behaved as an outlier in all the plots. The boxplot and density histogram showed that it has a lower intensity compared to the other samples (Supplementary figure 2.1A and 2.1B). In the cluster dendrogram (Supplementary figure 2.1C), sample FA_93_A clustered as an outlier. The correlation plot (Supplementary figure 2.1D) showed that sample FA_93_A has a low correlation when compared to the other samples (<0.968) and also in the PCA-plot (Supplementary figure 2.1E), this sample behaved divergent. Therefore, we removed sample FA_93_A from the database and repeated the QC. Thereafter, the boxplot and density histogram of the raw data showed some small variances between the intensities of the samples (Supplementary figure 2.2A and 2.2B) which disappeared after normalization (Supplementary figure 2.3A and 2.3B). The cluster dendrogram showed that the samples cluster per individual. Further, the samples treated with dexamethasone and the controls cluster in the dendrogram in a mixed way (Supplementary figure 2.2C). This remained after performing normalization techniques (Supplementary figure 2.3C). The correlation plot showed a high correlation (> 0.97) between the samples before (Supplementary figure 2.2D) and after the normalization (Supplementary figure 2.3D). The PCA-plots showed that the samples are mainly clustered per individual (Supplementary figure 2.2E), this is even more clear after the normalization (Supplementary figure 2.3E). The repeated QC showed an overall high quality of the remaining data with no other deviating samples. Based on the findings above, we concluded that this dataset is of high quality. However, as the data showed a clear distinction between the three month and the 35 year old donor and as there is little known about the developmental processes in the TM of newborns, we only

used the data of the 35 year old donor.

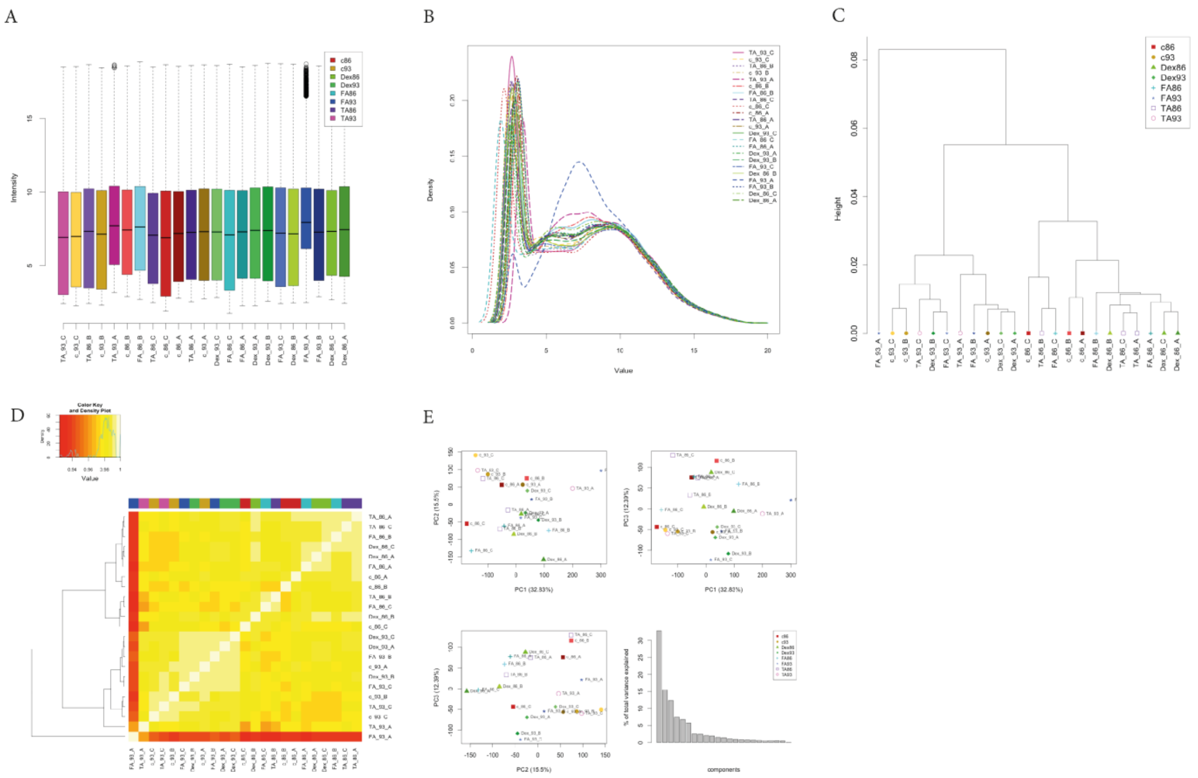

Supplemental figure 2.1 Quality control of dataset GSE16643: raw data, sample FA_93_A included
A. Boxplot of the signal intensities; B. Density histogram of the signal intensities; C. Cluster dendrogram; D. Correlation plot; E. PCA-analysis

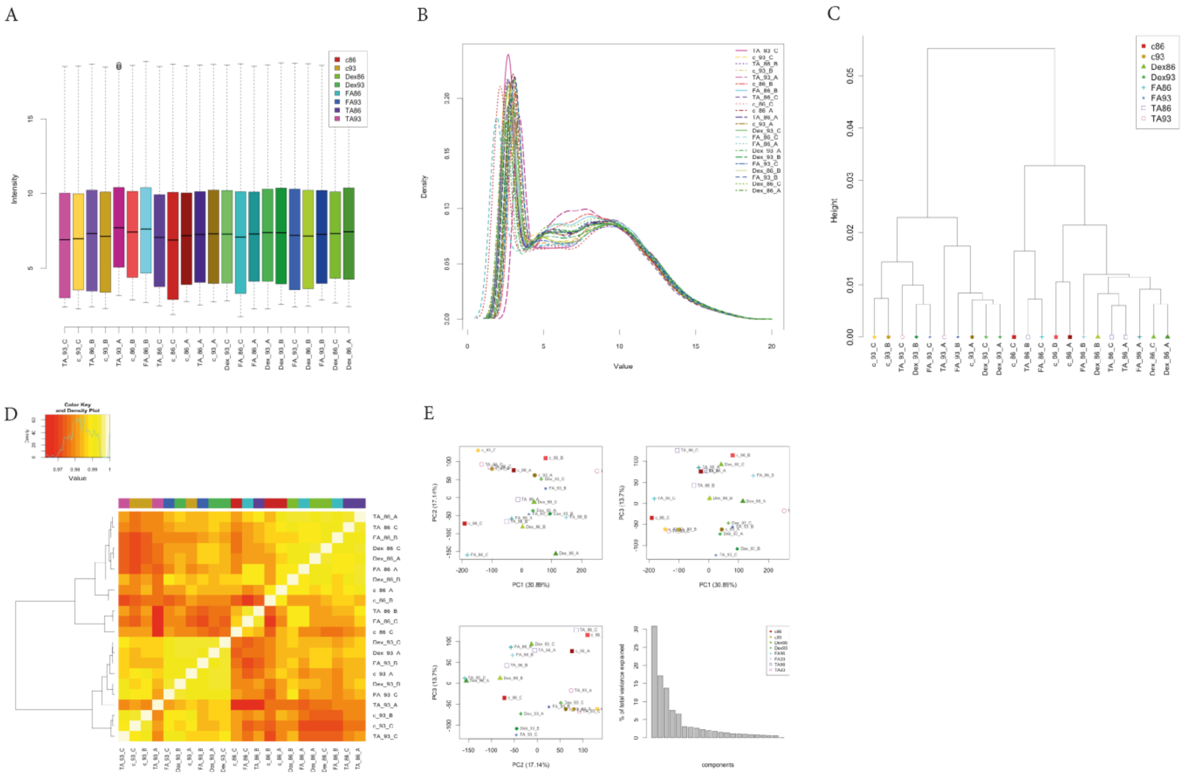

Supplemental figure 2.2 Quality control of dataset GSE16643: raw data, sample FA_93A excluded
A. Boxplot of the signal intensities; B. Density histogram of the signal intensities; C. Cluster dendrogram; D. Correlation plot; E. PCA-analysis

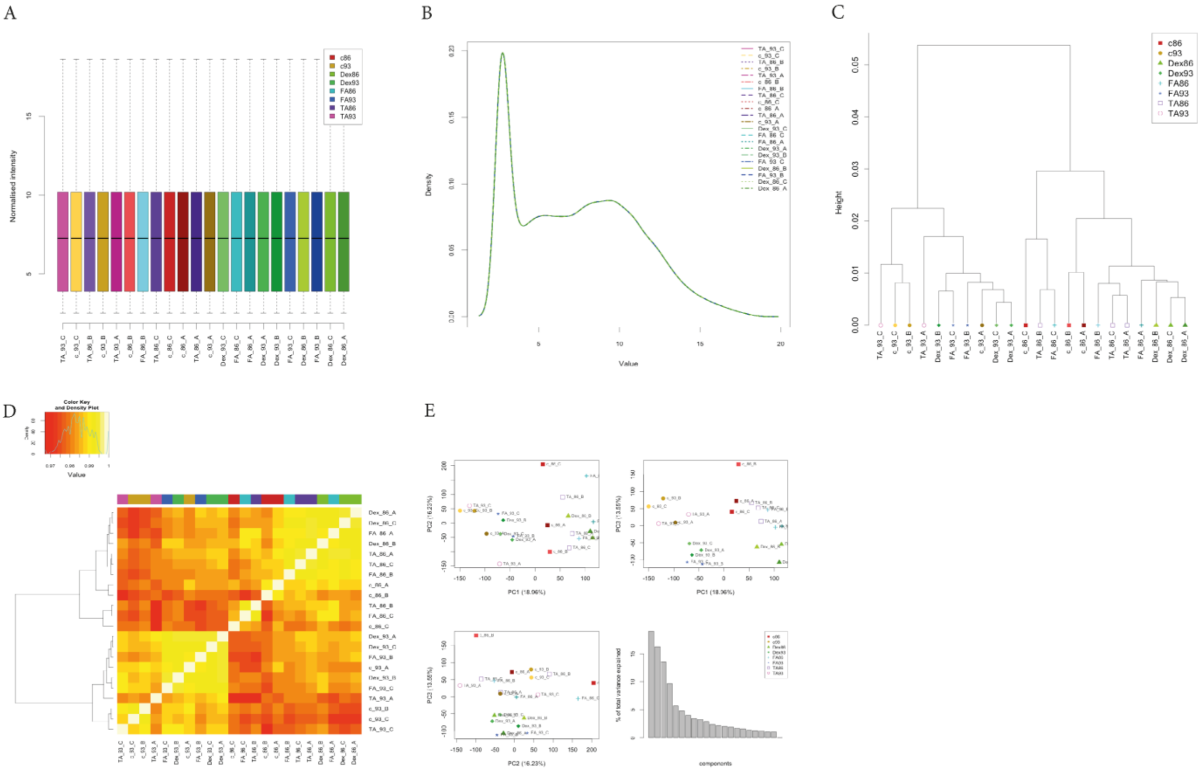

Supplemental figure 2.3 Quality control of dataset GSE16643: normalised data, sample FA_93A excluded
A. Boxplot of the signal intensities; B. Density histogram of the signal intensities; C. Cluster dendrogram; D. Correlation plot; E. PCA-analysis

Matsuda et al. (GSE65240)

For the RNA-analysis, two types of micro-arrays were used (GPL17077 and GPL14550). We investigated the probes on both arrays and we found that the probes are identical; however, probe GPL17077 was more recent. Therefore, we used this probe as it had the newest versions of the annotations. We first performed a quality control on the raw data as provided by the authors; however, based on the results of the quality control we concluded that this data was already normalized. Accordingly, the quality before and after normalization couldn't be compared.

The boxplot and density histogram showed that the intensities between the samples were comparable (Supplementary figure 3A and 3B). In the hierarchical clustering the samples of donor three cluster separately from the other two donor samples (Supplementary figure 3C). In the PCA plot, the control and the dexamethasone samples clustered separately (Supplementary figure 3D). The correlation plot showed a high correlation (>0.94), despite a slightly lower correlation of sample three when compared to the other two samples (Supplementary figure 3E). Because the samples of donor three behaved just slightly different compared to the other samples and only in some of the plots, the samples were maintained.

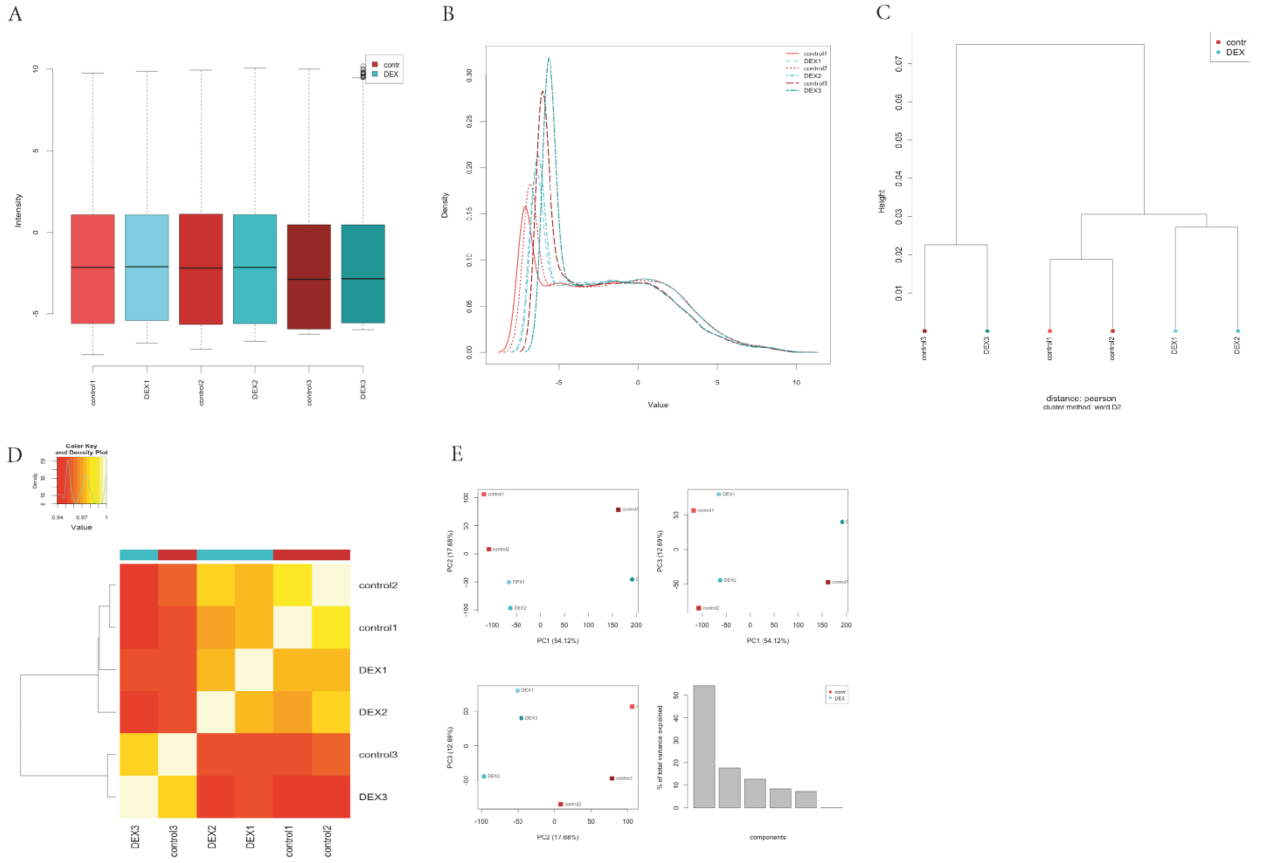

Supplemental figure 3. Quality control of dataset GSE62540: normalised data
A. Boxplot of the signal intensities; B. Density histogram of the signal intensities; C. Cluster dendrogram; D. Correlation plot; E. PCA-analysis

Kwon et al. (GSE37474)

For this study, both the raw and the normalized data were available and therefore, the quality before and after normalization were compared. The boxplot and density histogram of the raw data showed some small variances between the intensities of the samples (Supplementary figure 4.1A). After normalization, the intensities between the samples were comparable (Supplementary figure 4.2A). In the hierarchical clustering the samples of donor one and four clustered in a mixed way in both the raw and normalized plots (Supplementary figure 4.1C and 4.2C). The correlation plot of the raw data showed a high correlation (>0.91), despite a slightly lower correlation of sample five and three with the other samples (Supplementary figure 4.1D). The correlation plot of the normalized data showed a high correlation (>0.93) as well and now only sample five has a slightly lower correlation with the other samples (Supplementary figure 4.2D). In the PCA plot, both before after normalization, the control and the dexamethasone samples clustered in a mixed way (Supplementary figure 4.1E and 4.2E). Further, donor three and five clustered separately from the other samples. However, as the samples of donor three and five only deviated slightly and not consistently in all the plots, all the samples were included for further analysis.

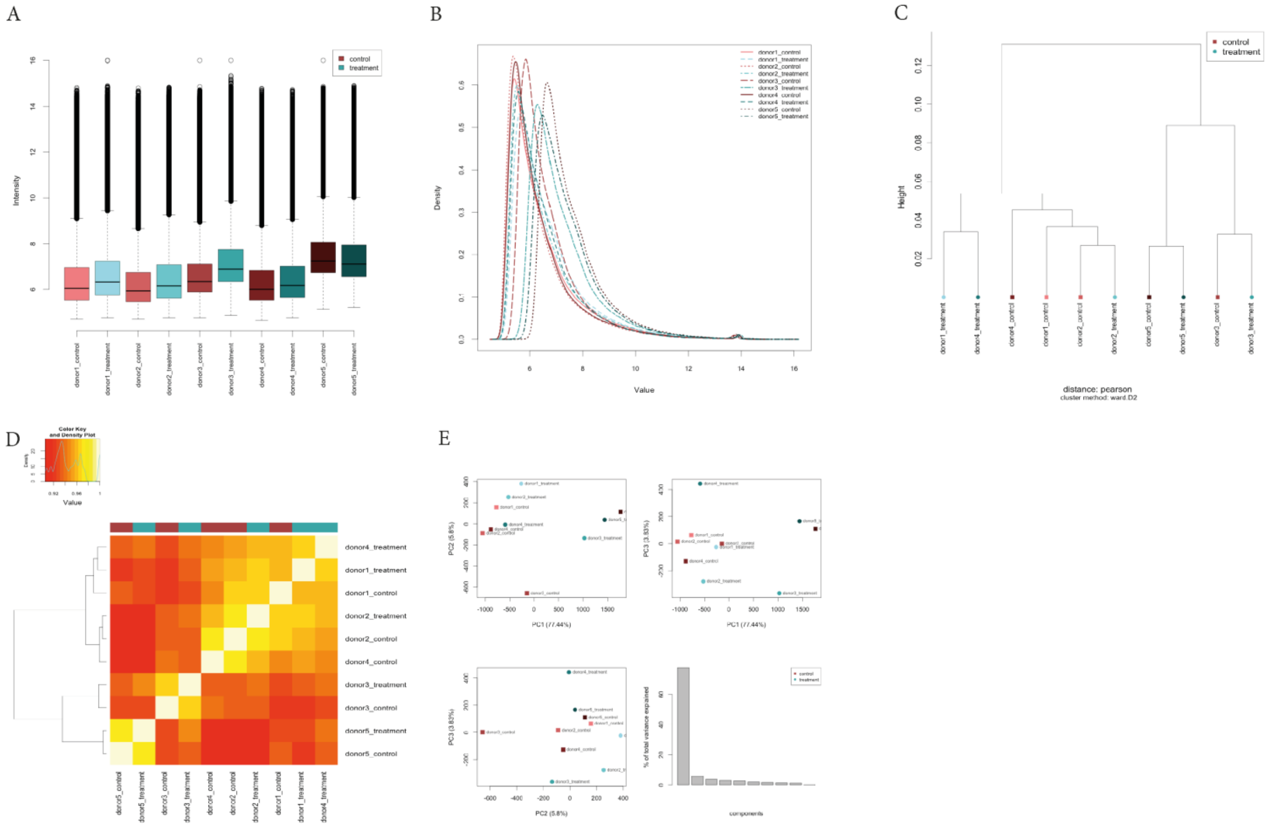

Supplemental figure 4.1 Quality control of dataset GSE37474: raw data
A. Boxplot of the signal intensities; B. Density histogram of the signal intensities; C. Cluster dendrogram; D. Correlation plot; E. PCA-analysis

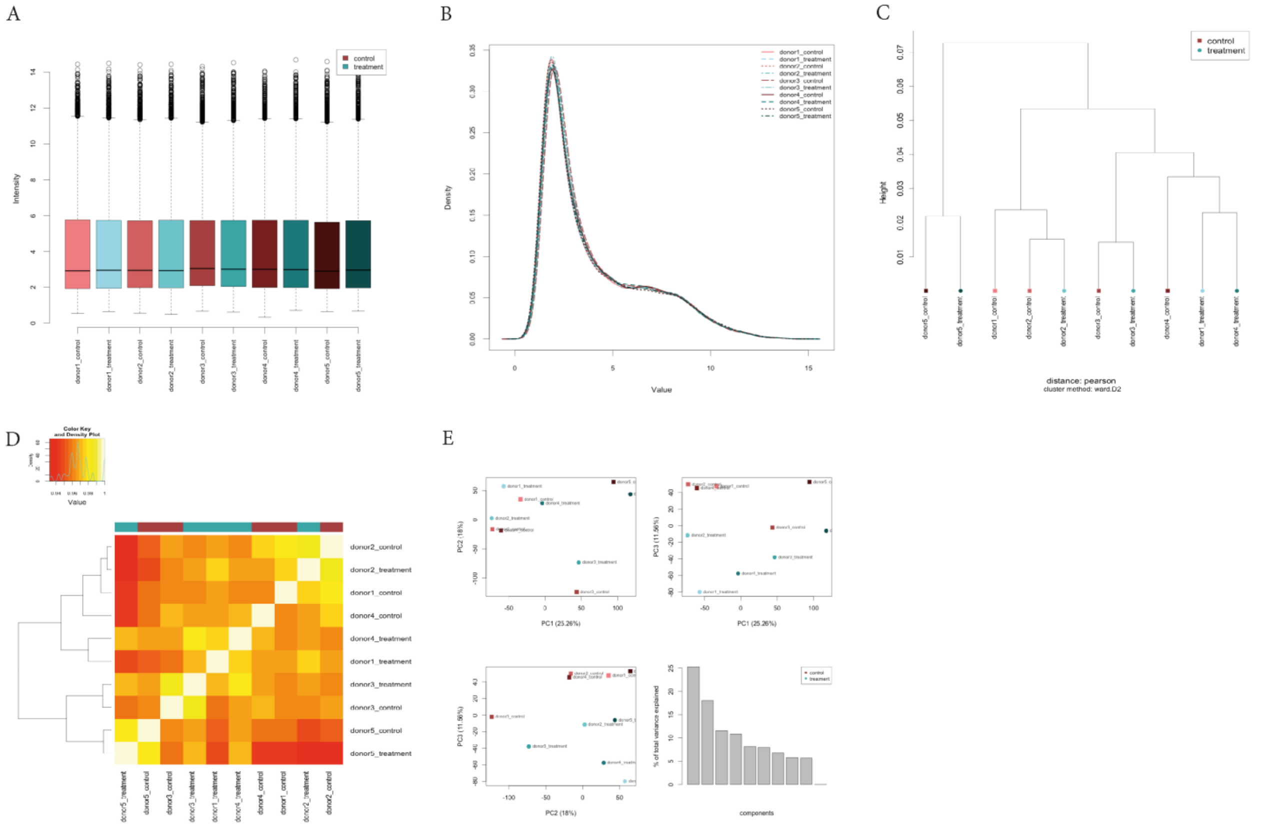

Supplemental figure 4.2 Quality control of dataset GSE37474: normalised data
A. Boxplot of the signal intensities; B. Density histogram of the signal intensities; C. Cluster dendrogram; D. Correlation plot; E. PCA-analysis

Peters et al. (GSE124114)

We used the raw data as provided by the authors and performed quantile normalization ourselves. Following we conducted a quality control on both the raw and normalized data. Within the plots of the raw data, four samples clearly behaved as outliers. TM2_DEX10 had a lower intensity when compared to the other samples (Supplementary figure 5.1A) and within the cluster dendrogram it clustered separately from all the other samples (Supplementary figure 5.1C). In the correlation plot, its correlation to the other samples was really low (0.84) (Supplementary figure 5.1D) and it was also an extreme outlier in the PCA plots (Supplementary figure 5.1E). Three other samples, TM2_DEX1, TM_2EtOH1a, and TM2_EtOH9 behaved as outliers as well. Their intensity and correlation was comparable with the other samples; however, they also clustered separately from the other samples in the cluster dendrogram and in the PCA plots (Supplementary figure 5.1C and 5.1E). Therefore, we removed the above-mentioned samples from the database and repeated the QC on both the raw and normalized data. The boxplot and density histogram of the raw data showed some small variances between the intensities of the samples (Supplementary figure 5.2A and 5.2B) which disappeared after normalization (Supplementary figure 5.3A and 5.3B). The cluster dendrogram of both the raw and normalized data showed that the samples cluster per TM cell isolate (TM4 and TM2). The cluster plot of the raw data also showed that the samples treated with a control medium and dexamethasone clustered in a mixed way, however, this improved after normalization (Supplementary figure 5.2C and 5.3C). The correlation plot showed a high correlation (0.92) between the samples before normalization (Supplementary figure 5.2D) which further increased after the normalization (0.96) (Supplementary figure 5.3D). The PCA-plots showed that the samples were mainly clustered per TM cell isolate (Supplementary figure 5.2E). This was even more clear after the normalization (Supplementary figure 5.3E). The repeated QC showed an overall high quality of the remaining data with no other

consistently deviating samples. Based on the findings above, we concluded that this dataset was of high quality.

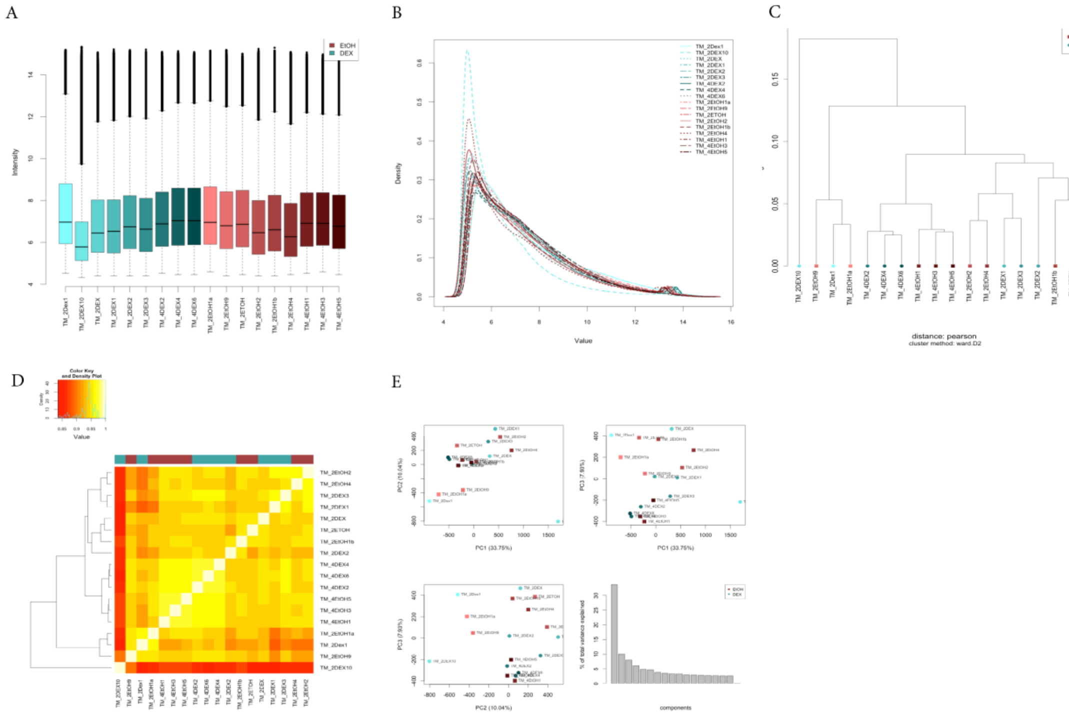

Supplemental figure 5.1 Quality control of dataset GSE124114: raw data, all samples included
A. Boxplot of the signal intensities; B. Density histogram of the signal intensities; C. Cluster dendrogram; D. Correlation plot; E. PCA-analysis

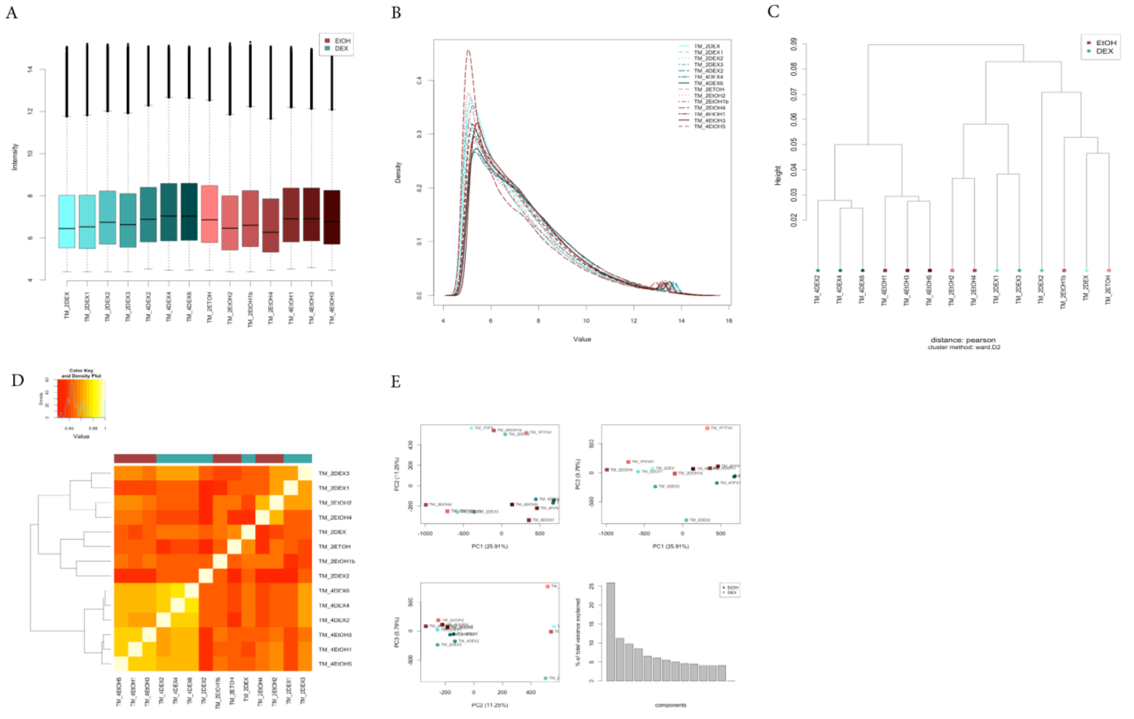

Supplemental figure 5.2 Quality control of dataset GSE124114: raw data, outliers removed
A. Boxplot of the signal intensities; B. Density histogram of the signal intensities; C. Cluster dendrogram; D. Correlation plot; E. PCA-analysis

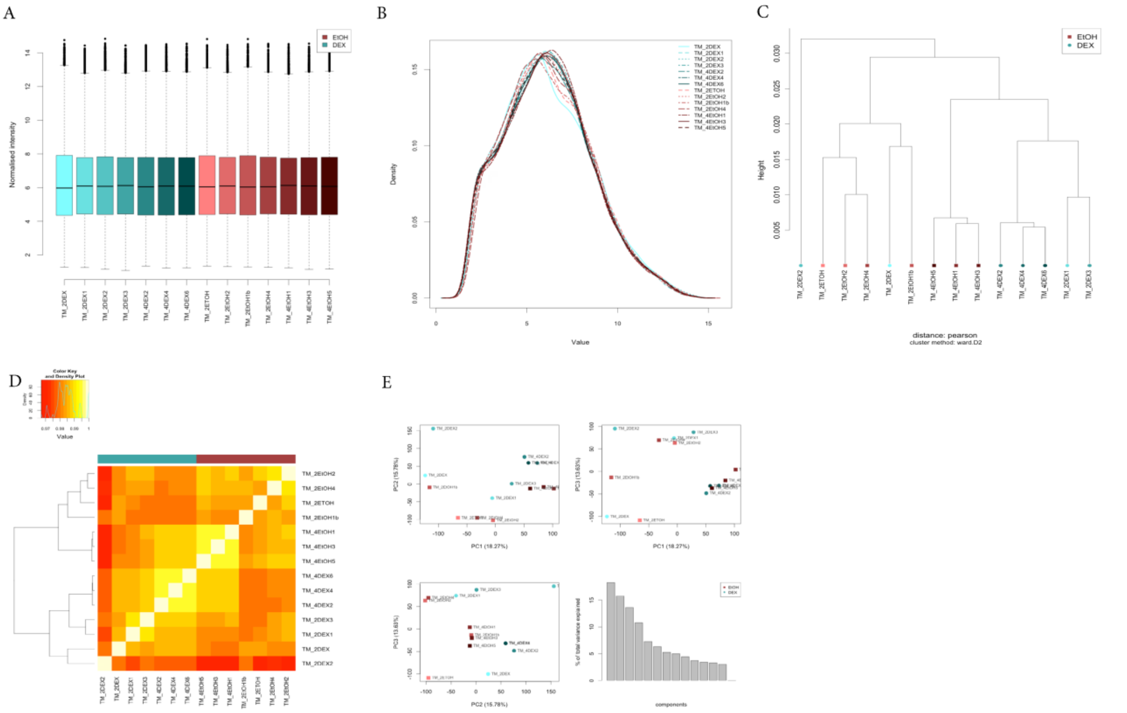

Supplemental figure 5.3 Quality control of dataset GSE124114: normalised data, outliers removed
A. Boxplot of the signal intensities; B. Density histogram of the signal intensities; C. Cluster dendrogram; D. Correlation plot; E. PCA-analysis
