# Supplementary material for: The Molecular Processes in the Trabecular Meshwork After Exposure to Corticosteroids and in Corticosteroid-Induced Ocular Hypertension
Source: Invest Ophthalmol Vis Sci. 2020 Apr 18;61(4):24. doi: 10.1167/iovs.61.4.24 (PMC7401422; doi:10.1167/iovs.61.4.24)
Supplement: Supplement 2 [file iovs-61-4-24_s002.pdf]

**Appendix 2. Complete pathway results of effect of dexamethasone on TM: combined human datasets**

| Pathway | Z Score | Permuted p-value | Positive* | Measured* |
|---|---|---|---|---|
| Reactome: Interleukin-4 and Interleukin-13 signaling | 8.27 | <0.0001 | 68 | 200 |
| Reactome: Metallothioneins bind metals | 6.52 | <0.0001 | 9 | 11 |
| Reactome: Class A/1 (Rhodopsin-like receptors) | 6.05 | <0.0001 | 79 | 307 |
| Reactome: Regulation of Insulin-like Growth Factor (IGF) transport and uptake by Insulin-like Growth Factor Binding Proteins (IGFBPs) | 5.8 | <0.0001 | 39 | 122 |
| KEGG: Complement and coagulation cascades | 5.55 | <0.0001 | 27 | 75 |
| WP: Prostaglandin Synthesis and Regulation | 5.51 | <0.0001 | 19 | 45 |
| WP: GPCRs, Class A Rhodopsin-like | 5.5 | <0.0001 | 63 | 242 |
| Reactome: Extracellular matrix organization | 5.23 | <0.0001 | 27 | 79 |
| WP: Vitamin D Receptor Pathway | 5.12 | <0.0001 | 47 | 172 |
| Reactome: Integrin cell surface interactions | 5.11 | <0.0001 | 23 | 64 |
| Reactome: Post-translational protein phosphorylation | 4.93 | <0.0001 | 32 | 105 |
| WP: Differentiation Pathway | 4.63 | <0.0001 | 18 | 49 |
| WP: Photodynamic therapy-induced NF-kB survival signaling | 4.61 | <0.0001 | 14 | 34 |
| WP: Macrophage markers | 4.58 | <0.0001 | 6 | 9 |
| Reactome: Interleukin-10 signaling | 4.58 | <0.0001 | 25 | 79 |
| KEGG: Neuroactive ligand-receptor interaction | 4.54 | <0.0001 | 64 | 275 |
| WP: Nuclear Receptors Meta-Pathway | 4.53 | <0.0001 | 70 | 308 |
| WP: Spinal Cord Injury | 4.45 | <0.0001 | 32 | 113 |
| Reactome: G alpha (q) signalling events | 4.41 | <0.0001 | 41 | 158 |
| KEGG: Cytokine-cytokine receptor interaction | 4.36 | <0.0001 | 63 | 275 |
| Reactome: Interleukin-7 signaling | 4.29 | <0.0001 | 12 | 29 |
| WP: Endochondral Ossification | 4.11 | <0.0001 | 20 | 63 |
| Reactome: Bile acid and bile salt metabolism | 4.1 | <0.0001 | 15 | 42 |
| Reactome: Interferon alpha/beta signaling | 4.03 | <0.0001 | 22 | 73 |
| KEGG: Hematopoietic cell lineage | 4.02 | <0.0001 | 25 | 87 |
| WP: Amplification and Expansion of Oncogenic Pathways as Metastatic Traits | 3.96 | <0.0001 | 8 | 17 |
| WP: Adipogenesis | 3.91 | <0.0001 | 33 | 128 |
| WP: GPCRs, Other | 3.9 | <0.0001 | 24 | 84 |
| WP: Monoamine GPCRs | 3.74 | 0.002 | 12 | 33 |
| WP: Ectoderm Differentiation | 3.67 | 0.001 | 34 | 138 |
| Reactome: Regulation of lipid metabolism by Peroxisome proliferator-activated receptor alpha (PPARalph | 3.66 | <0.0001 | 37 | 154 |
| Reactome: Phase I - Functionalization of compounds | 3.63 | <0.0001 | 26 | 98 |
| WP: Benzo(a)pyrene metabolism | 3.62 | 0.003 | 5 | 9 |
| Reactome: L1CAM interactions | 3.57 | 0.001 | 25 | 94 |
| WP: Complement and Coagulation Cascades | 3.57 | <0.0001 | 17 | 56 |
| WP: Nicotine Activity on Chromaffin Cells | 3.54 | 0.002 | 3 | 4 |
| WP: Proprotein convertase subtilisin/kexin type 9 (PCSK9) mediated LDL receptor degradation | 3.52 | <0.0001 | 2 | 2 |
| WP: Evolocumab Mechanism | 3.52 | 0.002 | 2 | 2 |
| Reactome: Formation of Fibrin Clot (Clotting Cascade) | 3.52 | 0.001 | 13 | 39 |
| WP: Development and heterogeneity of the ILC family | 3.48 | 0.002 | 11 | 31 |
| Reactome: Cell surface interactions at the vascular wall | 3.44 | <0.0001 | 31 | 127 |
| Reactome: Binding and Uptake of Ligands by Scavenger Receptors | 3.41 | <0.0001 | 13 | 40 |
| KEGG: ECM-receptor interaction | 3.4 | 0.004 | 22 | 82 |
| WP: Vitamin B12 Metabolism | 3.39 | <0.0001 | 15 | 49 |
| KEGG: Tyrosine metabolism | 3.38 | 0.001 | 12 | 36 |
| Reactome: Dissolution of Fibrin Clot | 3.37 | 0.002 | 6 | 13 |
| WP: Genes targeted by miRNAs in adipocytes | 3.37 | 0.009 | 6 | 13 |
| KEGG: Steroid hormone biosynthesis | 3.35 | 0.001 | 14 | 45 |
| WP: Arrhythmogenic Right Ventricular Cardiomyopathy | 3.28 | 0.001 | 20 | 74 |
| WP: Zinc homeostasis | 3.27 | 0.001 | 12 | 37 |
| KEGG: Nitrogen metabolism | 3.26 | 0.001 | 7 | 17 |
| WP: Epithelial to mesenchymal transition in colorectal cancer | 3.25 | <0.0001 | 36 | 158 |
| WP: Matrix Metalloproteinases | 3.21 | 0.002 | 10 | 29 |
| WP: IL-7 Signaling Pathway | 3.2 | 0.002 | 9 | 25 |
| WP: Thymic Stromal LymphoPoietin (TSLP) Signaling Pathway | 3.16 | 0.002 | 14 | 47 |
| WP: Sulfation Biotransformation Reaction | 3.14 | 0.004 | 6 | 14 |
| WP: Overview of nanoparticle effects | 3.07 | 0.004 | 7 | 18 |
| WP: Copper homeostasis | 3.04 | 0.003 | 15 | 53 |
| WP: Serotonin Transporter Activity | 3.03 | 0.005 | 5 | 11 |
| WP: Catalytic cycle of mammalian Flavin-containing MonoOxygenases (FMOs) | 2.98 | 0.01 | 3 | 5 |
| WP: Caloric restriction and aging | 2.96 | 0.016 | 4 | 8 |
| Reactome: Collagen degradation | 2.95 | 0.003 | 12 | 40 |
| WP: Small Ligand GPCRs | 2.9 | 0.005 | 7 | 19 |
| WP: Farnesoid X Receptor Pathway | 2.9 | 0.008 | 7 | 19 |
| WP: Extracellular vesicles in the crosstalk of cardiac cells | 2.9 | 0.011 | 7 | 19 |
| WP: Platelet-mediated interactions with vascular and circulating cells | 2.9 | 0.014 | 7 | 19 |
| WP: Hypertrophy Model | 2.9 | 0.006 | 7 | 19 |
| WP: Complement Activation | 2.9 | 0.01 | 7 | 19 |
| WP: Oncostatin M Signaling Pathway | 2.87 | 0.004 | 17 | 65 |

| | | | | |
|---|---|---|---|---|
| WP: Metapathway biotransformation Phase I and II | 2.84 | 0.003 | 36 | 168 |
| WP: IL1 and megakaryocytes in obesity | 2.76 | 0.009 | 8 | 24 |
| Reactome: Glycosaminoglycan metabolism | 2.74 | 0.009 | 27 | 120 |
| WP: Osteoclast Signaling | 2.73 | 0.008 | 6 | 16 |
| KEGG: Glycosaminoglycan biosynthesis | 2.73 | 0.007 | 7 | 20 |
| WP: Imatinib and Chronic Myeloid Leukemia | 2.73 | 0.008 | 7 | 20 |
| KEGG: Starch and sucrose metabolism | 2.73 | 0.013 | 10 | 33 |
| Reactome: G alpha (i) signalling events | 2.73 | 0.006 | 49 | 247 |
| WP: ncRNAs involved in Wnt signaling in hepatocellular carcinoma | 2.7 | 0.011 | 20 | 83 |
| KEGG: JAK-STAT signaling pathway | 2.68 | 0.011 | 33 | 155 |
| WP: Human Complement System | 2.65 | 0.011 | 23 | 100 |
| WP: Metabolism of Tetrahydrocannabinol (THC) | 2.64 | 0.01 | 2 | 3 |
| Reactome: Incretin synthesis, secretion, and inactivation | 2.62 | 0.015 | 8 | 25 |
| WP: Nicotine Activity on Dopaminergic Neurons | 2.58 | 0.009 | 7 | 21 |
| WP: Transcription factor regulation in adipogenesis | 2.58 | 0.014 | 7 | 21 |
| WP: Hepatitis C and Hepatocellular Carcinoma | 2.57 | 0.008 | 13 | 49 |
| Reactome: Signaling by Activin | 2.56 | 0.005 | 5 | 13 |
| WP: EV release from cardiac cells and their functional effects | 2.56 | 0.018 | 3 | 6 |
| WP: Robo4 and VEGF Signaling Pathways Crosstalk | 2.56 | 0.019 | 3 | 6 |
| KEGG: Phenylalanine metabolism | 2.56 | 0.007 | 6 | 17 |
| WP: Fibrin Complement Receptor 3 Signaling Pathway | 2.52 | 0.01 | 10 | 35 |
| WP: Selenium Micronutrient Network | 2.5 | 0.013 | 19 | 81 |
| KEGG: Tryptophan metabolism | 2.49 | 0.012 | 11 | 40 |
| Reactome: Latent infection of Homo sapiens with Mycobacterium tuberculosis | 2.49 | 0.011 | 1 | 1 |
| Reactome: Assembly of collagen fibrils and other multimeric structures | 2.48 | 0.015 | 12 | 45 |
| KEGG: Mineral absorption | 2.48 | 0.014 | 13 | 50 |
| KEGG: Cell adhesion molecules (CAMs) | 2.45 | 0.011 | 28 | 132 |
| WP: Monoamine Transport | 2.44 | 0.02 | 9 | 31 |
| WP: Eicosanoid Synthesis | 2.44 | 0.019 | 7 | 22 |
| WP: miRNA targets in ECM and membrane receptors | 2.44 | 0.012 | 7 | 22 |
| WP: PI3K/AKT/mTOR - VitD3 Signalling | 2.44 | 0.013 | 7 | 22 |
| Reactome: Elastic fibre formation | 2.4 | 0.016 | 11 | 41 |
| Reactome: Collagen chain trimerization | 2.4 | 0.031 | 11 | 41 |
| WP: Aryl Hydrocarbon Receptor | 2.4 | 0.016 | 12 | 46 |
| Reactome: Abacavir transport and metabolism | 2.39 | 0.015 | 4 | 10 |
| Reactome: POU5F1 (OCT4), SOX2, NANOG repress genes related to differentiation | 2.39 | 0.015 | 4 | 10 |
| WP: TGF-B Signaling in Thyroid Cells for Epithelial-Mesenchymal Transition | 2.39 | 0.026 | 6 | 18 |
| WP: Oligodendrocyte Specification and differentiation(including remyelination), leading to Myelin Components for CNS | 2.37 | 0.011 | 8 | 27 |
| Reactome: Acetylcholine binding and downstream events | 2.36 | 0.019 | 5 | 14 |
| Reactome: Complement cascade | 2.32 | 0.016 | 13 | 52 |
| WP: Photodynamic therapy-induced HIF-1 survival signaling | 2.32 | 0.016 | 10 | 37 |
| WP: GPCRs, Class B Secretin-like | 2.3 | 0.026 | 7 | 23 |
| KEGG: Osteoclast differentiation | 2.29 | 0.014 | 26 | 124 |
| WP: PI3K-Akt Signaling Pathway | 2.28 | 0.027 | 60 | 330 |
| Reactome: TAK1 activates NFkB by phosphorylation and activation of IKKs complex | 2.25 | 0.014 | 8 | 28 |
| WP: Gastric Cancer Network 1 | 2.25 | 0.019 | 8 | 28 |
| KEGG: PI3K-Akt signaling pathway | 2.24 | 0.022 | 62 | 344 |
| Reactome: Activation of Matrix Metalloproteinases | 2.23 | 0.019 | 9 | 33 |
| Reactome: Aryl hydrocarbon receptor signalling | 2.22 | 0.023 | 3 | 7 |
| WP: Gastric acid production | 2.22 | 0.026 | 3 | 7 |
| WP: Mevalonate pathway | 2.22 | 0.025 | 3 | 7 |
| KEGG: Renin secretion | 2.22 | 0.026 | 15 | 64 |
| Reactome: Post-translational modification: synthesis of GPI-anchored proteins | 2.21 | 0.027 | 19 | 86 |
| WP: Lung fibrosis | 2.19 | 0.016 | 14 | 59 |
| WP: Brain-Derived Neurotrophic Factor (BDNF) signaling pathway | 2.18 | 0.024 | 29 | 144 |
| Reactome: Signaling by Type 1 Insulin-like Growth Factor 1 Receptor (IGF1R) | 2.18 | 0.027 | 5 | 15 |
| WP: Fatty Acid Omega Oxidation | 2.18 | 0.027 | 5 | 15 |
| WP: Nucleotide GPCRs | 2.16 | 0.034 | 4 | 11 |
| WP: Oxidative Stress | 2.14 | 0.032 | 8 | 29 |
| WP: Statin Pathway | 2.14 | 0.023 | 8 | 29 |
| KEGG: Calcium signaling pathway | 2.13 | 0.027 | 35 | 181 |
| WP: LncRNA involvement in canonical Wnt signaling and colorectal cancer | 2.13 | 0.028 | 20 | 93 |
| WP: Microglia Pathogen Phagocytosis Pathway | 2.13 | 0.027 | 10 | 39 |
| KEGG: Arachidonic acid metabolism | 2.12 | 0.032 | 14 | 60 |
| WP: RANKL/RANK (Receptor activator of NFKB (ligand)) Signaling Pathway | 2.1 | 0.024 | 13 | 55 |
| Reactome: Neurotransmitter uptake and metabolism In glial cells | 2.09 | 0.041 | 2 | 4 |
| WP: Nicotine Metabolism | 2.09 | 0.036 | 2 | 4 |
| WP: Neurotransmitter Disorders | 2.09 | 0.03 | 2 | 4 |
| KEGG: Glycolysis / Gluconeogenesis | 2.08 | 0.041 | 15 | 66 |
| WP: Primary Focal Segmental Glomerulosclerosis FSGS | 2.05 | 0.03 | 16 | 72 |
| KEGG: Circadian rhythm | 2.03 | 0.03 | 8 | 30 |
| Reactome: Interleukin-2 family signaling | 2.03 | 0.044 | 8 | 30 |
| Reactome: Collagen biosynthesis and modifying enzymes | 2.02 | 0.048 | 15 | 67 |
| WP: SREBF and miR33 in cholesterol and lipid homeostasis | 2.01 | 0.042 | 5 | 16 |

| | | | | |
|---|---|---|---|---|
| KEGG: TNF signaling pathway | 2 | 0.033 | 22 | 107 |
| WP: Focal Adhesion | 2 | 0.04 | 37 | 197 |
| Reactome: TP53 Regulates Transcription of Cell Cycle Genes | 1.99 | 0.04 | 14 | 62 |
| WP: Tryptophan metabolism | 1.97 | 0.058 | 11 | 46 |
| Reactome: Cell junction organization | 1.95 | 0.058 | 18 | 85 |
| Reactome: alpha-linolenic (omega3) and linoleic (omega6) acid metabolism | 1.95 | 0.054 | 4 | 12 |
| Reactome: Reversible hydration of carbon dioxide | 1.95 | 0.033 | 4 | 12 |
| KEGG: Riboflavin metabolism | 1.93 | 0.041 | 3 | 8 |
| KEGG: Retinol metabolism | 1.92 | 0.054 | 12 | 52 |
| KEGG: Olfactory transduction | 1.91 | 0.066 | 31 | 163 |
| Reactome: Signaling by Retinoic Acid | 1.86 | 0.066 | 10 | 42 |
| WP: Splicing factor NOVA regulated synaptic proteins | 1.86 | 0.036 | 10 | 42 |
| WP: Vitamin A and Carotenoid Metabolism | 1.86 | 0.052 | 10 | 42 |
| KEGG: Leukocyte transendothelial migration | 1.86 | 0.064 | 22 | 110 |
| KEGG: Primary bile acid biosynthesis | 1.85 | 0.06 | 5 | 17 |
| WP: NOTCH1 regulation of human endothelial cell calcification | 1.85 | 0.049 | 5 | 17 |
| WP: Simplified Interaction Map Between LOXL4 and Oxidative Stress Pathway | 1.85 | 0.052 | 5 | 17 |
| WP: ACE Inhibitor Pathway | 1.85 | 0.052 | 5 | 17 |
| WP: Focal Adhesion-PI3K-Akt-mTOR-signaling pathway | 1.82 | 0.061 | 52 | 297 |
| Reactome: RAF-independent MAPK1/3 activation | 1.82 | 0.06 | 6 | 22 |
| Reactome: Amino acid synthesis and interconversion (transamination) | 1.82 | 0.077 | 6 | 22 |
| KEGG: Drug metabolism | 1.81 | 0.066 | 13 | 59 |
| Reactome: Arachidonic acid metabolism | 1.81 | 0.064 | 13 | 59 |
| WP: Sudden Infant Death Syndrome (SIDS) Susceptibility Pathways | 1.79 | 0.069 | 30 | 160 |
| WP: Folate Metabolism | 1.79 | 0.078 | 14 | 65 |
| WP: VEGFA-VEGFR2 Signaling Pathway | 1.78 | 0.067 | 42 | 235 |
| KEGG: Th17 cell differentiation | 1.78 | 0.072 | 20 | 100 |
| WP: Cell-type Dependent Selectivity of CCK2R Signaling | 1.76 | 0.061 | 4 | 13 |
| Reactome: RET signaling | 1.75 | 0.071 | 9 | 38 |
| KEGG: Focal adhesion | 1.73 | 0.075 | 36 | 199 |
| WP: Wnt Signaling Pathway and Pluripotency | 1.73 | 0.089 | 20 | 101 |
| Reactome: Circadian Clock | 1.73 | 0.082 | 14 | 66 |
| KEGG: Rap1 signaling pathway | 1.7 | 0.112 | 37 | 206 |
| KEGG: Histidine metabolism | 1.69 | 0.084 | 6 | 23 |
| Reactome: Interleukin-6 family signaling | 1.69 | 0.079 | 6 | 23 |
| KEGG: Neomycin, kanamycin and gentamicin biosynthesis | 1.69 | 0.085 | 2 | 5 |
| KEGG: Vitamin B6 metabolism | 1.69 | 0.063 | 2 | 5 |
| Reactome: Melanin biosynthesis | 1.69 | 0.07 | 2 | 5 |
| Reactome: tRNA processing in the mitochondrion | 1.69 | 0.097 | 2 | 5 |
| WP: SCFA and skeletal muscle substrate metabolism | 1.69 | 0.047 | 2 | 5 |
| WP: Methylation Pathways | 1.69 | 0.069 | 3 | 9 |
| Reactome: O-linked glycosylation | 1.68 | 0.085 | 21 | 108 |
| KEGG: Glycine, serine and threonine metabolism | 1.66 | 0.085 | 9 | 39 |
| KEGG: Cholesterol metabolism | 1.66 | 0.088 | 11 | 50 |
| WP: Hematopoietic Stem Cell Differentiation | 1.64 | 0.097 | 12 | 56 |
| WP: Wnt Signaling Pathway | 1.63 | 0.096 | 22 | 115 |
| WP: Oxidation by Cytochrome P450 | 1.62 | 0.094 | 13 | 62 |
| Reactome: Gap junction trafficking and regulation | 1.6 | 0.094 | 7 | 29 |
| WP: Pregnane X Receptor pathway | 1.6 | 0.081 | 7 | 29 |
| WP: Osteoblast Signaling | 1.59 | 0.08 | 4 | 14 |
| KEGG: Toll-like receptor signaling pathway | 1.58 | 0.109 | 18 | 92 |
| KEGG: Gastric cancer | 1.58 | 0.109 | 27 | 147 |
| WP: Bladder Cancer | 1.58 | 0.108 | 9 | 40 |
| KEGG: Glycosaminoglycan biosynthesis | 1.58 | 0.085 | 6 | 24 |
| Reactome: Cholesterol biosynthesis | 1.58 | 0.119 | 6 | 24 |
| WP: Angiogenesis | 1.58 | 0.073 | 6 | 24 |
| KEGG: Steroid biosynthesis | 1.57 | 0.112 | 5 | 19 |
| WP: Tamoxifen metabolism | 1.57 | 0.103 | 5 | 19 |
| KEGG: Alanine, aspartate and glutamate metabolism | 1.54 | 0.132 | 8 | 35 |
| WP: Melatonin metabolism and effects | 1.54 | 0.104 | 8 | 35 |
| WP: miRNAs involvement in the immune response in sepsis | 1.54 | 0.118 | 8 | 35 |
| Reactome: Degradation of the extracellular matrix | 1.54 | 0.115 | 15 | 75 |
| WP: Peptide GPCRs | 1.54 | 0.121 | 15 | 75 |
| WP: Apoptosis-related network due to altered Notch3 in ovarian cancer | 1.52 | 0.118 | 11 | 52 |
| WP: Breast cancer pathway | 1.51 | 0.136 | 28 | 155 |
| KEGG: AMPK signaling pathway | 1.5 | 0.121 | 22 | 118 |
| KEGG: Mucin type O-glycan biosynthesis | 1.5 | 0.109 | 7 | 30 |
| WP: Dopaminergic Neurogenesis | 1.5 | 0.111 | 7 | 30 |
| WP: Inflammatory Response Pathway | 1.5 | 0.115 | 7 | 30 |
| KEGG: Serotonergic synapse | 1.5 | 0.125 | 21 | 112 |
| Reactome: Biosynthesis of electrophilic Ï‰-3 PUFA oxo-derivatives | 1.48 | 0.133 | 1 | 2 |
| WP: Hypoxia-mediated EMT and Stemness | 1.48 | 0.065 | 1 | 2 |
| WP: Mevalonate arm of cholesterol biosynthesis pathway with inhibitors | 1.48 | 0.145 | 1 | 2 |
| KEGG: Synthesis and degradation of ketone bodies | 1.48 | 0.095 | 3 | 10 |
| Reactome: Neurotoxicity of clostridium toxins | 1.48 | 0.139 | 3 | 10 |

| | | | | |
|---|---|---|---|---|
| KEGG: MAPK signaling pathway | 1.47 | 0.146 | 49 | 291 |
| WP: PPAR Alpha Pathway | 1.46 | 0.103 | 6 | 25 |
| WP: Cytokines and Inflammatory Response | 1.46 | 0.12 | 6 | 25 |
| WP: Cardiac Progenitor Differentiation | 1.45 | 0.128 | 11 | 53 |
| Reactome: Syndecan interactions | 1.44 | 0.138 | 5 | 20 |
| Reactome: Cellular hexose transport | 1.44 | 0.127 | 5 | 20 |
| Reactome: Signaling by NTRK3 (TRKC) | 1.43 | 0.151 | 4 | 15 |
| WP: Cholesterol Biosynthesis | 1.43 | 0.117 | 4 | 15 |
| Reactome: Class B/2 (Secretin family receptors) | 1.43 | 0.152 | 18 | 95 |
| KEGG: Bile secretion | 1.43 | 0.161 | 14 | 71 |
| Reactome: Nicotinate metabolism | 1.4 | 0.146 | 7 | 31 |
| WP: Factors and pathways affecting insulin-like growth factor (IGF1)-Akt signaling | 1.4 | 0.169 | 7 | 31 |
| KEGG: Regulation of lipolysis in adipocytes | 1.38 | 0.162 | 11 | 54 |
| Reactome: Galactose catabolism | 1.38 | 0.094 | 2 | 6 |
| Reactome: Synthesis of Lipoxins (LX) | 1.38 | 0.181 | 2 | 6 |
| WP: Thyroxine (Thyroid Hormone) Production | 1.38 | 0.096 | 2 | 6 |
| WP: Metastatic brain tumor | 1.38 | 0.148 | 2 | 6 |
| WP: let-7 inhibition of ES cell reprogramming | 1.38 | 0.092 | 2 | 6 |
| WP: Somatroph axis (GH) and its relationship to dietary restriction and aging | 1.38 | 0.089 | 2 | 6 |
| Reactome: Potassium Channels | 1.37 | 0.169 | 12 | 60 |
| Reactome: BMAL1:CLOCK,NPAS2 activates circadian gene expression | 1.36 | 0.143 | 8 | 37 |
| KEGG: Biosynthesis of unsaturated fatty acids | 1.36 | 0.165 | 6 | 26 |
| Reactome: Toll-like Receptor Cascades | 1.36 | 0.155 | 6 | 26 |
| WP: Wnt/beta-catenin Signaling Pathway in Leukemia | 1.36 | 0.167 | 6 | 26 |
| Reactome: Transcriptional regulation of white adipocyte differentiation | 1.34 | 0.17 | 18 | 97 |
| WP: Circadian rythm related genes | 1.33 | 0.165 | 35 | 205 |
| Reactome: Regulation of TLR by endogenous ligand | 1.29 | 0.188 | 4 | 16 |
| WP: Tryptophan catabolism leading to NAD+ production | 1.29 | 0.183 | 4 | 16 |
| WP: Glucuronidation | 1.29 | 0.212 | 4 | 16 |
| WP: Vitamin D Metabolism | 1.29 | 0.145 | 3 | 11 |
| Reactome: Neurotransmitter release cycle | 1.28 | 0.209 | 8 | 38 |
| WP: Toll-like Receptor Signaling Pathway | 1.28 | 0.203 | 17 | 92 |
| Reactome: Interferon gamma signaling | 1.27 | 0.192 | 16 | 86 |
| WP: Hair Follicle Development: Cytodifferentiation (Part 3 of 3) | 1.27 | 0.194 | 16 | 86 |
| KEGG: Ether lipid metabolism | 1.26 | 0.211 | 9 | 44 |
| WP: Heart Development | 1.26 | 0.228 | 9 | 44 |
| KEGG: Longevity regulating pathway | 1.25 | 0.234 | 12 | 62 |
| KEGG: Signaling pathways regulating pluripotency of stem cells | 1.24 | 0.213 | 24 | 137 |
| Reactome: Ion channel transport | 1.22 | 0.206 | 30 | 176 |
| KEGG: Salivary secretion | 1.22 | 0.217 | 16 | 87 |
| WP: Alpha 6 Beta 4 signaling pathway | 1.22 | 0.241 | 7 | 33 |
| WP: Nuclear Receptors in Lipid Metabolism and Toxicity | 1.22 | 0.204 | 7 | 33 |
| KEGG: alpha-Linolenic acid metabolism | 1.2 | 0.235 | 5 | 22 |
| Reactome: Interleukin-12 family signaling | 1.2 | 0.23 | 5 | 22 |
| WP: Photodynamic therapy-induced NFE2L2 (NRF2) survival signaling | 1.2 | 0.249 | 5 | 22 |
| WP: Regulation of Apoptosis by Parathyroid Hormone-related Protein | 1.2 | 0.24 | 5 | 22 |
| WP: Synaptic Vesicle Pathway | 1.19 | 0.246 | 10 | 51 |
| WP: Regulation of toll-like receptor signaling pathway | 1.18 | 0.25 | 23 | 132 |
| Reactome: Metabolism of Angiotensinogen to Angiotensins | 1.15 | 0.254 | 4 | 17 |
| WP: Regulation of Wnt/B-catenin Signaling by Small Molecule Compounds | 1.15 | 0.236 | 4 | 17 |
| WP: Leptin Insulin Overlap | 1.15 | 0.175 | 4 | 17 |
| WP: Serotonin and anxiety | 1.15 | 0.218 | 4 | 17 |
| KEGG: Prolactin signaling pathway | 1.14 | 0.262 | 13 | 70 |
| WP: Non-genomic actions of 1,25 dihydroxyvitamin D3 | 1.14 | 0.243 | 13 | 70 |
| WP: Type II interferon signaling (IFNG) | 1.13 | 0.263 | 7 | 34 |
| WP: AMP-activated Protein Kinase (AMPK) Signaling | 1.13 | 0.254 | 12 | 64 |
| Reactome: Interleukin-1 processing | 1.12 | 0.25 | 2 | 7 |
| WP: Glial Cell Differentiation | 1.12 | 0.119 | 2 | 7 |
| WP: Glucocorticoid and Mineralcorticoid Metabolism | 1.12 | 0.301 | 2 | 7 |
| WP: MicroRNA for Targeting Cancer Growth and Vascularization in Glioblastoma | 1.12 | 0.119 | 2 | 7 |
| Reactome: Phase II - Conjugation of compounds | 1.12 | 0.264 | 16 | 89 |
| KEGG: Ovarian steroidogenesis | 1.12 | 0.274 | 9 | 46 |
| Reactome: Rap1 signalling | 1.11 | 0.267 | 3 | 12 |
| Reactome: Import of palmitoyl-CoA into the mitochondrial matrix | 1.11 | 0.268 | 3 | 12 |
| WP: Gene regulatory network modelling somitogenesis | 1.11 | 0.169 | 3 | 12 |
| KEGG: cGMP-PKG signaling pathway | 1.1 | 0.281 | 27 | 160 |
| Reactome: Response to elevated platelet cytosolic Ca2+ | 1.09 | 0.264 | 20 | 115 |
| KEGG: Proximal tubule bicarbonate reclamation | 1.09 | 0.294 | 5 | 23 |
| Reactome: Growth hormone receptor signaling | 1.09 | 0.279 | 5 | 23 |
| WP: The human immune response to tuberculosis | 1.09 | 0.322 | 5 | 23 |
| KEGG: Regulation of actin cytoskeleton | 1.08 | 0.291 | 35 | 213 |
| WP: Myometrial Relaxation and Contraction Pathways | 1.08 | 0.303 | 26 | 154 |
| KEGG: IL-17 signaling pathway | 1.07 | 0.307 | 16 | 90 |
| KEGG: Nicotinate and nicotinamide metabolism | 1.06 | 0.295 | 6 | 29 |
| WP: Allograft Rejection | 1.06 | 0.257 | 15 | 84 |

| | | | | |
|---|---|---|---|---|
| WP: TGF-beta Receptor Signaling | 1.05 | 0.308 | 10 | 53 |
| Reactome: Detoxification of Reactive Oxygen Species | 1.05 | 0.3 | 7 | 35 |
| WP: NRF2 pathway | 1.03 | 0.337 | 23 | 136 |
| Reactome: GABA synthesis, release, reuptake and degradation | 1.02 | 0.303 | 4 | 18 |
| Reactome: Amine-derived hormones | 1.02 | 0.299 | 4 | 18 |
| WP: AGE/RAGE pathway | 1.01 | 0.307 | 12 | 66 |
| WP: IL-4 Signaling Pathway | 0.99 | 0.337 | 10 | 54 |
| Reactome: Insulin-like Growth Factor-2 mRNA Binding Proteins (IGF2BPs/IMPs/VICKZs) bind RNA | 0.97 | 0.402 | 1 | 3 |
| WP: Vitamins A and D - action mechanisms | 0.97 | 0.323 | 1 | 3 |
| WP: Chromosomal and microsatellite instability in colorectal cancer | 0.97 | 0.343 | 13 | 73 |
| WP: Extracellular vesicle-mediated signaling in recipient cells | 0.97 | 0.346 | 6 | 30 |
| WP: IL-2 Signaling Pathway | 0.97 | 0.312 | 8 | 42 |
| WP: Target Of Rapamycin (TOR) Signaling | 0.97 | 0.302 | 7 | 36 |
| WP: Wnt Signaling in Kidney Disease | 0.97 | 0.329 | 7 | 36 |
| Reactome: Advanced glycosylation endproduct receptor signaling | 0.96 | 0.353 | 3 | 13 |
| Reactome: Passive transport by Aquaporins | 0.96 | 0.292 | 3 | 13 |
| Reactome: Platelet Adhesion to exposed collagen | 0.96 | 0.34 | 3 | 13 |
| Reactome: RHO GTPases Activate NADPH Oxidases | 0.96 | 0.31 | 3 | 13 |
| WP: Osteopontin Signaling | 0.96 | 0.349 | 3 | 13 |
| WP: Dopamine metabolism | 0.96 | 0.324 | 3 | 13 |
| WP: Estrogen Receptor Pathway | 0.96 | 0.374 | 3 | 13 |
| WP: ncRNAs involved in STAT3 signaling in hepatocellular carcinoma | 0.96 | 0.424 | 3 | 13 |
| KEGG: Cushing syndrome | 0.96 | 0.34 | 25 | 151 |
| KEGG: Glycerophospholipid metabolism | 0.93 | 0.358 | 16 | 93 |
| Reactome: Erythrocytes take up oxygen and release carbon dioxide | 0.91 | 0.368 | 2 | 8 |
| Reactome: tRNA modification in the mitochondrion | 0.91 | 0.35 | 2 | 8 |
| WP: NAD Biosynthesis II (from tryptophan) | 0.91 | 0.363 | 2 | 8 |
| WP: FTO Obesity Variant Mechanism | 0.91 | 0.28 | 2 | 8 |
| WP: Insulin signalling in human adipocytes (normal condition) | 0.91 | 0.377 | 2 | 8 |
| WP: Insulin signalling in human adipocytes (diabetic condition) | 0.91 | 0.318 | 2 | 8 |
| WP: ApoE and miR-146 in inflammation and atherosclerosis | 0.91 | 0.265 | 2 | 8 |
| KEGG: Fc gamma R-mediated phagocytosis | 0.91 | 0.363 | 15 | 87 |
| Reactome: Signaling by NODAL | 0.91 | 0.327 | 4 | 19 |
| Reactome: SALM protein interactions at the synapse | 0.91 | 0.365 | 4 | 19 |
| WP: Glutathione metabolism | 0.91 | 0.364 | 4 | 19 |
| KEGG: Adipocytokine signaling pathway | 0.9 | 0.333 | 12 | 68 |
| WP: Glycogen Metabolism | 0.9 | 0.394 | 8 | 43 |
| KEGG: Aldosterone-regulated sodium reabsorption | 0.89 | 0.367 | 7 | 37 |
| Reactome: Transport of vitamins, nucleosides, and related molecules | 0.89 | 0.364 | 7 | 37 |
| WP: Differentiation of white and brown adipocyte | 0.89 | 0.428 | 5 | 25 |
| KEGG: Hippo signaling pathway | 0.88 | 0.375 | 25 | 153 |
| KEGG: Galactose metabolism | 0.88 | 0.397 | 6 | 31 |
| Reactome: DAP12 interactions | 0.88 | 0.387 | 6 | 31 |
| Reactome: MyD88 dependent cascade initiated on endosome | 0.88 | 0.384 | 6 | 31 |
| WP: Ethanol effects on histone modifications | 0.88 | 0.417 | 6 | 31 |
| WP: Neural Crest Differentiation | 0.86 | 0.365 | 17 | 101 |
| KEGG: Estrogen signaling pathway | 0.85 | 0.421 | 22 | 134 |
| WP: Structural Pathway of Interleukin 1 (IL-1) | 0.84 | 0.388 | 9 | 50 |
| WP: One carbon metabolism and related pathways | 0.84 | 0.418 | 9 | 50 |
| WP: Sleep regulation | 0.81 | 0.419 | 7 | 38 |
| Reactome: Retinoid metabolism and transport | 0.8 | 0.419 | 6 | 32 |
| WP: White fat cell differentiation | 0.8 | 0.414 | 6 | 32 |
| KEGG: FoxO signaling pathway | 0.79 | 0.45 | 21 | 129 |
| WP: Calcium Regulation in the Cardiac Cell | 0.79 | 0.432 | 24 | 149 |
| KEGG: Linoleic acid metabolism | 0.79 | 0.447 | 5 | 26 |
| Reactome: Interconversion of nucleotide di- and triphosphates | 0.79 | 0.415 | 5 | 26 |
| Reactome: Glycogen metabolism | 0.79 | 0.433 | 5 | 26 |
| Reactome: Integrin alphaIIb beta3 signaling | 0.79 | 0.453 | 5 | 26 |
| WP: ESC Pluripotency Pathways | 0.78 | 0.437 | 19 | 116 |
| Reactome: Immunoregulatory interactions between a Lymphoid and a non-Lymphoid cell | 0.75 | 0.45 | 16 | 97 |
| Reactome: Cardiac conduction | 0.74 | 0.479 | 22 | 137 |
| Reactome: Signaling by SCF-KIT | 0.74 | 0.443 | 7 | 39 |
| WP: Mesodermal Commitment Pathway | 0.73 | 0.487 | 23 | 144 |
| KEGG: Sulfur metabolism | 0.72 | 0.482 | 2 | 9 |
| Reactome: Signaling by Leptin | 0.72 | 0.46 | 2 | 9 |
| Reactome: Interleukin-9 signaling | 0.72 | 0.499 | 2 | 9 |
| Reactome: OAS antiviral response | 0.72 | 0.461 | 2 | 9 |
| WP: Folate-Alcohol and Cancer Pathway Hypotheses | 0.72 | 0.468 | 2 | 9 |
| WP: Metabolism of Spingolipids in ER and Golgi apparatus | 0.72 | 0.465 | 2 | 9 |
| Reactome: Signaling by PDGF | 0.72 | 0.484 | 6 | 33 |
| WP: Senescence and Autophagy in Cancer | 0.69 | 0.497 | 17 | 105 |
| KEGG: Thiamine metabolism | 0.69 | 0.477 | 3 | 15 |
| WP: miRNAs involved in DNA damage response | 0.69 | 0.495 | 3 | 15 |
| WP: Phytochemical activity on NRF2 transcriptional activation | 0.69 | 0.605 | 3 | 15 |
| WP: Biogenic Amine Synthesis | 0.69 | 0.484 | 3 | 15 |

| | | | | |
|---|---|---|---|---|
| WP: Estrogen metabolism | 0.69 | 0.505 | 3 | 15 |
| WP: Kit receptor signaling pathway | 0.68 | 0.515 | 10 | 59 |
| WP: TYROBP Causal Network | 0.68 | 0.524 | 10 | 59 |
| KEGG: Ras signaling pathway | 0.68 | 0.502 | 35 | 227 |
| KEGG: Carbohydrate digestion and absorption | 0.66 | 0.518 | 7 | 40 |
| KEGG: Fat digestion and absorption | 0.66 | 0.526 | 7 | 40 |
| KEGG: Neurotrophin signaling pathway | 0.66 | 0.518 | 19 | 119 |
| KEGG: Wnt signaling pathway | 0.66 | 0.481 | 23 | 146 |
| Reactome: DDX58/IFIH1-mediated induction of interferon-alpha/beta | 0.66 | 0.522 | 11 | 66 |
| Reactome: NR1D1 (REV-ERBA) represses gene expression | 0.64 | 0.51 | 1 | 4 |
| Reactome: Threonine catabolism | 0.64 | 0.524 | 1 | 4 |
| Reactome: Signaling by MST1 | 0.64 | 0.592 | 1 | 4 |
| Reactome: rRNA processing | 0.64 | 0.572 | 1 | 4 |
| Reactome: Intestinal absorption | 0.64 | 0.518 | 1 | 4 |
| Reactome: NGF processing | 0.64 | 0.578 | 1 | 4 |
| Reactome: Biosynthesis of DPA-derived SPMs | 0.64 | 0.563 | 1 | 4 |
| WP: miR-222 in Exercise-Induced Cardiac Growth | 0.64 | 0.396 | 1 | 4 |
| WP: Serotonin Receptor 2 and STAT3 Signaling | 0.64 | 0.46 | 1 | 4 |
| WP: Angiopoietin Like Protein 8 Regulatory Pathway | 0.64 | 0.536 | 21 | 133 |
| Reactome: Striated Muscle Contraction | 0.64 | 0.563 | 6 | 34 |
| WP: p38 MAPK Signaling Pathway | 0.64 | 0.554 | 6 | 34 |
| KEGG: Pancreatic secretion | 0.63 | 0.561 | 15 | 93 |
| KEGG: Endocrine and other factor-regulated calcium reabsorption | 0.62 | 0.566 | 8 | 47 |
| WP: Constitutive Androstane Receptor Pathway | 0.61 | 0.564 | 5 | 28 |
| Reactome: G alpha (s) signalling events | 0.61 | 0.545 | 38 | 250 |
| WP: PPAR signaling pathway | 0.6 | 0.575 | 11 | 67 |
| KEGG: NF-kappa B signaling pathway | 0.6 | 0.525 | 14 | 87 |
| KEGG: Protein digestion and absorption | 0.6 | 0.509 | 14 | 87 |
| Reactome: Signaling by MET | 0.59 | 0.555 | 9 | 54 |
| Reactome: Regulation of TP53 Expression and Degradation | 0.59 | 0.55 | 7 | 41 |
| WP: TNF related weak inducer of apoptosis (TWEAK) Signaling Pathway | 0.59 | 0.546 | 7 | 41 |
| WP: ATM Signaling Pathway | 0.59 | 0.516 | 7 | 41 |
| KEGG: Chemokine signaling pathway | 0.59 | 0.525 | 28 | 182 |
| KEGG: Pentose and glucuronate interconversions | 0.58 | 0.594 | 4 | 22 |
| KEGG: PPAR signaling pathway | 0.58 | 0.544 | 12 | 74 |
| KEGG: Cortisol synthesis and secretion | 0.57 | 0.569 | 10 | 61 |
| Reactome: Costimulation by the CD28 family | 0.57 | 0.555 | 10 | 61 |
| Reactome: Transcriptional regulation by RUNX2 | 0.57 | 0.556 | 10 | 61 |
| Reactome: Biosynthesis of DHA-derived SPMs | 0.56 | 0.592 | 3 | 16 |
| Reactome: Neurotransmitter clearance | 0.56 | 0.599 | 2 | 10 |
| Reactome: Tetrahydrobiopterin (BH4) synthesis, recycling, salvage and regulation | 0.56 | 0.598 | 2 | 10 |
| WP: Liver X Receptor Pathway | 0.56 | 0.757 | 2 | 10 |
| Reactome: MyD88:MAL(TIRAP) cascade initiated on plasma membrane | 0.56 | 0.577 | 6 | 35 |
| WP: Pancreatic adenocarcinoma pathway | 0.55 | 0.584 | 14 | 88 |
| WP: Sterol Regulatory Element-Binding Proteins (SREBP) signalling | 0.55 | 0.607 | 11 | 68 |
| WP: RAC1/PAK1/p38/MMP2 Pathway | 0.55 | 0.58 | 11 | 68 |
| KEGG: Metabolism of xenobiotics by cytochrome P450 | 0.51 | 0.577 | 10 | 62 |
| WP: Genotoxicity pathway | 0.51 | 0.613 | 10 | 62 |
| KEGG: Arginine and proline metabolism | 0.5 | 0.633 | 8 | 49 |
| Reactome: Netrin-1 signaling | 0.5 | 0.622 | 8 | 49 |
| KEGG: Renin-angiotensin system | 0.49 | 0.646 | 4 | 23 |
| KEGG: Vitamin digestion and absorption | 0.49 | 0.614 | 4 | 23 |
| WP: EBV LMP1 signaling | 0.49 | 0.611 | 4 | 23 |
| WP: Blood Clotting Cascade | 0.49 | 0.566 | 4 | 23 |
| KEGG: TGF-beta signaling pathway | 0.47 | 0.692 | 13 | 83 |
| WP: Amino Acid metabolism | 0.46 | 0.631 | 14 | 90 |
| WP: T-Cell antigen Receptor (TCR) Signaling Pathway | 0.46 | 0.65 | 14 | 90 |
| KEGG: Inflammatory mediator regulation of TRP channels | 0.45 | 0.657 | 15 | 97 |
| KEGG: Ascorbate and aldarate metabolism | 0.45 | 0.629 | 3 | 17 |
| Reactome: Gastrin-CREB signalling pathway via PKC and MAPK | 0.45 | 0.648 | 3 | 17 |
| Reactome: RUNX1 and FOXP3 control the development of regulatory T lymphocytes (Tregs) | 0.45 | 0.618 | 3 | 17 |
| Reactome: Regulation of RUNX1 Expression and Activity | 0.45 | 0.662 | 3 | 17 |
| WP: IL-9 Signaling Pathway | 0.45 | 0.676 | 3 | 17 |
| WP: Drug Induction of Bile Acid Pathway | 0.45 | 0.605 | 3 | 17 |
| WP: miR-509-3p alteration of YAP1/ECM axis | 0.45 | 0.654 | 3 | 17 |
| WP: Gastric Cancer Network 2 | 0.44 | 0.691 | 5 | 30 |
| WP: PI3K-AKT-mTOR signaling pathway and therapeutic opportunities | 0.44 | 0.675 | 5 | 30 |
| Reactome: Platelet homeostasis | 0.43 | 0.678 | 12 | 77 |
| WP: Pathways in clear cell renal cell carcinoma | 0.42 | 0.681 | 13 | 84 |
| KEGG: Progesterone-mediated oocyte maturation | 0.42 | 0.709 | 14 | 91 |
| KEGG: Taurine and hypotaurine metabolism | 0.41 | 0.684 | 2 | 11 |
| Reactome: Platelet Aggregation (Plug Formation) | 0.41 | 0.647 | 2 | 11 |
| Reactome: Peptide hormone biosynthesis | 0.41 | 0.652 | 2 | 11 |
| Reactome: SUMOylation of immune response proteins | 0.41 | 0.666 | 2 | 11 |
| WP: NAD metabolism, sirtuins and aging | 0.41 | 0.68 | 2 | 11 |

| | | | | |
|---|---|---|---|---|
| Reactome: NCAM signaling for neurite out-growth | 0.41 | 0.707 | 6 | 37 |
| Reactome: Signaling by NOTCH2 | 0.41 | 0.723 | 6 | 37 |
| KEGG: Parathyroid hormone synthesis, secretion and action | 0.4 | 0.713 | 16 | 105 |
| Reactome: Clathrin-mediated endocytosis | 0.4 | 0.696 | 16 | 105 |
| KEGG: D-Glutamine and D-glutamate metabolism | 0.4 | 0.704 | 1 | 5 |
| Reactome: Transmission across Electrical Synapses | 0.4 | 0.741 | 1 | 5 |
| Reactome: Reelin signalling pathway | 0.4 | 0.737 | 1 | 5 |
| Reactome: Biosynthesis of EPA-derived SPMs | 0.4 | 0.694 | 1 | 5 |
| WP: Secretion of Hydrochloric Acid in Parietal Cells | 0.4 | 0.738 | 1 | 5 |
| WP: Synthesis and Degradation of Ketone Bodies | 0.4 | 0.777 | 1 | 5 |
| WP: miR-517 relationship with ARCN1 and USP1 | 0.4 | 0.928 | 1 | 5 |
| Reactome: Interleukin-20 family signaling | 0.4 | 0.693 | 4 | 24 |
| Reactome: SUMOylation of intracellular receptors | 0.4 | 0.708 | 4 | 24 |
| KEGG: ABC transporters | 0.39 | 0.702 | 7 | 44 |
| Reactome: Signaling by EGFR | 0.39 | 0.703 | 7 | 44 |
| WP: Wnt Signaling Pathway | 0.37 | 0.729 | 8 | 51 |
| WP: miRNA regulation of prostate cancer signaling pathways | 0.36 | 0.731 | 5 | 31 |
| KEGG: Platelet activation | 0.36 | 0.737 | 18 | 120 |
| Reactome: Plasma lipoprotein assembly, remodeling, and clearance | 0.34 | 0.734 | 11 | 72 |
| Reactome: Synaptic adhesion-like molecules | 0.34 | 0.707 | 3 | 18 |
| KEGG: Pyruvate metabolism | 0.34 | 0.76 | 6 | 38 |
| WP: Nuclear Receptors | 0.34 | 0.762 | 6 | 38 |
| WP: Striated Muscle Contraction | 0.34 | 0.754 | 6 | 38 |
| WP: Glycolysis and Gluconeogenesis | 0.33 | 0.756 | 7 | 45 |
| KEGG: Glucagon signaling pathway | 0.33 | 0.766 | 15 | 100 |
| WP: DNA Damage Response (only ATM dependent) | 0.32 | 0.745 | 17 | 114 |
| Reactome: Glycerophospholipid biosynthesis | 0.32 | 0.738 | 18 | 121 |
| WP: Phosphodiesterases in neuronal function | 0.31 | 0.73 | 8 | 52 |
| WP: Chemokine signaling pathway | 0.31 | 0.76 | 24 | 163 |
| KEGG: Butanoate metabolism | 0.31 | 0.754 | 4 | 25 |
| Reactome: Deregulated CDK5 triggers multiple neurodegenerative pathways in Alzheimer's disease mode | 0.31 | 0.76 | 4 | 25 |
| WP: Hypothesized Pathways in Pathogenesis of Cardiovascular Disease | 0.31 | 0.75 | 4 | 25 |
| WP: The effect of progerin on the involved genes in Hutchinson-Gilford Progeria Syndrome | 0.31 | 0.787 | 4 | 25 |
| Reactome: Signaling by NOTCH3 | 0.31 | 0.78 | 9 | 59 |
| Reactome: Signaling by FGFR1 | 0.31 | 0.777 | 9 | 59 |
| Reactome: Neurexins and neuroligins | 0.31 | 0.757 | 9 | 59 |
| WP: Human Thyroid Stimulating Hormone (TSH) signaling pathway | 0.3 | 0.768 | 10 | 66 |
| Reactome: Transcriptional Regulation by MECP2 | 0.29 | 0.752 | 13 | 87 |
| WP: Retinoblastoma Gene in Cancer | 0.29 | 0.786 | 13 | 87 |
| KEGG: Melanogenesis | 0.28 | 0.798 | 15 | 101 |
| KEGG: Phagosome | 0.28 | 0.775 | 20 | 136 |
| KEGG: Fatty acid biosynthesis | 0.28 | 0.775 | 2 | 12 |
| Reactome: Presynaptic depolarization and calcium channel opening | 0.28 | 0.731 | 2 | 12 |
| Reactome: Erythrocytes take up carbon dioxide and release oxygen | 0.28 | 0.757 | 2 | 12 |
| Reactome: Butyrophilin (BTN) family interactions | 0.28 | 0.779 | 2 | 12 |
| WP: Alanine and aspartate metabolism | 0.28 | 0.782 | 2 | 12 |
| WP: Bone Morphogenic Protein (BMP) Signalling and Regulation | 0.28 | 0.847 | 2 | 12 |
| WP: Codeine and Morphine Metabolism | 0.28 | 0.77 | 2 | 12 |
| WP: RalA downstream regulated genes | 0.28 | 0.837 | 2 | 12 |
| Reactome: Histidine, lysine, phenylalanine, tyrosine, proline and tryptophan catabolism | 0.26 | 0.801 | 7 | 46 |
| KEGG: NOD-like receptor signaling pathway | 0.25 | 0.799 | 23 | 158 |
| KEGG: Taste transduction | 0.24 | 0.822 | 12 | 81 |
| KEGG: Thyroid hormone signaling pathway | 0.24 | 0.811 | 17 | 116 |
| KEGG: Pantothenate and CoA biosynthesis | 0.24 | 0.822 | 3 | 19 |
| KEGG: Folate biosynthesis | 0.22 | 0.837 | 4 | 26 |
| Reactome: WNT ligand biogenesis and trafficking | 0.22 | 0.838 | 4 | 26 |
| Reactome: Other interleukin signaling | 0.22 | 0.846 | 4 | 26 |
| Reactome: GPVI-mediated activation cascade | 0.21 | 0.829 | 5 | 33 |
| Reactome: Fatty acyl-CoA biosynthesis | 0.21 | 0.809 | 5 | 33 |
| WP: Fluoropyrimidine Activity | 0.21 | 0.827 | 5 | 33 |
| WP: Endothelin Pathways | 0.21 | 0.842 | 5 | 33 |
| KEGG: Ferroptosis | 0.21 | 0.847 | 6 | 40 |
| WP: IL-5 Signaling Pathway | 0.21 | 0.828 | 6 | 40 |
| WP: Aryl Hydrocarbon Receptor Pathway | 0.21 | 0.837 | 6 | 40 |
| WP: Ferroptosis | 0.21 | 0.834 | 6 | 40 |
| KEGG: Circadian entrainment | 0.2 | 0.861 | 14 | 96 |
| KEGG: Glutathione metabolism | 0.2 | 0.856 | 8 | 54 |
| WP: Leptin signaling pathway | 0.2 | 0.877 | 11 | 75 |
| Reactome: Uptake and function of diphtheria toxin | 0.2 | 0.792 | 1 | 6 |
| Reactome: Choline catabolism | 0.2 | 0.859 | 1 | 6 |
| WP: Effects of Nitric Oxide | 0.2 | 0.804 | 1 | 6 |
| KEGG: Tight junction | 0.19 | 0.846 | 24 | 167 |
| WP: TGF-beta Signaling Pathway | 0.17 | 0.855 | 19 | 132 |
| WP: Ebola Virus Pathway on Host | 0.17 | 0.843 | 18 | 125 |
| Reactome: Receptor-type tyrosine-protein phosphatases | 0.16 | 0.88 | 2 | 13 |

| | | | | |
|---|---|---|---|---|
| WP: MFAP5-mediated ovarian cancer cell motility and invasiveness | 0.16 | 0.869 | 2 | 13 |
| WP: BMP2-WNT4-FOXO1 Pathway in Human Primary Endometrial Stromal Cell Differentiation | 0.16 | 0.877 | 2 | 13 |
| WP: Development of pulmonary dendritic cells and macrophage subsets | 0.16 | 0.925 | 2 | 13 |
| WP: Serotonin and anxiety-related events | 0.16 | 0.882 | 2 | 13 |
| WP: Transcriptional cascade regulating adipogenesis | 0.16 | 0.923 | 2 | 13 |
| WP: Disorders of Folate Metabolism and Transport | 0.16 | 0.871 | 2 | 13 |
| WP: Vitamin B12 Disorders | 0.16 | 0.83 | 2 | 13 |
| WP: Apoptosis Modulation and Signaling | 0.16 | 0.875 | 13 | 90 |
| WP: Prolactin Signaling Pathway | 0.15 | 0.884 | 11 | 76 |
| KEGG: Cellular senescence | 0.15 | 0.862 | 22 | 154 |
| KEGG: Synaptic vesicle cycle | 0.15 | 0.891 | 9 | 62 |
| WP: BMP Signaling Pathway in Eyelid Development | 0.15 | 0.866 | 3 | 20 |
| KEGG: Cytosolic DNA-sensing pathway | 0.14 | 0.881 | 8 | 55 |
| WP: IL-1 signaling pathway | 0.14 | 0.896 | 8 | 55 |
| Reactome: Inositol phosphate metabolism | 0.14 | 0.901 | 7 | 48 |
| Reactome: Transcriptional regulation by the AP-2 (TFAP2) family of transcription factors | 0.14 | 0.87 | 7 | 48 |
| Reactome: Effects of PIP2 hydrolysis | 0.14 | 0.891 | 4 | 27 |
| Reactome: Signaling by ERBB4 | 0.14 | 0.895 | 6 | 41 |
| WP: Signaling of Hepatocyte Growth Factor Receptor | 0.14 | 0.915 | 5 | 34 |
| KEGG: Cholinergic synapse | 0.13 | 0.926 | 16 | 112 |
| WP: EGF/EGFR Signaling Pathway | 0.12 | 0.919 | 23 | 162 |
| Reactome: G alpha (12/13) signalling events | 0.1 | 0.933 | 11 | 77 |
| WP: MECP2 and Associated Rett Syndrome | 0.09 | 0.928 | 9 | 63 |
| WP: Endometrial cancer | 0.09 | 0.909 | 9 | 63 |
| KEGG: Fatty acid elongation | 0.06 | 0.949 | 4 | 28 |
| KEGG: Hippo signaling pathway | 0.06 | 0.956 | 4 | 28 |
| Reactome: Signaling by BMP | 0.06 | 0.942 | 4 | 28 |
| Reactome: Pre-NOTCH Expression and Processing | 0.06 | 0.947 | 11 | 78 |
| Reactome: Metabolism of nitric oxide | 0.04 | 0.975 | 2 | 14 |
| Reactome: Prolactin receptor signaling | 0.04 | 0.976 | 2 | 14 |
| Reactome: Caspase activation via Death Receptors in the presence of ligand | 0.04 | 0.968 | 2 | 14 |
| WP: ERK Pathway in Huntington's Disease | 0.04 | 0.937 | 2 | 14 |
| WP: Prader-Willi and Angelman Syndrome | 0.04 | 0.971 | 9 | 64 |
| KEGG: Phospholipase D signaling pathway | 0.04 | 0.966 | 20 | 143 |
| WP: Integrin-mediated Cell Adhesion | 0.04 | 0.962 | 14 | 100 |
| Reactome: Fructose metabolism | 0.03 | 0.999 | 1 | 7 |
| WP: Hypothetical Craniofacial Development Pathway | 0.03 | 0.977 | 1 | 7 |
| WP: Arachidonate Epoxygenase / Epoxide Hydrolase | 0.03 | 0.925 | 1 | 7 |
| KEGG: Aldosterone synthesis and secretion | 0.03 | 0.975 | 13 | 93 |
| KEGG: Relaxin signaling pathway | 0.03 | 0.99 | 18 | 129 |
| KEGG: Hepatocellular carcinoma | 0.02 | 0.975 | 23 | 165 |
| Reactome: Gene and protein expression by JAK-STAT signaling after Interleukin-12 stimulation | 0 | 0.992 | 10 | 72 |
| Reactome: Signaling by VEGF | 0 | 0.997 | 14 | 101 |
| WP: Cannabinoid receptor signaling | -0.01 | 0.999 | 4 | 29 |
| WP: PDGFR-beta pathway | -0.01 | 0.999 | 4 | 29 |
| KEGG: Longevity regulating pathway | -0.02 | 0.969 | 12 | 87 |
| KEGG: Fatty acid metabolism | -0.03 | 0.972 | 7 | 51 |
| Reactome: Apoptotic execution phase | -0.03 | 0.967 | 7 | 51 |
| KEGG: Terpenoid backbone biosynthesis | -0.03 | 0.965 | 3 | 22 |
| Reactome: Signaling by NTRK2 (TRKB) | -0.03 | 0.97 | 3 | 22 |
| Reactome: Metabolism of water-soluble vitamins and cofactors | -0.04 | 0.961 | 10 | 73 |
| Reactome: ESR-mediated signaling | -0.05 | 0.968 | 18 | 131 |
| KEGG: Fatty acid degradation | -0.05 | 0.967 | 6 | 44 |
| WP: Integrated Cancer Pathway | -0.05 | 0.976 | 6 | 44 |
| WP: Interleukin-11 Signaling Pathway | -0.05 | 0.965 | 6 | 44 |
| WP: Non-small cell lung cancer | -0.06 | 0.972 | 9 | 66 |
| Reactome: Regulation of TP53 Activity through Association with Co-factors | -0.06 | 0.912 | 2 | 15 |
| WP: Ganglio Sphingolipid Metabolism | -0.06 | 0.923 | 2 | 15 |
| WP: Role of Osx and miRNAs in tooth development | -0.06 | 0.922 | 2 | 15 |
| WP: GPCRs, Class C Metabotropic glutamate, pheromone | -0.06 | 0.927 | 2 | 15 |
| KEGG: mTOR signaling pathway | -0.06 | 0.964 | 20 | 146 |
| KEGG: cAMP signaling pathway | -0.07 | 0.949 | 27 | 197 |
| KEGG: Lysine degradation | -0.07 | 0.957 | 8 | 59 |
| KEGG: Glyoxylate and dicarboxylate metabolism | -0.09 | 0.914 | 4 | 30 |
| KEGG: Vascular smooth muscle contraction | -0.1 | 0.917 | 16 | 118 |
| WP: NO/cGMP/PKG mediated Neuroprotection | -0.11 | 0.929 | 6 | 45 |
| Reactome: Interleukin-17 signaling | -0.11 | 0.855 | 1 | 8 |
| WP: HIF1A and PPARG regulation of glycolysis | -0.11 | 0.87 | 1 | 8 |
| WP: Dual hijack model of Vif in HIV infection | -0.11 | 0.984 | 1 | 8 |
| WP: NRF2-ARE regulation | -0.12 | 0.888 | 3 | 23 |
| WP: Parkinsons Disease Pathway | -0.13 | 0.921 | 5 | 38 |
| WP: Oxidative Damage | -0.13 | 0.904 | 5 | 38 |
| WP: Endoderm Differentiation | -0.14 | 0.909 | 19 | 141 |
| KEGG: beta-Alanine metabolism | -0.16 | 0.877 | 4 | 31 |
| Reactome: MAPK targets/ Nuclear events mediated by MAP kinases | -0.16 | 0.876 | 4 | 31 |

| | | | | |
|---|---|---|---|---|
| Reactome: Metabolism of steroid hormones | -0.16 | 0.863 | 4 | 31 |
| WP: IL17 signaling pathway | -0.16 | 0.913 | 4 | 31 |
| WP: Trans-sulfuration and one carbon metabolism | -0.16 | 0.879 | 4 | 31 |
| Reactome: YAP1- and WWTR1 (TAZ)-stimulated gene expression | -0.16 | 0.841 | 2 | 16 |
| WP: Deregulation of Rab and Rab Effector Genes in Bladder Cancer | -0.16 | 0.872 | 2 | 16 |
| WP: Regulation of Actin Cytoskeleton | -0.16 | 0.863 | 20 | 149 |
| WP: Energy Metabolism | -0.16 | 0.846 | 6 | 46 |
| Reactome: Cargo recognition for clathrin-mediated endocytosis | -0.16 | 0.873 | 11 | 83 |
| WP: Viral Acute Myocarditis | -0.16 | 0.871 | 11 | 83 |
| KEGG: Glycerolipid metabolism | -0.17 | 0.876 | 8 | 61 |
| Reactome: ISG15 antiviral mechanism | -0.17 | 0.856 | 8 | 61 |
| WP: MAPK Signaling Pathway | -0.19 | 0.848 | 33 | 245 |
| WP: ErbB Signaling Pathway | -0.19 | 0.865 | 12 | 91 |
| WP: PDGF Pathway | -0.19 | 0.831 | 5 | 39 |
| Reactome: Miscellaneous transport and binding events | -0.2 | 0.833 | 3 | 24 |
| Reactome: Signaling by Erythropoietin | -0.2 | 0.851 | 3 | 24 |
| WP: Physiological and Pathological Hypertrophy  of the Heart | -0.2 | 0.866 | 3 | 24 |
| Reactome: Keratinization | -0.2 | 0.831 | 9 | 69 |
| KEGG: Insulin secretion | -0.21 | 0.84 | 11 | 84 |
| WP: Exercise-induced Circadian Regulation | -0.22 | 0.828 | 6 | 47 |
| Reactome: RAB geranylgeranylation | -0.22 | 0.846 | 8 | 62 |
| Reactome: Carboxyterminal post-translational modifications of tubulin | -0.23 | 0.829 | 4 | 32 |
| WP: Ovarian Infertility Genes | -0.23 | 0.856 | 4 | 32 |
| Reactome: GP1b-IX-V activation signalling | -0.24 | 0.769 | 1 | 9 |
| Reactome: Serine biosynthesis | -0.24 | 0.824 | 1 | 9 |
| WP: Leptin and adiponectin | -0.24 | 0.84 | 1 | 9 |
| WP: Pyrimidine metabolism and related diseases | -0.24 | 0.83 | 1 | 9 |
| WP: Thiamine metabolic pathways | -0.24 | 0.833 | 1 | 9 |
| WP: Mismatch repair | -0.24 | 0.954 | 1 | 9 |
| WP: Heme Biosynthesis | -0.24 | 0.837 | 1 | 9 |
| KEGG: Selenocompound metabolism | -0.25 | 0.789 | 2 | 17 |
| Reactome: Aflatoxin activation and detoxification | -0.25 | 0.818 | 2 | 17 |
| WP: Canonical and Non-Canonical TGF-B signaling | -0.25 | 0.738 | 2 | 17 |
| WP: Simplified Depiction of MYD88 Distinct Input-Output Pathway | -0.25 | 0.753 | 2 | 17 |
| KEGG: Intestinal immune network for IgA production | -0.25 | 0.793 | 5 | 40 |
| KEGG: Human cytomegalovirus infection | -0.26 | 0.769 | 28 | 211 |
| Reactome: Fertilization | -0.27 | 0.77 | 3 | 25 |
| Reactome: MyD88 cascade initiated on plasma membrane | -0.27 | 0.817 | 3 | 25 |
| Reactome: PIP3 activates AKT signaling | -0.28 | 0.791 | 14 | 108 |
| KEGG: Valine, leucine and isoleucine degradation | -0.28 | 0.78 | 6 | 48 |
| KEGG: Cell cycle | -0.28 | 0.771 | 16 | 123 |
| KEGG: Fructose and mannose metabolism | -0.29 | 0.765 | 4 | 33 |
| Reactome: Cargo concentration in the ER | -0.29 | 0.785 | 4 | 33 |
| WP: BDNF-TrkB Signaling | -0.29 | 0.761 | 4 | 33 |
| WP: Prion disease pathway | -0.29 | 0.748 | 4 | 33 |
| Reactome: GABA receptor activation | -0.3 | 0.764 | 7 | 56 |
| Reactome: Signaling by Rho GTPases | -0.3 | 0.788 | 17 | 131 |
| Reactome: p75 NTR receptor-mediated signalling | -0.31 | 0.762 | 12 | 94 |
| KEGG: Natural killer cell mediated cytotoxicity | -0.31 | 0.778 | 14 | 109 |
| KEGG: Fc epsilon RI signaling pathway | -0.32 | 0.753 | 8 | 64 |
| WP: Photodynamic therapy-induced AP-1 survival signaling. | -0.33 | 0.766 | 6 | 49 |
| KEGG: C-type lectin receptor signaling pathway | -0.33 | 0.732 | 13 | 102 |
| KEGG: p53 signaling pathway | -0.34 | 0.742 | 9 | 72 |
| Reactome: Nephrin family interactions | -0.34 | 0.713 | 2 | 18 |
| Reactome: SUMOylation of transcription factors | -0.34 | 0.764 | 2 | 18 |
| Reactome: Digestion | -0.34 | 0.723 | 2 | 18 |
| WP: 4-hydroxytamoxifen, Dexamethasone, and Retinoic Acids Regulation of p27 Expression | -0.34 | 0.763 | 2 | 18 |
| WP: EPO Receptor Signaling | -0.35 | 0.759 | 3 | 26 |
| KEGG: VEGF signaling pathway | -0.35 | 0.719 | 7 | 57 |
| KEGG: Long-term depression | -0.35 | 0.721 | 7 | 57 |
| Reactome: mRNA Editing | -0.35 | 0.728 | 1 | 10 |
| Reactome: Trafficking and processing of endosomal TLR | -0.35 | 0.715 | 1 | 10 |
| Reactome: Neurotransmitter receptors and postsynaptic signal transmission | -0.35 | 0.711 | 1 | 10 |
| Reactome: SUMOylation | -0.35 | 0.715 | 1 | 10 |
| Reactome: Caspase activation via Dependence Receptors in the absence of ligand | -0.35 | 0.714 | 1 | 10 |
| Reactome: Ketone body metabolism | -0.35 | 0.705 | 1 | 10 |
| WP: Type III interferon signaling | -0.35 | 0.922 | 1 | 10 |
| WP: Irinotecan Pathway | -0.35 | 0.713 | 1 | 10 |
| WP: Trans-sulfuration pathway | -0.35 | 0.708 | 1 | 10 |
| WP: Composition of Lipid Particles | -0.35 | 0.694 | 1 | 10 |
| WP: Interleukin-1 Induced Activation of NF-kappa-B | -0.35 | 0.863 | 1 | 10 |
| WP: Steroid Biosynthesis | -0.35 | 0.722 | 1 | 10 |
| Reactome: Signaling by FGFR3 | -0.36 | 0.722 | 4 | 34 |
| Reactome: PI3K Cascade | -0.36 | 0.716 | 4 | 34 |
| WP: GABA receptor Signaling | -0.36 | 0.71 | 4 | 34 |

| | | | | |
|---|---|---|---|---|
| WP: Pathways Affected in Adenoid Cystic Carcinoma | -0.37 | 0.688 | 8 | 65 |
| Reactome: Transport of inorganic cations/anions and amino acids/oligopeptides | -0.37 | 0.739 | 13 | 103 |
| Reactome: Sphingolipid metabolism | -0.4 | 0.703 | 10 | 81 |
| KEGG: Apelin signaling pathway | -0.4 | 0.695 | 17 | 134 |
| Reactome: Abnormal conversion of 2-oxoglutarate to 2-hydroxyglutarate | -0.4 | 0.831 | 0 | 1 |
| Reactome: NADPH regeneration | -0.4 | 0.743 | 0 | 1 |
| Reactome: Vitamin E | -0.4 | 0.724 | 0 | 1 |
| WP: Influenza A virus infection | -0.4 | 0.694 | 0 | 1 |
| WP: Colchicine Metabolic Pathway | -0.4 | 0.675 | 0 | 1 |
| WP: mir34a and TGIF2 in osteoclastogenesis | -0.4 | 0.679 | 0 | 1 |
| WP: Gut-Liver Indole Metabolism | -0.4 | 0.821 | 0 | 1 |
| WP: Metabolism of Dichloroethylene by CYP450 | -0.4 | 0.76 | 0 | 1 |
| WP: Acrylamide Biotransformation and Exposure Biomarkers | -0.4 | 0.822 | 0 | 1 |
| WP: FABP4 in ovarian cancer | -0.4 | 0.388 | 0 | 1 |
| WP: Glucose Homeostasis | -0.4 | 0.669 | 0 | 1 |
| WP: Cell Cycle | -0.4 | 0.687 | 15 | 119 |
| WP: Association Between Physico-Chemical Features and Toxicity Associated Pathways | -0.41 | 0.694 | 8 | 66 |
| KEGG: Glycosphingolipid biosynthesis | -0.42 | 0.653 | 3 | 27 |
| WP: Follicle Stimulating Hormone (FSH) signaling pathway | -0.42 | 0.681 | 3 | 27 |
| Reactome: Toll Like Receptor 3 (TLR3) Cascade | -0.42 | 0.684 | 4 | 35 |
| Reactome: Smooth Muscle Contraction | -0.42 | 0.694 | 4 | 35 |
| KEGG: Arginine biosynthesis | -0.42 | 0.675 | 2 | 19 |
| KEGG: One carbon pool by folate | -0.42 | 0.675 | 2 | 19 |
| Reactome: Signaling by Hippo | -0.42 | 0.664 | 2 | 19 |
| Reactome: Synthesis, secretion, and deacylation of Ghrelin | -0.42 | 0.677 | 2 | 19 |
| WP: Nucleotide Metabolism | -0.42 | 0.659 | 2 | 19 |
| WP: Hereditary leiomyomatosis and renal cell carcinoma pathway | -0.42 | 0.662 | 2 | 19 |
| WP: Serotonin Receptor 4/6/7 and NR3C Signaling | -0.42 | 0.692 | 2 | 19 |
| WP: IL-6 signaling pathway | -0.43 | 0.676 | 5 | 43 |
| KEGG: Inositol phosphate metabolism | -0.43 | 0.672 | 9 | 74 |
| KEGG: Gastric acid secretion | -0.43 | 0.658 | 9 | 74 |
| KEGG: Insulin signaling pathway | -0.43 | 0.684 | 17 | 135 |
| Reactome: Nucleotide-binding domain, leucine rich repeat containing receptor (NLR) signaling pathways | -0.44 | 0.663 | 6 | 51 |
| Reactome: EPH-Ephrin signaling | -0.46 | 0.668 | 11 | 90 |
| Reactome: Mitotic G1-G1/S phases | -0.46 | 0.654 | 21 | 166 |
| KEGG: Ubiquinone and other terpenoid-quinone biosynthesis | -0.46 | 0.657 | 1 | 11 |
| Reactome: Signal regulatory protein family interactions | -0.46 | 0.628 | 1 | 11 |
| Reactome: RHO GTPases activate IQGAPs | -0.46 | 0.606 | 1 | 11 |
| Reactome: Vitamin D (calciferol) metabolism | -0.46 | 0.662 | 1 | 11 |
| WP: Nanoparticle triggered regulated necrosis | -0.46 | 0.622 | 1 | 11 |
| KEGG: HIF-1 signaling pathway | -0.47 | 0.63 | 12 | 98 |
| Reactome: MyD88-independent TLR4 cascade | -0.48 | 0.638 | 4 | 36 |
| KEGG: Sphingolipid metabolism | -0.48 | 0.651 | 5 | 44 |
| Reactome: Energy dependent regulation of mTOR by LKB1-AMPK | -0.48 | 0.658 | 3 | 28 |
| Reactome: TNFs bind their physiological receptors | -0.48 | 0.655 | 3 | 28 |
| WP: Sphingolipid Metabolism | -0.48 | 0.626 | 3 | 28 |
| WP: TLR4 Signaling and Tolerance | -0.48 | 0.625 | 3 | 28 |
| WP: Lipid Metabolism Pathway | -0.48 | 0.607 | 3 | 28 |
| WP: Pathogenic Escherichia coli infection | -0.49 | 0.621 | 6 | 52 |
| KEGG: Axon guidance | -0.5 | 0.614 | 22 | 175 |
| Reactome: RORA activates gene expression | -0.5 | 0.618 | 2 | 20 |
| WP: Type II diabetes mellitus | -0.5 | 0.605 | 2 | 20 |
| WP: Serotonin Receptor 2 and ELK-SRF/GATA4 signaling | -0.5 | 0.617 | 2 | 20 |
| WP: DNA Damage Response | -0.5 | 0.591 | 8 | 68 |
| KEGG: Phosphatidylinositol signaling system | -0.51 | 0.585 | 12 | 99 |
| Reactome: Signaling by NTRK1 (TRKA) | -0.51 | 0.593 | 9 | 76 |
| Reactome: Regulation of Hypoxia-inducible Factor (HIF) by oxygen | -0.51 | 0.597 | 9 | 76 |
| Reactome: Transport of bile salts and organic acids, metal ions and amine compounds | -0.52 | 0.589 | 10 | 84 |
| WP: Cardiac Hypertrophic Response | -0.54 | 0.589 | 6 | 53 |
| KEGG: Pentose phosphate pathway | -0.55 | 0.582 | 3 | 29 |
| Reactome: Surfactant metabolism | -0.55 | 0.596 | 3 | 29 |
| WP: One Carbon Metabolism | -0.55 | 0.571 | 3 | 29 |
| WP: Canonical  and Non-canonical Notch signaling | -0.55 | 0.609 | 3 | 29 |
| WP: Toll-like Receptor Signaling | -0.55 | 0.65 | 3 | 29 |
| WP: MAPK  and NFkB Signalling Pathways Inhibited by Yersinia YopJ | -0.56 | 0.746 | 1 | 12 |
| WP: Valproic acid pathway | -0.56 | 0.615 | 1 | 12 |
| Reactome: Signaling by NOTCH1 | -0.56 | 0.567 | 9 | 77 |
| KEGG: ErbB signaling pathway | -0.56 | 0.596 | 10 | 85 |
| KEGG: Th1 and Th2 cell differentiation | -0.56 | 0.582 | 10 | 85 |
| Reactome: RHO GTPases Activate Formins | -0.56 | 0.562 | 14 | 116 |
| Reactome: Transport of glycerol from adipocytes to the liver by Aquaporins | -0.57 | 0.589 | 0 | 2 |
| Reactome: Pyrophosphate hydrolysis | -0.57 | 0.715 | 0 | 2 |
| Reactome: Plasmalogen biosynthesis | -0.57 | 0.595 | 0 | 2 |
| Reactome: Intestinal infectious diseases | -0.57 | 0.516 | 0 | 2 |
| WP: Butyrate-induced histone acetylation | -0.57 | 0.735 | 0 | 2 |

| | | | | |
|---|---|---|---|---|
| WP: Aripiprazole Metabolic Pathway | -0.57 | 0.588 | 0 | 2 |
| WP: Lidocaine metabolism | -0.57 | 0.727 | 0 | 2 |
| WP: Felbamate Metabolism | -0.57 | 0.73 | 0 | 2 |
| WP: Hormonal control of Pubertal Growth Spurt | -0.57 | 0.664 | 0 | 2 |
| WP: Ultraconserved region 339 modulation of tumor suppressor microRNAs in cancer | -0.57 | 0.505 | 0 | 2 |
| WP: Model for regulation of MSMP expression in cancer cells and its proangiogenic role in ovarian tumors | -0.57 | 0.495 | 0 | 2 |
| WP: TP53 Network | -0.58 | 0.554 | 2 | 21 |
| WP: Interferon type I signaling pathways | -0.59 | 0.564 | 6 | 54 |
| Reactome: Signaling by PTK6 | -0.59 | 0.541 | 5 | 46 |
| WP: Integrated Breast Cancer Pathway | -0.6 | 0.542 | 20 | 163 |
| Reactome: MAP kinase activation | -0.6 | 0.54 | 4 | 38 |
| Reactome: RAF/MAP kinase cascade | -0.63 | 0.52 | 26 | 210 |
| WP: Histone Modifications | -0.64 | 0.516 | 7 | 63 |
| Reactome: RUNX1 regulates genes involved in megakaryocyte differentiation and platelet function | -0.64 | 0.533 | 8 | 71 |
| WP: miRNA Regulation of DNA Damage Response | -0.64 | 0.531 | 8 | 71 |
| KEGG: Cysteine and methionine metabolism | -0.64 | 0.517 | 5 | 47 |
| Reactome: Mitochondrial calcium ion transport | -0.65 | 0.495 | 1 | 13 |
| KEGG: Other types of O-glycan biosynthesis | -0.65 | 0.541 | 2 | 22 |
| KEGG: Oxytocin signaling pathway | -0.67 | 0.528 | 18 | 150 |
| KEGG: Propanoate metabolism | -0.68 | 0.499 | 3 | 31 |
| Reactome: Signal amplification | -0.68 | 0.506 | 3 | 31 |
| Reactome: Thrombin signalling through proteinase activated receptors (PARs) | -0.68 | 0.523 | 3 | 31 |
| Reactome: Sialic acid metabolism | -0.68 | 0.492 | 3 | 31 |
| Reactome: ROS, RNS production in phagocytes | -0.68 | 0.536 | 3 | 31 |
| WP: G1 to S cell cycle control | -0.68 | 0.505 | 7 | 64 |
| KEGG: Antigen processing and presentation | -0.69 | 0.485 | 6 | 56 |
| KEGG: Adrenergic signaling in cardiomyocytes | -0.69 | 0.508 | 17 | 143 |
| KEGG: Amino sugar and nucleotide sugar metabolism | -0.69 | 0.511 | 5 | 48 |
| Reactome: RHO GTPases regulate CFTR trafficking | -0.7 | 0.554 | 0 | 3 |
| Reactome: Wax biosynthesis | -0.7 | 0.552 | 0 | 3 |
| Reactome: Metabolism of vitamin K | -0.7 | 0.556 | 0 | 3 |
| Reactome: Intracellular oxygen transport | -0.7 | 0.553 | 0 | 3 |
| Reactome: Lactose synthesis | -0.7 | 0.534 | 0 | 3 |
| WP: Heroin metabolism | -0.7 | 0.389 | 0 | 3 |
| Reactome: Activation of NMDA receptors and postsynaptic events | -0.71 | 0.452 | 4 | 40 |
| WP: Nucleotide-binding Oligomerization Domain (NOD) pathway | -0.71 | 0.479 | 4 | 40 |
| Reactome: Branched-chain amino acid catabolism | -0.72 | 0.482 | 2 | 23 |
| Reactome: Nucleotide salvage | -0.72 | 0.472 | 2 | 23 |
| KEGG: Glycosaminoglycan biosynthesis | -0.73 | 0.571 | 1 | 14 |
| KEGG: Glycosphingolipid biosynthesis | -0.73 | 0.569 | 1 | 14 |
| Reactome: Pentose phosphate pathway | -0.73 | 0.434 | 1 | 14 |
| Reactome: Interleukin-15 signaling | -0.73 | 0.453 | 1 | 14 |
| WP: H19 action Rb-E2F1 signaling and CDK-Beta-catenin activity | -0.73 | 0.57 | 1 | 14 |
| KEGG: Porphyrin and chlorophyll metabolism | -0.74 | 0.467 | 3 | 32 |
| Reactome: DNA Damage Bypass | -0.74 | 0.451 | 5 | 49 |
| Reactome: Fcgamma receptor (FCGR) dependent phagocytosis | -0.76 | 0.441 | 9 | 82 |
| Reactome: Senescence-Associated Secretory Phenotype (SASP) | -0.76 | 0.458 | 9 | 82 |
| WP: MicroRNAs in cardiomyocyte hypertrophy | -0.76 | 0.411 | 9 | 82 |
| Reactome: Signaling by ERBB2 | -0.76 | 0.474 | 4 | 41 |
| WP: Insulin Signaling | -0.76 | 0.453 | 19 | 161 |
| WP: Triacylglyceride Synthesis | -0.79 | 0.453 | 2 | 24 |
| WP: miRNA regulation of p53 pathway in prostate cancer | -0.79 | 0.418 | 2 | 24 |
| Reactome: Transcriptional regulation of pluripotent stem cells | -0.8 | 0.424 | 3 | 33 |
| KEGG: GnRH signaling pathway | -0.8 | 0.432 | 10 | 91 |
| Reactome: PI Metabolism | -0.8 | 0.448 | 9 | 83 |
| KEGG: Caffeine metabolism | -0.8 | 0.459 | 0 | 4 |
| Reactome: TET1,2,3 and TDG demethylate DNA | -0.8 | 0.458 | 0 | 4 |
| Reactome: Lysosomal oligosaccharide catabolism | -0.8 | 0.506 | 0 | 4 |
| Reactome: Transcription from mitochondrial promoters | -0.8 | 0.418 | 0 | 4 |
| WP: Peroxisomal beta-oxidation of tetracosanoyl-CoA | -0.8 | 0.502 | 0 | 4 |
| WP: Diclofenac Metabolic Pathway | -0.8 | 0.391 | 0 | 4 |
| WP: Cocaine metabolism | -0.8 | 0.508 | 0 | 4 |
| WP: Oxytocin signaling | -0.8 | 0.354 | 0 | 4 |
| WP: eIF5A regulation in response to inhibition of the nuclear export system | -0.8 | 0.227 | 0 | 4 |
| WP: Caffeine and Theobromine metabolism | -0.8 | 0.476 | 0 | 4 |
| WP: MicroRNA network associated with chronic lymphocytic leukemia | -0.8 | 0.337 | 0 | 4 |
| WP: Amino acid conjugation of benzoic acid | -0.8 | 0.421 | 0 | 4 |
| WP: Polyol Pathway | -0.8 | 0.429 | 0 | 4 |
| WP: Ras Signaling | -0.81 | 0.438 | 21 | 178 |
| Reactome: Metabolism of porphyrins | -0.81 | 0.4 | 1 | 15 |
| Reactome: Mismatch Repair | -0.81 | 0.406 | 1 | 15 |
| WP: GPR40 Pathway | -0.81 | 0.501 | 1 | 15 |
| WP: Regulation of sister chromatid separation at the metaphase-anaphase transition | -0.81 | 0.461 | 1 | 15 |
| Reactome: Activation of gene expression by SREBF (SREBP) | -0.82 | 0.443 | 6 | 59 |
| Reactome: Translocation of SLC2A4 (GLUT4) to the plasma membrane | -0.84 | 0.407 | 5 | 51 |

| | | | | |
|---|---|---|---|---|
| Reactome: SIRT1 negatively regulates rRNA expression | -0.85 | 0.411 | 3 | 34 |
| WP: Type 2 papillary renal cell carcinoma | -0.85 | 0.394 | 3 | 34 |
| Reactome: Interleukin-3, Interleukin-5 and GM-CSF signaling | -0.87 | 0.376 | 4 | 43 |
| WP: T-Cell antigen Receptor (TCR) pathway during Staphylococcus aureus infection | -0.87 | 0.404 | 6 | 60 |
| KEGG: Gap junction | -0.88 | 0.383 | 9 | 85 |
| KEGG: 2-Oxocarboxylic acid metabolism | -0.88 | 0.4 | 1 | 16 |
| Reactome: Metabolism of folate and pterines | -0.88 | 0.406 | 1 | 16 |
| Reactome: Processing of SMDT1 | -0.88 | 0.41 | 1 | 16 |
| WP: Cori Cycle | -0.88 | 0.406 | 1 | 16 |
| WP: TCA Cycle and Deficiency of Pyruvate Dehydrogenase complex (PDHc) | -0.88 | 0.369 | 1 | 16 |
| WP: NAD+ metabolism | -0.88 | 0.386 | 1 | 16 |
| WP: ID signaling pathway | -0.88 | 0.503 | 1 | 16 |
| KEGG: Phenylalanine, tyrosine and tryptophan biosynthesis | -0.9 | 0.341 | 0 | 5 |
| Reactome: FasL/ CD95L signaling | -0.9 | 0.42 | 0 | 5 |
| Reactome: MTF1 activates gene expression | -0.9 | 0.35 | 0 | 5 |
| Reactome: Formation of xylulose-5-phosphate | -0.9 | 0.401 | 0 | 5 |
| Reactome: rRNA modification in the mitochondrion | -0.9 | 0.407 | 0 | 5 |
| WP: Sulindac Metabolic Pathway | -0.9 | 0.282 | 0 | 5 |
| WP: TCA Cycle Nutrient Utilization and Invasiveness of Ovarian Cancer | -0.9 | 0.287 | 0 | 5 |
| WP: Vitamin B6-dependent and responsive disorders | -0.9 | 0.389 | 0 | 5 |
| WP: Lamin A-processing pathway | -0.9 | 0.22 | 0 | 5 |
| WP: Arylamine metabolism | -0.9 | 0.315 | 0 | 5 |
| KEGG: Collecting duct acid secretion | -0.91 | 0.358 | 2 | 26 |
| KEGG: Other glycan degradation | -0.95 | 0.391 | 1 | 17 |
| Reactome: Protein methylation | -0.95 | 0.388 | 1 | 17 |
| WP: Mitochondrial LC-Fatty Acid Beta-Oxidation | -0.95 | 0.404 | 1 | 17 |
| Reactome: Gamma carboxylation, hypusine formation and arylsulfatase activation | -0.96 | 0.333 | 3 | 36 |
| Reactome: Oncogene Induced Senescence | -0.96 | 0.374 | 3 | 36 |
| Reactome: Signaling by FGFR4 | -0.96 | 0.338 | 3 | 36 |
| Reactome: Triglyceride metabolism | -0.96 | 0.337 | 3 | 36 |
| WP: Alzheimers Disease | -0.97 | 0.351 | 8 | 79 |
| Reactome: Antimicrobial peptides | -0.97 | 0.313 | 4 | 45 |
| WP: ATM Signaling Network in Development and Disease | -0.97 | 0.339 | 4 | 45 |
| Reactome: Myogenesis | -0.97 | 0.327 | 2 | 27 |
| Reactome: Insulin processing | -0.97 | 0.328 | 2 | 27 |
| WP: Photodynamic therapy-induced unfolded protein response | -0.97 | 0.366 | 2 | 27 |
| Reactome: Visual phototransduction | -0.98 | 0.315 | 5 | 54 |
| KEGG: Phosphonate and phosphinate metabolism | -0.98 | 0.338 | 0 | 6 |
| Reactome: Synthesis of wybutosine at G37 of tRNA(Phe) | -0.98 | 0.341 | 0 | 6 |
| Reactome: Protein repair | -0.98 | 0.33 | 0 | 6 |
| Reactome: Lipid particle organization | -0.98 | 0.34 | 0 | 6 |
| WP: miRNA Biogenesis | -0.98 | 0.144 | 0 | 6 |
| WP: exRNA mechanism of action and biogenesis | -0.98 | 0.12 | 0 | 6 |
| WP: mir-124 predicted interactions with cell cycle and differentiation | -0.98 | 0.111 | 0 | 6 |
| WP: LncRNA-mediated mechanisms of therapeutic resistance | -0.98 | 0.223 | 0 | 6 |
| WP: Benzene metabolism | -0.98 | 0.273 | 0 | 6 |
| KEGG: GABAergic synapse | -0.99 | 0.317 | 9 | 88 |
| KEGG: Mitophagy | -1 | 0.312 | 6 | 63 |
| WP: Amyotrophic lateral sclerosis (ALS) | -1.02 | 0.346 | 3 | 37 |
| WP: Cell Differentiation - Index expanded | -1.02 | 0.326 | 1 | 18 |
| WP: Inhibition of exosome biogenesis and secretion by Manumycin A in CRPC cells | -1.02 | 0.337 | 1 | 18 |
| WP: Corticotropin-releasing hormone signaling pathway | -1.03 | 0.3 | 9 | 89 |
| KEGG: Phototransduction | -1.03 | 0.305 | 2 | 28 |
| Reactome: Processing of Capped Intronless Pre-mRNA | -1.03 | 0.327 | 2 | 28 |
| Reactome: RHO GTPases activate PKNs | -1.03 | 0.28 | 2 | 28 |
| WP: miRs in Muscle Cell Differentiation | -1.03 | 0.276 | 2 | 28 |
| WP: T-Cell Receptor and Co-stimulatory Signaling | -1.03 | 0.262 | 2 | 28 |
| WP: Nanoparticle-mediated activation of receptor signaling | -1.03 | 0.28 | 2 | 28 |
| Reactome: Transcriptional regulation by RUNX3 | -1.04 | 0.299 | 11 | 106 |
| Reactome: Interleukin-1 family signaling | -1.06 | 0.302 | 15 | 139 |
| KEGG: Thyroid hormone synthesis | -1.06 | 0.292 | 7 | 73 |
| Reactome: Uptake and function of anthrax toxins | -1.06 | 0.281 | 0 | 7 |
| Reactome: TYSND1 cleaves peroxisomal proteins | -1.06 | 0.223 | 0 | 7 |
| WP: Pentose Phosphate Pathway | -1.06 | 0.283 | 0 | 7 |
| WP: DDX1 as a regulatory component of the Drosha microprocessor | -1.06 | 0.12 | 0 | 7 |
| WP: Hfe effect on hepcidin production | -1.06 | 0.134 | 0 | 7 |
| WP: Disorders of the Krebs cycle | -1.06 | 0.329 | 0 | 7 |
| WP: Acetylcholine Synthesis | -1.06 | 0.273 | 0 | 7 |
| WP: Aflatoxin B1 metabolism | -1.06 | 0.222 | 0 | 7 |
| WP: Selenium Metabolism and Selenoproteins | -1.07 | 0.292 | 4 | 47 |
| Reactome: HSP90 chaperone cycle for steroid hormone receptors (SHR) | -1.07 | 0.28 | 3 | 38 |
| KEGG: Sphingolipid signaling pathway | -1.07 | 0.277 | 12 | 115 |
| WP: Signaling Pathways in Glioblastoma | -1.08 | 0.274 | 8 | 82 |
| WP: Apoptosis | -1.08 | 0.307 | 8 | 82 |
| KEGG: Glycosaminoglycan degradation | -1.09 | 0.319 | 1 | 19 |

| | | | | |
|---|---|---|---|---|
| Reactome: RHO GTPases activate CIT | -1.09 | 0.265 | 1 | 19 |
| Reactome: Regulation of TP53 Activity through Methylation | -1.09 | 0.256 | 1 | 19 |
| WP: Mitochondrial Gene Expression | -1.09 | 0.334 | 1 | 19 |
| WP: Urea cycle and metabolism of amino groups | -1.09 | 0.267 | 1 | 19 |
| WP: TNF alpha Signaling Pathway | -1.1 | 0.274 | 9 | 91 |
| Reactome: Kinesins | -1.12 | 0.266 | 3 | 39 |
| Reactome: Signaling by Insulin receptor | -1.12 | 0.264 | 3 | 39 |
| KEGG: T cell receptor signaling pathway | -1.12 | 0.231 | 10 | 100 |
| Reactome: Activation of anterior HOX genes in hindbrain development during early embryogenesis | -1.12 | 0.272 | 10 | 100 |
| KEGG: Sulfur relay system | -1.14 | 0.161 | 0 | 8 |
| Reactome: DNA Damage Reversal | -1.14 | 0.236 | 0 | 8 |
| Reactome: Ubiquinol biosynthesis | -1.14 | 0.282 | 0 | 8 |
| Reactome: Base-Excision Repair, AP Site Formation | -1.14 | 0.243 | 0 | 8 |
| Reactome: TRAIL signaling | -1.14 | 0.222 | 0 | 8 |
| WP: Phase I biotransformations, non P450 | -1.14 | 0.132 | 0 | 8 |
| WP: TFs Regulate miRNAs related to cardiac hypertrophy | -1.14 | 0.142 | 0 | 8 |
| WP: Degradation pathway of sphingolipids, including diseases | -1.14 | 0.257 | 0 | 8 |
| WP: Non-homologous end joining | -1.14 | 0.142 | 0 | 8 |
| KEGG: Citrate cycle (TCA cycle) | -1.14 | 0.277 | 2 | 30 |
| Reactome: Activation of kainate receptors upon glutamate binding | -1.14 | 0.245 | 2 | 30 |
| Reactome: Integration of energy metabolism | -1.15 | 0.242 | 11 | 109 |
| WP: Hematopoietic Stem Cell Gene Regulation by GABP alpha/beta Complex | -1.15 | 0.251 | 1 | 20 |
| KEGG: Apoptosis | -1.15 | 0.267 | 14 | 134 |
| Reactome: Regulation of beta-cell development | -1.16 | 0.258 | 5 | 58 |
| WP: RIG-I-like Receptor Signaling | -1.16 | 0.242 | 5 | 58 |
| WP: MET in type 1 papillary renal cell carcinoma | -1.16 | 0.25 | 5 | 58 |
| Reactome: Mitotic Prometaphase | -1.19 | 0.232 | 19 | 176 |
| Reactome: DNA methylation | -1.2 | 0.237 | 2 | 31 |
| Reactome: Glyoxylate metabolism and glycine degradation | -1.2 | 0.19 | 2 | 31 |
| Reactome: Nucleosome assembly | -1.2 | 0.223 | 4 | 50 |
| Reactome: RHO GTPases Activate Rhotekin and Rhophilins | -1.2 | 0.245 | 0 | 9 |
| WP: NLR Proteins | -1.2 | 0.117 | 0 | 9 |
| WP: Cytosine methylation | -1.2 | 0.165 | 0 | 9 |
| WP: Tgif disruption of Shh signaling | -1.2 | 0.122 | 0 | 9 |
| WP: ATR Signaling | -1.2 | 0.124 | 0 | 9 |
| WP: Globo Sphingolipid Metabolism | -1.21 | 0.24 | 1 | 21 |
| Reactome: SUMOylation of DNA replication proteins | -1.22 | 0.233 | 3 | 41 |
| Reactome: RNA Polymerase III Transcription | -1.22 | 0.228 | 3 | 41 |
| Reactome: SUMOylation of transcription cofactors | -1.22 | 0.223 | 3 | 41 |
| Reactome: Factors involved in megakaryocyte development and platelet production | -1.24 | 0.214 | 9 | 95 |
| Reactome: MHC class II antigen presentation | -1.24 | 0.213 | 9 | 95 |
| KEGG: Glutamatergic synapse | -1.24 | 0.199 | 11 | 112 |
| KEGG: Apoptosis | -1.25 | 0.223 | 2 | 32 |
| Reactome: ATF4 activates genes | -1.25 | 0.196 | 2 | 32 |
| Reactome: Nucleobase catabolism | -1.25 | 0.196 | 2 | 32 |
| Reactome: rRNA modification in the nucleus and cytosol | -1.27 | 0.189 | 1 | 22 |
| WP: Fatty Acid Biosynthesis | -1.27 | 0.25 | 1 | 22 |
| WP: Methionine De Novo and Salvage Pathway | -1.27 | 0.188 | 1 | 22 |
| WP: NAD+ biosynthetic pathways | -1.27 | 0.234 | 1 | 22 |
| Reactome: Mitochondrial iron-sulfur cluster biogenesis | -1.27 | 0.229 | 0 | 10 |
| Reactome: HDR through MMEJ (alt-NHEJ) | -1.27 | 0.213 | 0 | 10 |
| WP: SRF and miRs in Smooth Muscle Differentiation and Proliferation | -1.27 | 0.107 | 0 | 10 |
| WP: Ethanol metabolism resulting in production of ROS by CYP2E1 | -1.27 | 0.203 | 0 | 10 |
| WP: Notch Signaling Pathway | -1.29 | 0.208 | 5 | 61 |
| KEGG: B cell receptor signaling pathway | -1.29 | 0.225 | 6 | 70 |
| Reactome: Mitochondrial biogenesis | -1.29 | 0.196 | 7 | 79 |
| Reactome: DAG and IP3 signaling | -1.3 | 0.179 | 2 | 33 |
| WP: Fatty Acid Beta Oxidation | -1.3 | 0.204 | 2 | 33 |
| WP: Hedgehog Signaling Pathway | -1.31 | 0.194 | 3 | 43 |
| Reactome: TCF dependent signaling in response to WNT | -1.31 | 0.185 | 21 | 197 |
| KEGG: Glycosylphosphatidylinositol (GPI)-anchor biosynthesis | -1.32 | 0.176 | 1 | 23 |
| KEGG: Mismatch repair | -1.32 | 0.131 | 1 | 23 |
| WP: Estrogen signaling pathway | -1.32 | 0.166 | 1 | 23 |
| KEGG: Adherens junction | -1.32 | 0.181 | 6 | 71 |
| Reactome: trans-Golgi Network Vesicle Budding | -1.32 | 0.188 | 6 | 71 |
| Reactome: RHO GTPases activate KTN1 | -1.33 | 0.125 | 0 | 11 |
| WP: PTF1A related regulatory pathway | -1.33 | 0.134 | 0 | 11 |
| WP: Preimplantation Embryo | -1.33 | 0.161 | 4 | 53 |
| KEGG: SNARE interactions in vesicular transport | -1.35 | 0.177 | 2 | 34 |
| WP: Pyrimidine metabolism | -1.37 | 0.162 | 7 | 81 |
| KEGG: RIG-I-like receptor signaling pathway | -1.37 | 0.164 | 5 | 63 |
| Reactome: Cytosolic sensors of pathogen-associated DNA | -1.37 | 0.178 | 5 | 63 |
| Reactome: Peroxisomal lipid metabolism | -1.38 | 0.178 | 1 | 24 |
| Reactome: The citric acid (TCA) cycle and respiratory electron transport | -1.38 | 0.162 | 4 | 54 |
| Reactome: Cytosolic iron-sulfur cluster assembly | -1.39 | 0.156 | 0 | 12 |

| | | | | |
|---|---|---|---|---|
| Reactome: LGI-ADAM interactions | -1.39 | 0.123 | 0 | 12 |
| WP: Iron metabolism in placenta | -1.39 | 0.099 | 0 | 12 |
| WP: Cell Differentiation - Index | -1.39 | 0.152 | 0 | 12 |
| WP: EDA Signalling in Hair Follicle Development | -1.39 | 0.088 | 0 | 12 |
| WP: Kennedy pathway from Sphingolipids | -1.39 | 0.138 | 0 | 12 |
| WP: Purine metabolism | -1.39 | 0.175 | 0 | 12 |
| Reactome: Neutrophil degranulation | -1.39 | 0.147 | 54 | 463 |
| Reactome: RHO GTPases Activate WASPs and WAVEs | -1.4 | 0.168 | 2 | 35 |
| Reactome: Activated PKN1 stimulates transcription of AR (androgen receptor) regulated genes KLK2 and KLK3 | -1.4 | 0.155 | 2 | 35 |
| Reactome: G alpha (z) signalling events | -1.4 | 0.162 | 3 | 45 |
| Reactome: Opioid Signalling | -1.4 | 0.154 | 7 | 82 |
| KEGG: Oocyte meiosis | -1.4 | 0.167 | 11 | 117 |
| Reactome: Amyloid fiber formation | -1.42 | 0.146 | 4 | 55 |
| WP: Signal Transduction of S1P Receptor | -1.43 | 0.092 | 1 | 25 |
| Reactome: COPI-mediated anterograde transport | -1.44 | 0.138 | 7 | 83 |
| KEGG: Cardiac muscle contraction | -1.44 | 0.152 | 6 | 74 |
| KEGG: Hedgehog signaling pathway | -1.44 | 0.143 | 3 | 46 |
| WP: Regulation of Microtubule Cytoskeleton | -1.44 | 0.135 | 3 | 46 |
| WP: Notch Signaling Pathway | -1.44 | 0.148 | 3 | 46 |
| Reactome: Fanconi Anemia Pathway | -1.45 | 0.152 | 2 | 36 |
| KEGG: Necroptosis | -1.45 | 0.151 | 14 | 144 |
| KEGG: Non-homologous end-joining | -1.45 | 0.095 | 0 | 13 |
| WP: Homologous recombination | -1.45 | 0.096 | 0 | 13 |
| WP: DNA IR-Double Strand Breaks (DSBs) and cellular response via ATM | -1.46 | 0.144 | 4 | 56 |
| Reactome: Defensins | -1.48 | 0.143 | 1 | 26 |
| Reactome: Sulfur amino acid metabolism | -1.48 | 0.142 | 1 | 26 |
| Reactome: Glutamate binding, activation of AMPA receptors and synaptic plasticity | -1.48 | 0.134 | 1 | 26 |
| KEGG: Glycosphingolipid biosynthesis | -1.5 | 0.079 | 0 | 14 |
| Reactome: ATF6 (ATF6-alpha) activates chaperone genes | -1.5 | 0.088 | 0 | 14 |
| Reactome: Regulation of cholesterol biosynthesis by SREBP (SREBF) | -1.5 | 0.149 | 0 | 14 |
| Reactome: Mitotic Telophase/Cytokinesis | -1.5 | 0.086 | 0 | 14 |
| KEGG: Long-term potentiation | -1.52 | 0.159 | 5 | 67 |
| Reactome: Signaling by FGFR2 | -1.52 | 0.137 | 5 | 67 |
| WP: Parkin-Ubiquitin Proteasomal System pathway | -1.52 | 0.123 | 5 | 67 |
| Reactome: mTOR signalling | -1.53 | 0.135 | 1 | 27 |
| KEGG: Notch signaling pathway | -1.53 | 0.133 | 3 | 48 |
| KEGG: Dopaminergic synapse | -1.54 | 0.131 | 12 | 130 |
| Reactome: Signaling by TGF-beta Receptor Complex | -1.54 | 0.12 | 2 | 38 |
| KEGG: Carbon metabolism | -1.55 | 0.124 | 10 | 113 |
| Reactome: Unfolded Protein Response (UPR) | -1.56 | 0.122 | 0 | 15 |
| Reactome: Regulated Necrosis | -1.56 | 0.105 | 0 | 15 |
| Reactome: Nucleobase biosynthesis | -1.56 | 0.11 | 0 | 15 |
| Reactome: SUMOylation of DNA methylation proteins | -1.56 | 0.076 | 0 | 15 |
| Reactome: COPII-mediated vesicle transport | -1.56 | 0.121 | 5 | 68 |
| KEGG: N-Glycan biosynthesis | -1.57 | 0.112 | 3 | 49 |
| Reactome: Transcriptional activity of SMAD2/SMAD3:SMAD4 heterotrimer | -1.57 | 0.112 | 3 | 49 |
| WP: IL-3 Signaling Pathway | -1.57 | 0.124 | 3 | 49 |
| KEGG: mRNA surveillance pathway | -1.58 | 0.115 | 7 | 87 |
| Reactome: PRC2 methylates histones and DNA | -1.58 | 0.114 | 2 | 39 |
| WP: Interactome of polycomb repressive complex 2 (PRC2) | -1.61 | 0.084 | 0 | 16 |
| WP: Hedgehog Signaling Pathway | -1.61 | 0.079 | 0 | 16 |
| KEGG: Pyrimidine metabolism | -1.61 | 0.104 | 8 | 97 |
| WP: B Cell Receptor Signaling Pathway | -1.61 | 0.1 | 8 | 97 |
| KEGG: Purine metabolism | -1.61 | 0.104 | 16 | 167 |
| KEGG: Basal transcription factors | -1.63 | 0.117 | 2 | 40 |
| KEGG: RNA polymerase | -1.63 | 0.067 | 1 | 29 |
| Reactome: Endosomal Sorting Complex Required For Transport (ESCRT) | -1.63 | 0.095 | 1 | 29 |
| Reactome: Cristae formation | -1.63 | 0.082 | 1 | 29 |
| Reactome: Class C/3 (Metabotropic glutamate/pheromone receptors) | -1.63 | 0.078 | 1 | 29 |
| WP: MAPK Cascade | -1.63 | 0.058 | 1 | 29 |
| Reactome: Cell Cycle Checkpoints | -1.63 | 0.104 | 28 | 268 |
| WP: DNA IR-damage and cellular response via ATR | -1.65 | 0.09 | 6 | 80 |
| Reactome: TNF signaling | -1.67 | 0.09 | 2 | 41 |
| Reactome: Protein ubiquitination | -1.67 | 0.084 | 1 | 30 |
| Reactome: TCR signaling | -1.7 | 0.088 | 9 | 109 |
| WP: TCA Cycle (aka Krebs or citric acid cycle) | -1.7 | 0.081 | 0 | 18 |
| KEGG: Autophagy | -1.71 | 0.084 | 11 | 127 |
| Reactome: ERCC6 (CSB) and EHMT2 (G9a) positively regulate rRNA expression | -1.71 | 0.076 | 2 | 42 |
| WP: Metabolic reprogramming in colon cancer | -1.71 | 0.067 | 2 | 42 |
| WP: DNA Replication | -1.71 | 0.079 | 2 | 42 |
| KEGG: Autophagy | -1.72 | 0.07 | 1 | 31 |
| WP: Tumor suppressor activity of SMARCB1 | -1.72 | 0.059 | 1 | 31 |
| Reactome: Semaphorin interactions | -1.73 | 0.067 | 4 | 63 |
| KEGG: Metabolic pathways | -1.73 | 0.094 | 152 | 1243 |

| | | | | |
|---|---|---|---|---|
| Reactome: MAPK6/MAPK4 signaling | -1.74 | 0.086 | 7 | 92 |
| Reactome: Regulation of TP53 Activity through Phosphorylation | -1.74 | 0.079 | 7 | 92 |
| KEGG: Retrograde endocannabinoid signaling | -1.74 | 0.075 | 13 | 146 |
| Reactome: RHO GTPases Activate ROCKs | -1.75 | 0.05 | 0 | 19 |
| Reactome: Listeria monocytogenes entry into host cells | -1.75 | 0.069 | 0 | 19 |
| Reactome: TBC/RABGAPs | -1.75 | 0.073 | 2 | 43 |
| KEGG: Peroxisome | -1.76 | 0.081 | 6 | 83 |
| Reactome: Glucose metabolism | -1.76 | 0.054 | 6 | 83 |
| Reactome: Regulation of mitotic cell cycle | -1.76 | 0.067 | 6 | 83 |
| Reactome: Host Interactions with Influenza Factors | -1.76 | 0.079 | 1 | 32 |
| WP: Initiation of transcription and translation elongation at the HIV-1 LTR | -1.76 | 0.054 | 1 | 32 |
| Reactome: Mitotic Metaphase and Anaphase | -1.77 | 0.072 | 16 | 173 |
| Reactome: E3 ubiquitin ligases ubiquitinate target proteins | -1.77 | 0.075 | 3 | 54 |
| Reactome: Protein folding | -1.78 | 0.064 | 7 | 93 |
| KEGG: Vasopressin-regulated water reabsorption | -1.79 | 0.047 | 2 | 44 |
| Reactome: Transcriptional Regulation by E2F6 | -1.79 | 0.082 | 2 | 44 |
| Reactome: POU5F1 (OCT4), SOX2, NANOG activate genes related to proliferation | -1.8 | 0.059 | 0 | 20 |
| WP: Nanomaterial induced apoptosis | -1.8 | 0.055 | 0 | 20 |
| WP: Glycerophospholipid Biosynthetic Pathway | -1.8 | 0.06 | 0 | 20 |
| WP: Apoptosis Modulation by HSP70 | -1.8 | 0.049 | 0 | 20 |
| WP: Nonalcoholic fatty liver disease | -1.8 | 0.068 | 13 | 148 |
| KEGG: Base excision repair | -1.8 | 0.064 | 1 | 33 |
| KEGG: Drug metabolism | -1.8 | 0.08 | 4 | 65 |
| Reactome: Meiotic synapsis | -1.81 | 0.058 | 3 | 55 |
| Reactome: RHO GTPases activate PAKs | -1.84 | 0.059 | 0 | 21 |
| Reactome: Beta-catenin independent WNT signaling | -1.85 | 0.057 | 12 | 141 |
| Reactome: Macroautophagy | -1.88 | 0.057 | 4 | 67 |
| Reactome: Iron uptake and transport | -1.88 | 0.062 | 3 | 57 |
| KEGG: Mannose type O-glycan biosynthesis | -1.88 | 0.055 | 0 | 22 |
| Reactome: Mitochondrial Fatty Acid Beta-Oxidation | -1.89 | 0.059 | 1 | 35 |
| KEGG: Protein export | -1.93 | 0.057 | 0 | 23 |
| WP: Nanoparticle triggered autophagic cell death | -1.93 | 0.062 | 0 | 23 |
| Reactome: DNA Damage/Telomere Stress Induced Senescence | -1.96 | 0.038 | 3 | 59 |
| Reactome: Peroxisomal protein import | -1.96 | 0.052 | 3 | 59 |
| KEGG: Biosynthesis of amino acids | -1.98 | 0.035 | 4 | 70 |
| Reactome: Regulation of RUNX2 expression and activity | -1.98 | 0.055 | 4 | 70 |
| WP: G Protein Signaling Pathways | -1.98 | 0.037 | 6 | 90 |
| WP: G13 Signaling Pathway | -2.01 | 0.025 | 1 | 38 |
| Reactome: Mitophagy | -2.01 | 0.03 | 0 | 25 |
| WP: MTHFR deficiency | -2.01 | 0.035 | 0 | 25 |
| Reactome: NoRC negatively regulates rRNA expression | -2.01 | 0.036 | 4 | 71 |
| KEGG: Human immunodeficiency virus 1 infection | -2.03 | 0.043 | 18 | 201 |
| WP: Eukaryotic Transcription Initiation | -2.05 | 0.028 | 1 | 39 |
| KEGG: Lysosome | -2.05 | 0.039 | 9 | 121 |
| Reactome: RNA Polymerase I Transcription | -2.06 | 0.041 | 3 | 62 |
| Reactome: Regulation of DNA replication | -2.08 | 0.039 | 4 | 73 |
| Reactome: TP53 Regulates Transcription of Cell Death Genes | -2.08 | 0.041 | 4 | 73 |
| KEGG: Homologous recombination | -2.12 | 0.031 | 1 | 41 |
| WP: Common Pathways Underlying Drug Addiction | -2.12 | 0.023 | 1 | 41 |
| WP: Fas Ligand (FasL) pathway and Stress induction of Heat Shock Proteins (HSP) regulation | -2.12 | 0.025 | 1 | 41 |
| Reactome: mRNA Capping | -2.13 | 0.03 | 0 | 28 |
| Reactome: G-protein beta:gamma signalling | -2.13 | 0.028 | 0 | 28 |
| Reactome: Meiotic recombination | -2.13 | 0.031 | 2 | 53 |
| KEGG: Endocytosis | -2.14 | 0.029 | 22 | 241 |
| Reactome: Transcriptional regulation by RUNX1 | -2.16 | 0.026 | 14 | 171 |
| KEGG: Nucleotide excision repair | -2.19 | 0.023 | 1 | 43 |
| Reactome: Deadenylation-dependent mRNA decay | -2.2 | 0.024 | 2 | 55 |
| Reactome: Regulation of TP53 Activity through Acetylation | -2.2 | 0.022 | 0 | 30 |
| Reactome: SUMOylation of SUMOylation proteins | -2.2 | 0.025 | 0 | 30 |
| Reactome: S Phase | -2.23 | 0.028 | 6 | 98 |
| Reactome: Oxidative Stress Induced Senescence | -2.23 | 0.026 | 6 | 98 |
| Reactome: Vasopressin regulates renal water homeostasis via Aquaporins | -2.23 | 0.018 | 1 | 44 |
| Reactome: B-WICH complex positively regulates rRNA expression | -2.23 | 0.023 | 2 | 56 |
| Reactome: ABC-family proteins mediated transport | -2.25 | 0.019 | 6 | 99 |
| Reactome: Intra-Golgi and retrograde Golgi-to-ER traffic | -2.26 | 0.028 | 14 | 175 |
| WP: Androgen receptor signaling pathway | -2.26 | 0.019 | 5 | 89 |
| Reactome: TNFR2 non-canonical NF-kB pathway | -2.27 | 0.029 | 2 | 57 |
| Reactome: TP53 Regulates Metabolic Genes | -2.29 | 0.019 | 5 | 90 |
| Reactome: SUMOylation of ubiquitinylation proteins | -2.31 | 0.017 | 0 | 33 |
| Reactome: DNA Double Strand Break Response | -2.33 | 0.022 | 2 | 59 |
| WP: Proteasome Degradation | -2.33 | 0.013 | 2 | 59 |
| Reactome: tRNA modification in the nucleus and cytosol | -2.38 | 0.015 | 0 | 35 |
| Reactome: Degradation of beta-catenin by the destruction complex | -2.39 | 0.015 | 4 | 83 |
| KEGG: Fanconi anemia pathway | -2.4 | 0.017 | 1 | 49 |
| KEGG: DNA replication | -2.41 | 0.016 | 0 | 36 |

| | | | | |
|---|---|---|---|---|
| Reactome: Resolution of Abasic Sites (AP sites) | -2.41 | 0.02 | 0 | 36 |
| Reactome: Regulation of Apoptosis | -2.43 | 0.016 | 1 | 50 |
| KEGG: Ribosome biogenesis in eukaryotes | -2.45 | 0.007 | 3 | 74 |
| Reactome: Nonhomologous End-Joining (NHEJ) | -2.46 | 0.015 | 1 | 51 |
| Reactome: tRNA processing in the nucleus | -2.5 | 0.008 | 1 | 52 |
| KEGG: RNA degradation | -2.51 | 0.011 | 3 | 76 |
| Reactome: RAB GEFs exchange GTP for GDP on RABs | -2.51 | 0.014 | 4 | 87 |
| Reactome: tRNA Aminoacylation | -2.57 | 0.004 | 0 | 41 |
| Reactome: SUMOylation of RNA binding proteins | -2.57 | 0.012 | 0 | 41 |
| KEGG: Proteasome | -2.6 | 0.018 | 0 | 42 |
| Reactome: C-type lectin receptors (CLRs) | -2.61 | 0.006 | 8 | 132 |
| Reactome: Neddylation | -2.61 | 0.015 | 18 | 227 |
| Reactome: NIK-->noncanonical NF-kB signaling | -2.62 | 0.013 | 1 | 56 |
| KEGG: Aminoacyl-tRNA biosynthesis | -2.63 | 0.011 | 0 | 43 |
| Reactome: M/G1 Transition | -2.68 | 0.01 | 3 | 82 |
| Reactome: DNA Replication Pre-Initiation | -2.68 | 0.01 | 3 | 82 |
| Reactome: Fc epsilon receptor (FCERI) signaling | -2.68 | 0.004 | 7 | 125 |
| WP: Cytoplasmic Ribosomal Proteins | -2.74 | 0.005 | 3 | 84 |
| Reactome: Signaling by NOTCH4 | -2.77 | 0.007 | 3 | 85 |
| Reactome: Intrinsic Pathway for Apoptosis | -2.78 | 0.009 | 0 | 48 |
| Reactome: Metabolism of non-coding RNA | -2.78 | 0.005 | 0 | 48 |
| Reactome: XBP1(S) activates chaperone genes | -2.79 | 0.008 | 4 | 97 |
| Reactome: Hedgehog ligand biogenesis | -2.8 | 0.001 | 1 | 62 |
| WP: Translation Factors | -2.81 | 0.012 | 0 | 49 |
| Reactome: Hedgehog 'on' state | -2.82 | 0.001 | 3 | 87 |
| KEGG: Protein processing in endoplasmic reticulum | -2.85 | 0.007 | 10 | 162 |
| Reactome: HDR through Homologous Recombination (HRR) or Single Strand Annealing (SSA) | -2.86 | 0.004 | 5 | 111 |
| Reactome: SUMOylation of chromatin organization proteins | -2.87 | 0.004 | 0 | 51 |
| KEGG: Ubiquitin mediated proteolysis | -2.95 | 0.006 | 7 | 136 |
| WP: Mitochondrial complex I assembly model OXPHOS system | -2.98 | 0.004 | 0 | 55 |
| Reactome: Synthesis of DNA | -2.99 | 0.003 | 5 | 116 |
| Reactome: SUMOylation of DNA damage response and repair proteins | -3.02 | 0.001 | 1 | 70 |
| Reactome: Mitotic G2-G2/M phases | -3.03 | 0.005 | 11 | 180 |
| Reactome: Mitotic Prophase | -3.03 | 0.004 | 3 | 95 |
| Reactome: Cellular response to heat stress | -3.05 | 0.003 | 3 | 96 |
| WP: Oxidative phosphorylation | -3.06 | 0.004 | 0 | 58 |
| Reactome: Telomere Maintenance | -3.11 | 0.001 | 0 | 60 |
| Reactome: RNA polymerase II transcribes snRNA genes | -3.12 | 0.005 | 1 | 74 |
| Reactome: TP53 Regulates Transcription of DNA Repair Genes | -3.15 | 0.003 | 1 | 75 |
| Reactome: Mitochondrial protein import | -3.17 | 0.004 | 0 | 62 |
| Reactome: Hedgehog 'off' state | -3.18 | 0.003 | 3 | 101 |
| Reactome: Selenoamino acid metabolism | -3.21 | 0.001 | 4 | 114 |
| Reactome: Deubiquitination | -3.23 | 0 | 16 | 239 |
| KEGG: Thermogenesis | -3.26 | 0.004 | 14 | 221 |
| Reactome: Signaling by the B Cell Receptor (BCR) | -3.27 | 0 | 3 | 105 |
| Reactome: Assembly of the primary cilium | -3.29 | 0 | 10 | 182 |
| Reactome: Cilium Assembly | -3.29 | 0 | 10 | 182 |
| Reactome: Metabolism of polyamines | -3.3 | 0.001 | 1 | 81 |
| Reactome: Nucleotide Excision Repair | -3.37 | 0.003 | 3 | 109 |
| Reactome: PTEN Regulation | -3.39 | 0.001 | 5 | 133 |
| Reactome: Asparagine N-linked glycosylation | -3.4 | 0 | 4 | 122 |
| WP: mRNA Processing | -3.4 | 0 | 4 | 122 |
| Reactome: Gene Silencing by RNA | -3.4 | 0.002 | 2 | 98 |
| KEGG: Spliceosome | -3.51 | 0 | 4 | 127 |
| Reactome: Signaling by ROBO receptors | -3.52 | 0.001 | 12 | 214 |
| Reactome: Eukaryotic Translation Elongation | -3.53 | 0.001 | 1 | 91 |
| Reactome: Eukaryotic Translation Termination | -3.53 | 0.001 | 1 | 91 |
| Reactome: Mitochondrial translation | -3.53 | 0 | 1 | 91 |
| Reactome: Regulation of mRNA stability by proteins that bind AU-rich elements | -3.64 | 0 | 0 | 82 |
| Reactome: Nonsense-Mediated Decay (NMD) | -3.73 | 0.001 | 2 | 113 |
| KEGG: RNA transport | -3.81 | 0.001 | 5 | 153 |
| Reactome: Class I MHC mediated antigen processing & presentation | -3.84 | 0 | 25 | 360 |
| Reactome: RNA Polymerase II Transcription | -3.89 | 0.001 | 4 | 145 |
| Reactome: SRP-dependent cotranslational protein targeting to membrane | -3.94 | 0 | 1 | 110 |
| KEGG: Oxidative phosphorylation | -3.96 | 0.001 | 2 | 124 |
| WP: Electron Transport Chain (OXPHOS system in mitochondria) | -3.98 | 0 | 0 | 98 |
| KEGG: Ribosome | -4.02 | 0 | 2 | 127 |
| Reactome: Eukaryotic Translation Initiation | -4.09 | 0 | 1 | 117 |
| Reactome: Host Interactions of HIV factors | -4.14 | 0.001 | 1 | 120 |
| Reactome: Chromatin organization | -4.26 | 0 | 9 | 222 |
| Reactome: Respiratory electron transport, ATP synthesis by chemiosmotic coupling, and heat production by uncoupling proteins. | -4.39 | 0 | 0 | 119 |
| Reactome: Influenza Life Cycle | -4.47 | 0 | 1 | 137 |
| Reactome: HIV Life Cycle | -4.54 | 0 | 1 | 141 |
| Reactome: Major pathway of rRNA processing in the nucleolus and cytosol | -4.57 | 0 | 2 | 156 |

| | | | | |
|---|---|---|---|---|
| Reactome: Generic Transcription Pathway | -5.02 | 0 | 19 | 378 |
| Reactome: Processing of Capped Intron-Containing Pre-mRNA | -5.03 | 0 | 6 | 234 |
| WP: Human metabolism overview | NaN | 0 | 0 | 0 |
| WP: Biochemical Pathways Part I | NaN | 0 | 0 | 0 |
| WP: Biosynthesis and regeneration of tetrahydrobiopterin (BH4) and catabolism of phenylalanine, including diseases | NaN | 0 | 0 | 0 |
| WP: GHB metabolic pathway | NaN | 0 | 0 | 0 |
| WP: Mevalonate arm of cholesterol biosynthesis pathway | NaN | 0 | 0 | 0 |
| WP: Phosphatidylcholine catabolism | NaN | 0 | 0 | 0 |
| WP: Amino acid conjugation | NaN | 0 | 0 | 0 |

**Positive: the number of genes on the pathway that pass the statistical criteria (absolute logFC > 0.58 and p-value < 0.05)*
**Measured: the number of genes on the pathway that were measured in the dataset*
*WP: WikiPathways*
*The red line indicates the end of the significantly changed pathways for this analysis*
