# Supplementary material for: The Molecular Processes in the Trabecular Meshwork After Exposure to Corticosteroids and in Corticosteroid-Induced Ocular Hypertension
Source: Invest Ophthalmol Vis Sci. 2020 Apr 18;61(4):24. doi: 10.1167/iovs.61.4.24 (PMC7401422; doi:10.1167/iovs.61.4.24)
Supplement: Supplement 3 [file iovs-61-4-24_s003.pdf]

Appendix 3. Complete pathway results of effect of dexamethasone on TM: bovine data

Pathway	Z Score	Permuted p-value	Positive*	Measured*
Reactome: Collagen chain trimerization	9,44	<0,0001	21	39
WP: miRNA targets in ECM and membrane receptors	8,18	<0,0001	13	21
Reactome: Interleukin-4 and Interleukin-13 signaling	8,1	<0,0001	52	197
KEGG: Protein digestion and absorption	7,99	<0,0001	29	82
Reactome: Collagen biosynthesis and modifying enzymes	7,94	<0,0001	24	61
WP: Photodynamic therapy-induced HIF-1 survival signaling	6,99	<0,0001	16	37
WP: Glycolysis and Gluconeogenesis	6,71	<0,0001	17	43
WP: Cori Cycle	6,37	<0,0001	9	16
Reactome: Binding and Uptake of Ligands by Scavenger Receptors	6,01	<0,0001	14	36
WP: Differentiation Pathway	5,84	<0,0001	16	46
Reactome: Interferon alpha/beta signaling	5,76	<0,0001	20	66
Reactome: Assembly of collagen fibrils and other multimeric structures	5,67	<0,0001	15	43
Reactome: Regulation of Insulin-like Growth Factor (IGF) transport and uptake by Insulin-like Growth Factor Binding Proteins (IGFBPs)	5,48	<0,0001	28	114
WP: Metabolic reprogramming in colon cancer	5,37	<0,0001	14	41
WP: Lung fibrosis	5,23	<0,0001	17	57
WP: Nuclear Receptors Meta-Pathway	5,2	<0,0001	53	287
WP: LncRNA involvement in canonical Wnt signaling and colorectal cancer	5,07	<0,0001	23	92
WP: ncRNAs involved in Wnt signaling in hepatocellular carcinoma	5,04	<0,0001	21	81
WP: IL1 and megakaryocytes in obesity	4,84	<0,0001	9	23
KEGG: Glycolysis / Gluconeogenesis	4,73	<0,0001	17	63
WP: Spinal Cord Injury	4,68	<0,0001	25	111
WP: Melatonin metabolism and effects	4,66	<0,0001	11	33
Reactome: Post-translational protein phosphorylation	4,6	<0,0001	23	100
KEGG: Biosynthesis of amino acids	4,55	<0,0001	18	71
Reactome: Extracellular matrix organization	4,47	<0,0001	19	78
WP: Pathways in clear cell renal cell carcinoma	4,47	<0,0001	20	84
WP: Amplification and Expansion of Oncogenic Pathways as Metastatic Traits	4,45	0,001	7	17
KEGG: HIF-1 signaling pathway	4,42	<0,0001	22	97
Reactome: Elastic fibre formation	4,31	0,001	12	41
WP: HIF1A and PPARG regulation of glycolysis	4,29	<0,0001	4	7
KEGG: TNF signaling pathway	4,28	<0,0001	23	106
WP: Matrix Metalloproteinases	4,22	0,001	9	27
WP: Oxidative Stress	4,22	0,002	9	27
Reactome: Interleukin-10 signaling	4,21	0,001	18	76
KEGG: ECM-receptor interaction	4,15	<0,0001	18	77
WP: Prostaglandin Synthesis and Regulation	4,11	<0,0001	12	43
WP: Photodynamic therapy-induced NF-kB survival signaling	4,07	<0,0001	10	33
WP: Angiogenesis	3,97	0,003	8	24
Reactome: ATF4 activates genes	3,95	0,002	9	29
WP: Wnt Signaling Pathway	3,93	0,001	23	113
KEGG: Focal adhesion	3,88	<0,0001	34	192
WP: Focal Adhesion	3,88	<0,0001	34	192
WP: Adipogenesis	3,87	0,001	25	128
Reactome: Metallothioneins bind metals	3,84	<0,0001	3	5
WP: Glucocorticoid and Mineralcorticoid Metabolism	3,84	0,001	3	5
Reactome: Glycosaminoglycan metabolism	3,74	<0,0001	23	117
WP: Sleep regulation	3,63	0,001	10	37
Reactome: Transport of inorganic cations/anions and amino acids/oligopeptides	3,48	<0,0001	20	102
KEGG: Complement and coagulation cascades	3,43	0,001	16	76
WP: Viral Acute Myocarditis	3,41	0,003	17	83
WP: Vitamin D Receptor Pathway	3,32	0,004	27	156
WP: Platelet-mediated interactions with vascular and circulating cells	3,28	0,003	6	19
WP: PI3K-Akt Signaling Pathway	3,24	0,002	47	317
KEGG: PI3K-Akt signaling pathway	3,15	0,002	48	329
Reactome: Regulation of TLR by endogenous ligand	3,14	0,006	5	15
KEGG: TGF-beta signaling pathway	3,14	0,002	16	81
KEGG: Other types of O-glycan biosynthesis	3,12	0,008	6	20
WP: Selenium Micronutrient Network	3,11	0,002	14	68
WP: Ectoderm Differentiation	3,1	0,002	23	132
WP: Endothelin Pathways	3,09	0,004	8	31
WP: Influenza A virus infection	3,08	0,009	1	1
WP: Colchicine Metabolic Pathway	3,08	0,005	1	1
WP: Metabolism of Tetrahydrocannabinol (THC)	3,08	0,011	1	1
WP: Epithelial to mesenchymal transition in colorectal cancer	3,05	0,001	26	156
KEGG: Wnt signaling pathway	3,04	0,002	24	141
WP: Complement and Coagulation Cascades	3,04	0,003	12	56
Reactome: Peptide hormone biosynthesis	3,03	0,007	4	11
WP: Cardiac Progenitor Differentiation	3,01	0,002	11	50
WP: Benzo(a)pyrene metabolism	3	0,007	3	7
WP: Focal Adhesion-PI3K-Akt-mTOR-signaling pathway	2,98	0,002	43	295

WP: Complement Activation	2,97	0,012	6	21
KEGG: FoxO signaling pathway	2,96	0,004	22	128
WP: Pathogenic Escherichia coli infection	2,93	0,007	11	51
KEGG: Hippo signaling pathway	2,9	0,008	7	27
WP: Photodynamic therapy-induced unfolded protein response	2,9	0,012	7	27
KEGG: Glycine, serine and threonine metabolism	2,88	0,007	9	39
KEGG: Cytokine-cytokine receptor interaction	2,88	0,005	37	249
WP: Chromosomal and microsatellite instability in colorectal cancer	2,87	0,003	14	72
WP: Apoptosis Modulation and Signaling	2,82	0,005	16	87
KEGG: Relaxin signaling pathway	2,82	0,003	21	124
Reactome: Cell surface interactions at the vascular wall	2,82	0,008	19	109
Reactome: Glucose metabolism	2,82	0,006	15	80
Reactome: Biosynthesis of DHA-derived SPMs	2,81	0,008	4	12
WP: Transcriptional cascade regulating adipogenesis	2,81	0,008	4	12
Reactome: BMAL1:CLOCK,NPAS2 activates circadian gene expression	2,78	0,011	8	34
KEGG: Axon guidance	2,77	0,005	27	172
KEGG: p53 signaling pathway	2,76	0,011	13	67
Reactome: Neurotransmitter uptake and metabolism In glial cells	2,76	0,015	2	4
Reactome: Biosynthesis of DPA-derived SPMs	2,76	0,006	2	4
WP: Cocaine metabolism	2,76	0,008	2	4
WP: Copper homeostasis	2,75	0,005	10	47
Reactome: Signaling by Retinoic Acid	2,71	0,008	9	41
WP: Vitamin A and Carotenoid Metabolism	2,71	0,007	9	41
WP: Apoptosis	2,71	0,012	15	82
WP: Trans-sulfuration and one carbon metabolism	2,68	0,008	7	29
WP: NRF2 pathway	2,66	0,005	21	128
KEGG: IL-17 signaling pathway	2,66	0,011	15	83
WP: Overview of nanoparticle effects	2,64	0,013	5	18
WP: TGF-B Signaling in Thyroid Cells for Epithelial-Mesenchymal Transition	2,64	0,025	5	18
KEGG: Inflammatory mediator regulation of TRP channels	2,62	0,008	16	91
Reactome: Dissolution of Fibrin Clot	2,61	0,011	4	13
WP: BMP2-WNT4-FOXO1 Pathway in Human Primary Endometrial Stromal Cell Differentiation	2,61	0,036	4	13
KEGG: Hippo signaling pathway	2,61	0,005	23	145
Reactome: Collagen degradation	2,6	0,009	8	36
KEGG: Arachidonic acid metabolism	2,58	0,013	11	56
WP: Endometrial cancer	2,58	0,013	12	63
Reactome: O-linked glycosylation	2,53	0,013	16	93
WP: Photodynamic therapy-induced AP-1 survival signaling.	2,53	0,019	10	50
WP: TP53 Network	2,49	0,017	5	19
KEGG: Fructose and mannose metabolism	2,48	0,015	7	31
KEGG: Mineral absorption	2,47	0,012	9	44
WP: Exercise-induced Circadian Regulation	2,47	0,013	9	44
Reactome: Deregulated CDK5 triggers multiple neurodegenerative pathways in Alzheimer's disease models	2,47	0,016	6	25
Reactome: Serine biosynthesis	2,43	0,021	3	9
WP: Heme Biosynthesis	2,43	0,015	3	9
Reactome: ATF6 (ATF6-alpha) activates chaperone genes	2,43	0,03	4	14
WP: Osteoblast Signaling	2,43	0,027	4	14
WP: Estrogen metabolism	2,43	0,014	4	14
Reactome: Neutrophil degranulation	2,4	0,009	55	426
KEGG: Ovarian steroidogenesis	2,4	0,02	9	45
Reactome: Arachidonic acid metabolism	2,39	0,014	10	52
WP: TGF-beta Receptor Signaling	2,39	0,02	10	52
WP: DNA Damage Response (only ATM dependent)	2,37	0,011	18	112
KEGG: Gastric cancer	2,36	0,011	22	144
Reactome: Syndecan interactions	2,36	0,016	5	20
WP: Imatinib and Chronic Myeloid Leukemia	2,36	0,031	5	20
WP: Hypertrophy Model	2,36	0,033	5	20
KEGG: Neomycin, kanamycin and gentamicin biosynthesis	2,32	0,022	2	5
WP: Endochondral Ossification	2,27	0,02	11	61
KEGG: JAK-STAT signaling pathway	2,25	0,029	21	139
Reactome: Activation of Matrix Metalloproteinases	2,25	0,02	6	27
WP: Transcription factor regulation in adipogenesis	2,23	0,026	5	21
WP: Primary Focal Segmental Glomerulosclerosis FSGS	2,23	0,015	12	69
Reactome: Detoxification of Reactive Oxygen Species	2,2	0,025	7	34
WP: Folate Metabolism	2,15	0,036	11	63
KEGG: NF-kappa B signaling pathway	2,14	0,027	14	86
KEGG: Carbon metabolism	2,12	0,023	17	110
WP: Wnt Signaling in Kidney Disease	2,11	0,036	7	35
WP: Vitamin B12 Metabolism	2,11	0,036	9	49
WP: Regulation of Apoptosis by Parathyroid Hormone-related Protein	2,11	0,035	5	22
WP: The human immune response to tuberculosis	2,11	0,038	5	22
KEGG: Phenylalanine metabolism	2,11	0,02	4	16
Reactome: Metabolism of porphyrins	2,11	0,034	4	16
WP: Deregulation of Rab and Rab Effector Genes in Bladder Cancer	2,11	0,034	4	16

KEGG: Apoptosis	2,09	0,038	19	127
KEGG: Apoptosis	2,05	0,045	6	29
WP: Apoptosis-related network due to altered Notch3 in ovarian cancer	2,04	0,032	9	50
WP: Phosphodiesterases in neuronal function	2,04	0,039	9	50
KEGG: Cysteine and methionine metabolism	2,03	0,03	8	43
Reactome: Gamma carboxylation, hypusine formation and arylsulfatase activation	2,03	0,047	7	36
Reactome: RET signaling	2,03	0,048	7	36
Reactome: Phase II - Conjugation of compounds	2	0,03	13	81
Reactome: Response to elevated platelet cytosolic Ca2+	2	0,042	17	113
WP: Eicosanoid Synthesis	2	0,034	5	23
WP: Cytokines and Inflammatory Response	2	0,037	5	23
KEGG: Vitamin B6 metabolism	1,99	0,043	2	6
Reactome: Uptake and function of diphtheria toxin	1,99	0,037	2	6
WP: Metastatic brain tumor	1,99	0,046	2	6
WP: miR-509-3p alteration of YAP1/ECM axis	1,97	0,054	4	17
KEGG: Cholesterol metabolism	1,96	0,047	8	44
Reactome: Retinoid metabolism and transport	1,96	0,051	6	30
WP: Extracellular vesicle-mediated signaling in recipient cells	1,96	0,048	6	30
WP: miRNA regulation of prostate cancer signaling pathways	1,96	0,042	6	30
WP: Amino Acid metabolism	1,95	0,048	14	90
KEGG: Caffeine metabolism	1,95	0,052	1	2
Reactome: Transport of glycerol from adipocytes to the liver by Aquaporins	1,95	0,038	1	2
Reactome: Biosynthesis of electrophilic Ï‰-3 PUFA oxo-derivatives	1,95	0,055	1	2
WP: Lidocaine metabolism	1,95	0,049	1	2
WP: Felbamate Metabolism	1,95	0,04	1	2
WP: Caffeine and Theobromine metabolism	1,95	0,063	1	2
Reactome: NCAM signaling for neurite out-growth	1,95	0,042	7	37
WP: TYROBP Causal Network	1,94	0,044	10	59
WP: VEGFA-VEGFR2 Signaling Pathway	1,93	0,054	30	226
Reactome: Interleukin-6 family signaling	1,89	0,045	5	24
Reactome: Transcriptional regulation by RUNX2	1,89	0,047	10	60
Reactome: DAG and IP3 signaling	1,86	0,045	6	31
WP: Type 2 papillary renal cell carcinoma	1,86	0,051	6	31
WP: Type II interferon signaling (IFNG)	1,86	0,04	6	31
Reactome: Integrin cell surface interactions	1,83	0,067	10	61
Reactome: Semaphorin interactions	1,83	0,067	10	61
Reactome: Passive transport by Aquaporins	1,83	0,05	3	12
WP: Iron metabolism in placenta	1,83	0,072	3	12
KEGG: Arginine and proline metabolism	1,82	0,066	8	46
KEGG: Hematopoietic cell lineage	1,81	0,07	12	77
Reactome: WNT ligand biogenesis and trafficking	1,78	0,065	5	25
Reactome: Nicotinate metabolism	1,78	0,069	5	25
Reactome: Post-translational modification: synthesis of GPI-anchored proteins	1,77	0,099	12	78
WP: Parkin-Ubiquitin Proteasomal System pathway	1,72	0,072	10	63
Reactome: TRAIL signaling	1,72	0,052	2	7
WP: LncRNA-mediated mechanisms of therapeutic resistance	1,72	0,08	2	7
Reactome: Degradation of the extracellular matrix	1,71	0,1	11	71
Reactome: Amino acid synthesis and interconversion (transamination)	1,71	0,067	4	19
WP: Small Ligand GPCRs	1,71	0,081	4	19
WP: Urea cycle and metabolism of amino groups	1,71	0,065	4	19
Reactome: Signaling by PDGF	1,69	0,074	6	33
WP: Genes targeted by miRNAs in adipocytes	1,66	0,085	3	13
WP: EDA Signalling in Hair Follicle Development	1,66	0,077	3	13
WP: ncRNAs involved in STAT3 signaling in hepatocellular carcinoma	1,66	0,068	3	13
WP: Tamoxifen metabolism	1,66	0,059	3	13
WP: Wnt Signaling Pathway and Pluripotency	1,65	0,093	14	97
Reactome: tRNA Aminoacylation	1,65	0,09	7	41
Reactome: Class B/2 (Secretin family receptors)	1,64	0,102	13	89
KEGG: Glutathione metabolism	1,62	0,087	8	49
Reactome: Complement cascade	1,62	0,111	8	49
Reactome: Netrin-1 signaling	1,62	0,114	8	49
KEGG: Hepatocellular carcinoma	1,62	0,088	21	158
Reactome: Class A/1 (Rhodopsin-like receptors)	1,62	0,098	35	284
Reactome: Smooth Muscle Contraction	1,61	0,082	6	34
WP: Brain-Derived Neurotrophic Factor (BDNF) signaling pathway	1,6	0,122	19	141
KEGG: MAPK signaling pathway	1,59	0,129	35	285
WP: Cannabinoid receptor signaling	1,59	0,074	5	27
WP: Statin Pathway	1,59	0,096	5	27
Reactome: G alpha (q) signalling events	1,59	0,106	21	159
WP: One carbon metabolism and related pathways	1,56	0,103	8	50
WP: Ebola Virus Pathway on Host	1,55	0,115	15	108
KEGG: Gap junction	1,53	0,118	12	83
Reactome: Caspase activation via Death Receptors in the presence of ligand	1,52	0,09	3	14
WP: Phytochemical activity on NRF2 transcriptional activation	1,52	0,078	3	14

WP: Glucuronidation	1,52	0,069	3	14
WP: IL-6 signaling pathway	1,51	0,094	7	43
WP: PPAR signaling pathway	1,51	0,121	10	67
WP: Phase I biotransformations, non P450	1,49	0,089	2	8
WP: Caloric restriction and aging	1,49	0,083	2	8
Reactome: RAF-independent MAPK1/3 activation	1,49	0,12	4	21
Reactome: RHO GTPases activate PAKs	1,49	0,137	4	21
Reactome: Nucleotide salvage	1,49	0,134	4	21
Reactome: Signaling by NTRK2 (TRKB)	1,49	0,12	4	21
WP: Senescence and Autophagy in Cancer	1,45	0,145	14	102
WP: IL-4 Signaling Pathway	1,44	0,141	8	52
KEGG: Cellular senescence	1,44	0,164	19	146
Reactome: Nucleobase catabolism	1,42	0,165	5	29
WP: Inflammatory Response Pathway	1,42	0,088	5	29
WP: Circadian rythm related genes	1,41	0,171	24	192
KEGG: Prolactin signaling pathway	1,41	0,175	10	69
Reactome: Intestinal absorption	1,4	0,088	1	3
Reactome: Lactose synthesis	1,4	0,09	1	3
WP: Heroin metabolism	1,4	0,082	1	3
WP: Arylamine metabolism	1,4	0,088	1	3
Reactome: Formation of Fibrin Clot (Clotting Cascade)	1,39	0,176	6	37
WP: Amyotrophic lateral sclerosis (ALS)	1,39	0,173	6	37
KEGG: Proximal tubule bicarbonate reclamation	1,38	0,108	4	22
WP: Methionine De Novo and Salvage Pathway	1,38	0,176	4	22
WP: Human Complement System	1,38	0,179	13	95
KEGG: Regulation of lipolysis in adipocytes	1,38	0,201	8	53
Reactome: Signaling by Type 1 Insulin-like Growth Factor 1 Receptor (IGF1R)	1,38	0,166	3	15
WP: miRNAs involved in DNA damage response	1,38	0,176	3	15
WP: H19 action Rb-E2F1 signaling and CDK-Beta-catenin activity	1,38	0,101	3	15
WP: Sulfation Biotransformation Reaction	1,38	0,109	3	15
KEGG: Parathyroid hormone synthesis, secretion and action	1,37	0,16	14	104
WP: TGF-beta Signaling Pathway	1,35	0,154	17	131
Reactome: Circadian Clock	1,34	0,185	9	62
Reactome: Fcgamma receptor (FCGR) dependent phagocytosis	1,33	0,182	11	79
KEGG: Galactose metabolism	1,33	0,163	5	30
WP: PI3K-AKT-mTOR signaling pathway and therapeutic opportunities	1,33	0,106	5	30
WP: Oligodendrocyte Specification and differentiation(including remyelination), leading to Myelin Components for CNS	1,33	0,189	5	30
WP: ESC Pluripotency Pathways	1,32	0,169	15	114
KEGG: Metabolism of xenobiotics by cytochrome P450	1,32	0,173	7	46
Reactome: Signaling by PTK6	1,32	0,182	7	46
WP: ErbB Signaling Pathway	1,32	0,199	12	88
KEGG: Melanogenesis	1,3	0,205	13	97
Reactome: Butyrophilin (BTN) family interactions	1,3	0,126	2	9
WP: Codeine and Morphine Metabolism	1,3	0,118	2	9
WP: Composition of Lipid Particles	1,3	0,112	2	9
WP: Degradation pathway of sphingolipids, including diseases	1,3	0,127	2	9
WP: Steroid Biosynthesis	1,3	0,113	2	9
Reactome: Transcriptional regulation by RUNX3	1,29	0,184	14	106
WP: Breast cancer pathway	1,28	0,187	19	151
Reactome: Interferon gamma signaling	1,26	0,2	10	72
Reactome: Nephrin family interactions	1,26	0,25	3	16
WP: Osteoclast Signaling	1,26	0,117	3	16
WP: Hepatitis C and Hepatocellular Carcinoma	1,25	0,229	7	47
WP: Myometrial Relaxation and Contraction Pathways	1,25	0,226	19	152
WP: White fat cell differentiation	1,25	0,171	5	31
KEGG: Ferroptosis	1,25	0,223	6	39
WP: Oxidative Damage	1,25	0,258	6	39
WP: Ferroptosis	1,25	0,222	6	39
KEGG: Glycosaminoglycan biosynthesis	1,19	0,158	4	24
Reactome: Miscellaneous transport and binding events	1,19	0,248	4	24
KEGG: Cell adhesion molecules (CAMs)	1,18	0,259	15	118
KEGG: Carbohydrate digestion and absorption	1,18	0,228	6	40
KEGG: Starch and sucrose metabolism	1,18	0,212	5	32
KEGG: PPAR signaling pathway	1,17	0,262	10	74
Reactome: Gastrin-CREB signalling pathway via PKC and MAPK	1,14	0,304	3	17
Reactome: Aflatoxin activation and detoxification	1,14	0,251	3	17
Reactome: Cellular hexose transport	1,14	0,255	3	17
WP: Glutathione metabolism	1,14	0,261	3	17
WP: Canonical and Non-Canonical TGF-B signaling	1,14	0,132	3	17
Reactome: Transcriptional activity of SMAD2/SMAD3:SMAD4 heterotrimer	1,14	0,255	7	49
WP: Irinotecan Pathway	1,13	0,138	2	10
WP: Trans-sulfuration pathway	1,13	0,152	2	10
WP: TNF related weak inducer of apoptosis (TWEAK) Signaling Pathway	1,11	0,321	6	41

WP: IL-2 Signaling Pathway	1,11	0,319	6	41
Reactome: Signaling by FGFR1	1,11	0,269	8	58
Reactome: Interleukin-7 signaling	1,1	0,268	4	25
WP: IL-7 Signaling Pathway	1,1	0,208	4	25
WP: PPAR Alpha Pathway	1,1	0,228	4	25
WP: Hypothesized Pathways in Pathogenesis of Cardiovascular Disease	1,1	0,202	4	25
WP: Insulin Signaling	1,07	0,275	19	158
Reactome: NGF processing	1,05	0,129	1	4
WP: Nicotine Activity on Chromaffin Cells	1,05	0,143	1	4
WP: Neurotransmitter Disorders	1,05	0,187	1	4
WP: MicroRNA network associated with chronic lymphocytic leukemia	1,05	0,117	1	4
WP: Serotonin Receptor 2 and STAT3 Signaling	1,05	0,149	1	4
KEGG: Aminoacyl-tRNA biosynthesis	1,05	0,302	6	42
KEGG: Pantothenate and CoA biosynthesis	1,03	0,345	3	18
WP: Farnesoid X Receptor Pathway	1,03	0,191	3	18
WP: Hematopoietic Stem Cell Gene Regulation by GABP alpha/beta Complex	1,03	0,256	3	18
Reactome: Signaling by FGFR3	1,03	0,266	5	34
WP: Fatty Acid Beta Oxidation	1,03	0,305	5	34
Reactome: Toll-like Receptor Cascades	1,02	0,316	4	26
Reactome: Sulfur amino acid metabolism	1,02	0,343	4	26
WP: Constitutive Androstane Receptor Pathway	1,02	0,396	4	26
WP: Wnt/beta-catenin Signaling Pathway in Leukemia	1,02	0,333	4	26
KEGG: Longevity regulating pathway	1,01	0,291	8	60
WP: Non-genomic actions of 1,25 dihydroxyvitamin D3	1	0,329	9	69
KEGG: Longevity regulating pathway	0,99	0,315	11	87
WP: Pancreatic adenocarcinoma pathway	0,99	0,307	11	87
KEGG: Purine metabolism	0,99	0,313	19	161
WP: Nucleotide GPCRs	0,98	0,177	2	11
Reactome: PI3K Cascade	0,96	0,326	5	35
WP: Selenium Metabolism and Selenoproteins	0,96	0,326	5	35
Reactome: Cell junction organization	0,95	0,351	10	79
Reactome: Phase I - Functionalization of compounds	0,95	0,331	10	79
KEGG: Pentose phosphate pathway	0,94	0,322	4	27
WP: BMP Signaling Pathway in Eyelid Development	0,93	0,334	3	19
WP: Extracellular vesicles in the crosstalk of cardiac cells	0,93	0,427	3	19
KEGG: Endocrine and other factor-regulated calcium reabsorption	0,93	0,369	6	44
Reactome: Signaling by EGFR	0,93	0,356	6	44
WP: Thymic Stromal LymphoPoietin (TSLP) Signaling Pathway	0,93	0,355	6	44
KEGG: Signaling pathways regulating pluripotency of stem cells	0,92	0,356	16	135
Reactome: Gene and protein expression by JAK-STAT signaling after Interleukin-12 stimulation	0,91	0,381	9	71
WP: Arrhythmogenic Right Ventricular Cardiomyopathy	0,91	0,373	9	71
Reactome: Transport of vitamins, nucleosides, and related molecules	0,89	0,386	5	36
Reactome: Signaling by FGFR4	0,89	0,359	5	36
WP: Notch Signaling Pathway	0,87	0,358	6	45
KEGG: AMPK signaling pathway	0,87	0,391	14	118
Reactome: TP53 Regulates Transcription of Cell Death Genes	0,86	0,373	8	63
Reactome: Myogenesis	0,86	0,374	4	28
Reactome: Metabolism of steroid hormones	0,86	0,388	4	28
Reactome: TNFs bind their physiological receptors	0,86	0,38	4	28
Reactome: Signaling by Rho GTPases	0,85	0,427	15	128
Reactome: Advanced glycosylation endproduct receptor signaling	0,84	0,529	2	12
WP: Purine metabolism	0,84	0,428	2	12
KEGG: Histidine metabolism	0,83	0,414	3	20
KEGG: Calcium signaling pathway	0,83	0,434	20	176
KEGG: NOD-like receptor signaling pathway	0,83	0,383	16	138
KEGG: Aldosterone-regulated sodium reabsorption	0,83	0,433	5	37
Reactome: Neurotransmitter release cycle	0,83	0,404	5	37
WP: Fibrin Complement Receptor 3 Signaling Pathway	0,83	0,448	5	37
KEGG: ErbB signaling pathway	0,82	0,41	10	82
WP: Hair Follicle Development: Cytodifferentiation (Part 3 of 3)	0,82	0,405	10	82
WP: Oncostatin M Signaling Pathway	0,81	0,431	8	64
KEGG: Drug metabolism	0,81	0,435	7	55
Reactome: Signaling by NOTCH3	0,81	0,417	7	55
KEGG: Phosphonate and phosphinate metabolism	0,8	0,415	1	5
Reactome: Formation of xylulose-5-phosphate	0,8	0,55	1	5
Reactome: Reelin signalling pathway	0,8	0,294	1	5
Reactome: Biosynthesis of EPA-derived SPMs	0,8	0,41	1	5
WP: Sulindac Metabolic Pathway	0,8	0,228	1	5
WP: Vitamin B6-dependent and responsive disorders	0,8	0,531	1	5
WP: Arachidonate Epoxygenase / Epoxide Hydrolase	0,8	0,359	1	5
Reactome: Transcriptional regulation of white adipocyte differentiation	0,8	0,437	11	92
KEGG: Circadian rhythm	0,78	0,515	4	29
WP: PDGFR-beta pathway	0,78	0,541	4	29
KEGG: C-type lectin receptor signaling pathway	0,77	0,434	12	102

WP: AMP-activated Protein Kinase (AMPK) Signaling	0,76	0,475	8	65
Reactome: Activation of NMDA receptors and postsynaptic events	0,76	0,451	5	38
WP: PDGF Pathway	0,76	0,416	5	38
WP: Bladder Cancer	0,76	0,427	5	38
KEGG: Notch signaling pathway	0,76	0,41	6	47
KEGG: cAMP signaling pathway	0,74	0,468	21	189
Reactome: Growth hormone receptor signaling	0,74	0,485	3	21
WP: PI3K/AKT/mTOR - VitD3 Signalling	0,74	0,545	3	21
Reactome: Regulated Necrosis	0,72	0,469	2	13
WP: Osteopontin Signaling	0,72	0,588	2	13
WP: Estrogen Receptor Pathway	0,72	0,635	2	13
WP: Serotonin and anxiety-related events	0,72	0,594	2	13
KEGG: Porphyrin and chlorophyll metabolism	0,71	0,477	4	30
WP: Zinc homeostasis	0,71	0,513	4	30
KEGG: Amino sugar and nucleotide sugar metabolism	0,7	0,49	6	48
KEGG: Steroid hormone biosynthesis	0,7	0,487	5	39
WP: Prolactin Signaling Pathway	0,69	0,504	9	76
KEGG: Glucagon signaling pathway	0,68	0,474	11	95
KEGG: Insulin signaling pathway	0,66	0,489	15	134
KEGG: alpha-Linolenic acid metabolism	0,66	0,529	3	22
WP: Globo Sphingolipid Metabolism	0,66	0,574	3	22
WP: NRF2-ARE regulation	0,66	0,598	3	22
WP: Estrogen signaling pathway	0,66	0,624	3	22
KEGG: Vascular smooth muscle contraction	0,65	0,522	13	115
KEGG: Osteoclast differentiation	0,65	0,522	13	115
Reactome: Nucleotide-binding domain, leucine rich repeat containing receptor (NLR) signaling pathways	0,65	0,492	6	49
WP: IL-5 Signaling Pathway	0,64	0,476	5	40
Reactome: GPVI-mediated activation cascade	0,64	0,534	4	31
Reactome: MAPK targets/ Nuclear events mediated by MAP kinases	0,64	0,555	4	31
Reactome: DAP12 interactions	0,64	0,519	4	31
WP: Factors and pathways affecting insulin-like growth factor (IGF1)-Akt signaling	0,64	0,58	4	31
Reactome: Unfolded Protein Response (UPR)	0,61	0,547	2	14
Reactome: Synthesis of Lipoxins (LX)	0,6	0,555	1	6
WP: let-7 inhibition of ES cell reprogramming	0,6	0,719	1	6
WP: Robo4 and VEGF Signaling Pathways Crosstalk	0,6	0,337	1	6
WP: SCFA and skeletal muscle substrate metabolism	0,6	0,72	1	6
WP: Sudden Infant Death Syndrome (SIDS) Susceptibility Pathways	0,58	0,547	17	156
WP: EBV LMP1 signaling	0,57	0,682	3	23
KEGG: Tyrosine metabolism	0,57	0,561	4	32
KEGG: Cortisol synthesis and secretion	0,57	0,587	7	60
KEGG: Regulation of actin cytoskeleton	0,54	0,595	22	207
WP: Splicing factor NOVA regulated synaptic proteins	0,53	0,586	5	42
WP: G Protein Signaling Pathways	0,51	0,602	10	90
Reactome: Sialic acid metabolism	0,51	0,645	4	33
KEGG: Cushing syndrome	0,51	0,613	16	149
KEGG: Selenocompound metabolism	0,5	0,6	2	15
Reactome: Metabolism of folate and pterines	0,5	0,596	2	15
Reactome: Nucleobase biosynthesis	0,5	0,661	2	15
Reactome: Signaling by NTRK3 (TRKC)	0,5	0,595	2	15
WP: GPCRs, Class B Secretin-like	0,5	0,692	2	15
WP: Biogenic Amine Synthesis	0,5	0,588	2	15
WP: ACE Inhibitor Pathway	0,5	0,637	2	15
KEGG: Linoleic acid metabolism	0,5	0,691	3	24
KEGG: Vitamin digestion and absorption	0,5	0,665	3	24
Reactome: Interconversion of nucleotide di- and triphosphates	0,5	0,62	3	24
WP: Differentiation of white and brown adipocyte	0,5	0,645	3	24
KEGG: Phagosome	0,49	0,672	13	120
Reactome: Regulation of lipid metabolism by Peroxisome proliferator-activated receptor alpha (PPARalpha)	0,48	0,634	16	150
KEGG: Mitophagy	0,47	0,669	7	62
WP: Interleukin-11 Signaling Pathway	0,47	0,644	5	43
KEGG: Ras signaling pathway	0,45	0,64	23	221
KEGG: Alanine, aspartate and glutamate metabolism	0,44	0,65	4	34
Reactome: MyD88:MAL(TIRAP) cascade initiated on plasma membrane	0,44	0,634	4	34
WP: p38 MAPK Signaling Pathway	0,44	0,667	4	34
KEGG: Circadian entrainment	0,44	0,667	10	92
Reactome: Interleukin-1 processing	0,43	0,8	1	7
Reactome: OAS antiviral response	0,43	0,674	1	7
WP: Effects of Nitric Oxide	0,43	0,873	1	7
WP: FTO Obesity Variant Mechanism	0,43	0,921	1	7
WP: Hypothetical Craniofacial Development Pathway	0,43	0,938	1	7
WP: Tgif disruption of Shh signaling	0,43	0,895	1	7
WP: Hfe effect on hepcidin production	0,43	0,856	1	7
WP: Aflatoxin B1 metabolism	0,43	0,871	1	7

WP: Signal Transduction of S1P Receptor	0,42	0,717	3	25
KEGG: Retinol metabolism	0,42	0,706	5	44
KEGG: Drug metabolism	0,42	0,672	5	44
WP: Oxidation by Cytochrome P450	0,42	0,704	5	44
WP: MicroRNAs in cardiomyocyte hypertrophy	0,41	0,671	9	83
KEGG: Nitrogen metabolism	0,41	0,638	2	16
WP: SREBF and miR33 in cholesterol and lipid homeostasis	0,41	0,869	2	16
Reactome: Signaling by MET	0,4	0,7	6	54
KEGG: Neuroactive ligand-receptor interaction	0,39	0,719	27	264
Reactome: Signaling by NTRK1 (TRKA)	0,38	0,691	8	74
WP: RANKL/RANK (Receptor activator of NFKB (ligand)) Signaling Pathway	0,35	0,717	6	55
WP: Hematopoietic Stem Cell Differentiation	0,35	0,733	6	55
WP: Non-small cell lung cancer	0,34	0,745	7	65
Reactome: Signaling by NOTCH1	0,34	0,752	8	75
Reactome: Regulation of Hypoxia-inducible Factor (HIF) by oxygen	0,34	0,731	8	75
WP: Striated Muscle Contraction	0,32	0,73	4	36
WP: miRNAs involvement in the immune response in sepsis	0,32	0,742	4	36
WP: Regulation of Wnt/B-catenin Signaling by Small Molecule Compounds	0,31	0,693	2	17
WP: Mitochondrial LC-Fatty Acid Beta-Oxidation	0,31	0,732	2	17
WP: Leptin Insulin Overlap	0,31	0,824	2	17
WP: Serotonin and anxiety	0,31	0,809	2	17
KEGG: Phospholipase D signaling pathway	0,31	0,766	14	136
KEGG: Platelet activation	0,3	0,753	12	116
WP: Association Between Physico-Chemical Features and Toxicity Associated Pathways	0,3	0,779	7	66
KEGG: Toll-like receptor signaling pathway	0,3	0,76	9	86
KEGG: Sulfur metabolism	0,29	0,817	1	8
Reactome: GP1b-IX-V activation signalling	0,29	0,733	1	8
WP: Folate-Alcohol and Cancer Pathway Hypotheses	0,29	0,745	1	8
WP: NAD Biosynthesis II (from tryptophan)	0,29	0,734	1	8
WP: NLR Proteins	0,29	0,98	1	8
WP: Insulin signalling in human adipocytes (normal condition)	0,29	0,938	1	8
WP: Insulin signalling in human adipocytes (diabetic condition)	0,29	0,977	1	8
WP: ApoE and miR-146 in inflammation and atherosclerosis	0,29	0,989	1	8
WP: Methylation Pathways	0,29	0,952	1	8
WP: G13 Signaling Pathway	0,27	0,801	4	37
Reactome: Intrinsic Pathway for Apoptosis	0,26	0,794	5	47
WP: Peptide GPCRs	0,26	0,811	7	67
KEGG: mTOR signaling pathway	0,25	0,787	15	148
KEGG: Steroid biosynthesis	0,23	0,822	2	18
KEGG: One carbon pool by folate	0,23	0,748	2	18
Reactome: Signaling by Hippo	0,23	0,829	2	18
Reactome: RHO GTPases activate CIT	0,23	0,804	2	18
Reactome: Amine-derived hormones	0,23	0,821	2	18
WP: Simplified Interaction Map Between LOXL4 and Oxidative Stress Pathway	0,23	0,927	2	18
WP: 4-hydroxytamoxifen, Dexamethasone, and Retinoic Acids Regulation of p27 Expression	0,23	0,73	2	18
WP: Toll-like Receptor Signaling Pathway	0,22	0,826	9	88
KEGG: Bile secretion	0,22	0,805	7	68
Reactome: Surfactant metabolism	0,21	0,826	3	28
WP: One Carbon Metabolism	0,21	0,813	3	28
Reactome: ISG15 antiviral mechanism	0,21	0,822	6	58
WP: Kit receptor signaling pathway	0,21	0,841	6	58
KEGG: Cytosolic DNA-sensing pathway	0,21	0,834	5	48
KEGG: Pyruvate metabolism	0,21	0,852	4	38
Reactome: Signaling by SCF-KIT	0,21	0,815	4	38
WP: MAPK Signaling Pathway	0,21	0,828	24	242
Reactome: Opioid Signalling	0,18	0,841	8	79
Reactome: Sphingolipid metabolism	0,18	0,844	8	79
KEGG: cGMP-PKG signaling pathway	0,18	0,851	16	161
KEGG: Adipocytokine signaling pathway	0,17	0,872	7	69
Reactome: Neurotransmitter clearance	0,16	0,889	1	9
Reactome: Signaling by Leptin	0,16	0,846	1	9
WP: Macrophage markers	0,16	0,99	1	9
WP: Thiamine metabolic pathways	0,16	0,817	1	9
WP: Angiopoietin Like Protein 8 Regulatory Pathway	0,15	0,879	13	131
WP: Canonical and Non-canonical Notch signaling	0,15	0,825	3	29
KEGG: Glycosaminoglycan degradation	0,15	0,9	2	19
Reactome: RHO GTPases Activate ROCKs	0,15	0,874	2	19
Reactome: Synthesis, secretion, and deacylation of Ghrelin	0,15	0,888	2	19
WP: Nucleotide Metabolism	0,15	0,865	2	19
KEGG: Leukocyte transendothelial migration	0,14	0,892	11	111
WP: Metapathway biotransformation Phase I and II	0,13	0,886	14	142
Reactome: Metabolism of water-soluble vitamins and cofactors	0,13	0,903	7	70
WP: Neural Crest Differentiation	0,13	0,907	10	101
Reactome: ESR-mediated signaling	0,12	0,896	12	122

WP: Structural Pathway of Interleukin 1 (IL-1)	0,11	0,918	5	50
WP: Preimplantation Embryo	0,11	0,901	5	50
WP: Signaling Pathways in Glioblastoma	0,11	0,91	8	81
KEGG: Ether lipid metabolism	0,1	0,91	4	40
WP: Aryl Hydrocarbon Receptor Pathway	0,1	0,941	4	40
KEGG: beta-Alanine metabolism	0,09	0,909	3	30
KEGG: Glyoxylate and dicarboxylate metabolism	0,09	0,899	3	30
Reactome: Cargo concentration in the ER	0,09	0,925	3	30
WP: Development and heterogeneity of the ILC family	0,09	0,889	3	30
WP: Prion disease pathway	0,09	0,917	3	30
KEGG: Glycosaminoglycan biosynthesis	0,07	0,878	2	20
Reactome: rRNA modification in the nucleus and cytosol	0,07	0,923	2	20
WP: Nanomaterial induced apoptosis	0,07	0,871	2	20
WP: Hereditary leiomyomatosis and renal cell carcinoma pathway	0,07	0,935	2	20
KEGG: Necroptosis	0,07	0,936	13	134
Reactome: Translocation of SLC2A4 (GLUT4) to the plasma membrane	0,07	0,934	5	51
WP: Regulation of Actin Cytoskeleton	0,05	0,944	14	145
Reactome: Platelet Aggregation (Plug Formation)	0,05	0,891	1	10
Reactome: Trafficking and processing of endosomal TLR	0,05	0,931	1	10
Reactome: RHO GTPases activate IQGAPs	0,05	0,883	1	10
Reactome: POU5F1 (OCT4), SOX2, NANOG repress genes related to differentiation	0,05	0,911	1	10
Reactome: Ketone body metabolism	0,05	0,955	1	10
Reactome: Tetrahydrobiopterin (BH4) synthesis, recycling, salvage and regulation	0,05	0,958	1	10
WP: SRF and miRs in Smooth Muscle Differentiation and Proliferation	0,05	0,966	1	10
WP: Liver X Receptor Pathway	0,05	0,996	1	10
WP: Valproic acid pathway	0,05	0,911	1	10
WP: Ethanol metabolism resulting in production of ROS by CYP2E1	0,05	0,874	1	10
WP: Glycogen Metabolism	0,05	0,973	4	41
Reactome: Signal amplification	0,03	0,97	3	31
Reactome: Thrombin signalling through proteinase activated receptors (PARs)	0,03	0,971	3	31
Reactome: Carboxyterminal post-translational modifications of tubulin	0,03	0,975	3	31
WP: Monoamine GPCRs	0,03	0,992	3	31
KEGG: Thyroid hormone synthesis	0,02	0,996	7	73
KEGG: Neurotrophin signaling pathway	0,01	0,977	11	115
KEGG: Pentose and glucuronate interconversions	0	0,998	2	21
Reactome: Cholesterol biosynthesis	0	0,999	2	21
WP: Type II diabetes mellitus	0	0,999	2	21
Reactome: DDX58/IFIH1-mediated induction of interferon-alpha/beta	0	0,998	6	63
KEGG: Rap1 signaling pathway	-0,01	0,993	19	200
WP: Mesodermal Commitment Pathway	-0,02	0,992	13	137
WP: Interferon type I signaling pathways	-0,02	0,96	5	53
Reactome: Transport of bile salts and organic acids, metal ions and amine compounds	-0,04	0,968	8	85
Reactome: Hedgehog ligand biogenesis	-0,04	0,979	6	64
KEGG: Taurine and hypotaurine metabolism	-0,05	0,936	1	11
Reactome: LGI-ADAM interactions	-0,05	0,937	1	11
WP: Serotonin Transporter Activity	-0,05	0,993	1	11
WP: Vitamin D Metabolism	-0,05	0,975	1	11
WP: Fatty Acid Omega Oxidation	-0,05	0,987	1	11
WP: Gene regulatory network modelling somitogenesis	-0,05	0,998	1	11
WP: NAD metabolism, sirtuins and aging	-0,05	0,997	1	11
Reactome: Vasopressin regulates renal water homeostasis via Aquaporins	-0,05	0,948	4	43
WP: Tryptophan metabolism	-0,05	0,95	4	43
WP: Integrin-mediated Cell Adhesion	-0,05	0,948	9	96
WP: EGF/EGFR Signaling Pathway	-0,07	0,959	15	160
KEGG: Metabolic pathways	-0,07	0,951	111	1172
KEGG: Nicotinate and nicotinamide metabolism	-0,07	0,934	2	22
KEGG: Biosynthesis of unsaturated fatty acids	-0,07	0,953	2	22
WP: miRNA regulation of p53 pathway in prostate cancer	-0,07	0,913	2	22
KEGG: Glycerophospholipid metabolism	-0,07	0,917	8	86
WP: Signaling of Hepatocyte Growth Factor Receptor	-0,09	0,904	3	33
Reactome: G alpha (12/13) signalling events	-0,09	0,932	7	76
KEGG: Glutamatergic synapse	-0,1	0,934	10	108
Reactome: EPH-Ephrin signaling	-0,11	0,92	8	87
Reactome: Visual phototransduction	-0,11	0,912	5	55
Reactome: Signaling by FGFR2	-0,12	0,909	6	66
Reactome: PIP3 activates AKT signaling	-0,13	0,891	10	109
Reactome: Factors involved in megakaryocyte development and platelet production	-0,13	0,884	7	77
WP: GPCRs, Class A Rhodopsin-like	-0,14	0,901	20	216
Reactome: Branched-chain amino acid catabolism	-0,14	0,897	2	23
WP: Nanoparticle triggered autophagic cell death	-0,14	0,894	2	23
WP: Blood Clotting Cascade	-0,14	0,895	2	23
WP: Fatty Acid Biosynthesis	-0,14	0,914	2	23
Reactome: Triglyceride metabolism	-0,14	0,891	3	34
Reactome: Prolactin receptor signaling	-0,14	0,897	1	12

Reactome: alpha-linolenic (omega3) and linoleic (omega6) acid metabolism	-0,14	0,877	1	12
Reactome: Reversible hydration of carbon dioxide	-0,14	0,85	1	12
Reactome: Import of palmitoyl-CoA into the mitochondrial matrix	-0,14	0,916	1	12
WP: Alanine and aspartate metabolism	-0,14	0,865	1	12
WP: Bone Morphogenic Protein (BMP) Signalling and Regulation	-0,14	0,993	1	12
WP: Cell Differentiation - Index	-0,14	0,865	1	12
WP: Dopamine metabolism	-0,14	0,919	1	12
WP: Nanoparticle triggered regulated necrosis	-0,14	0,871	1	12
WP: Aryl Hydrocarbon Receptor	-0,15	0,889	4	45
KEGG: RIG-I-like receptor signaling pathway	-0,15	0,877	5	56
WP: miRNA Regulation of DNA Damage Response	-0,16	0,871	6	67
Reactome: PI Metabolism	-0,17	0,856	7	78
KEGG: Apelin signaling pathway	-0,17	0,878	12	132
Reactome: Amyloid fiber formation	-0,19	0,857	4	46
WP: Regulation of Microtubule Cytoskeleton	-0,19	0,902	4	46
Reactome: Signaling by NOTCH2	-0,19	0,857	3	35
Reactome: Toll Like Receptor 3 (TLR3) Cascade	-0,19	0,85	3	35
Reactome: Mitochondrial Fatty Acid Beta-Oxidation	-0,19	0,875	3	35
Reactome: Antimicrobial peptides	-0,19	0,865	3	35
WP: Nucleotide-binding Oligomerization Domain (NOD) pathway	-0,19	0,885	3	35
WP: RIG-I-like Receptor Signaling	-0,19	0,856	5	57
KEGG: Fatty acid elongation	-0,2	0,854	2	24
Reactome: Signaling by Erythropoietin	-0,2	0,848	2	24
WP: Physiological and Pathological Hypertrophy of the Heart	-0,2	0,797	2	24
KEGG: Aldosterone synthesis and secretion	-0,21	0,874	8	90
KEGG: Fatty acid biosynthesis	-0,23	0,8	1	13
KEGG: Thiamine metabolism	-0,23	0,864	1	13
Reactome: Signaling by Activin	-0,23	0,85	1	13
WP: Development of pulmonary dendritic cells and macrophage subsets	-0,23	0,983	1	13
WP: Kennedy pathway from Sphingolipids	-0,23	0,767	1	13
WP: Vitamin B12 Disorders	-0,23	0,753	1	13
WP: Hedgehog Signaling Pathway	-0,23	0,961	1	13
Reactome: Iron uptake and transport	-0,24	0,825	5	58
KEGG: Ribosome biogenesis in eukaryotes	-0,24	0,799	6	69
KEGG: Fat digestion and absorption	-0,24	0,81	3	36
Reactome: MyD88-independent TLR4 cascade	-0,24	0,803	3	36
WP: Target Of Rapamycin (TOR) Signaling	-0,24	0,803	3	36
Reactome: Insulin processing	-0,26	0,774	2	25
Reactome: Glutamate binding, activation of AMPA receptors and synaptic plasticity	-0,26	0,775	2	25
WP: Follicle Stimulating Hormone (FSH) signaling pathway	-0,26	0,756	2	25
WP: Pregnane X Receptor pathway	-0,26	0,773	2	25
KEGG: Th17 cell differentiation	-0,27	0,795	8	92
Reactome: p75 NTR receptor-mediated signalling	-0,27	0,784	8	92
Reactome: Senescence-Associated Secretory Phenotype (SASP)	-0,27	0,797	6	70
KEGG: Thyroid hormone signaling pathway	-0,28	0,797	10	114
WP: Notch Signaling Pathway	-0,28	0,784	5	59
WP: IL-3 Signaling Pathway	-0,28	0,804	4	48
KEGG: Tryptophan metabolism	-0,29	0,785	3	37
WP: Hedgehog Signaling Pathway	-0,29	0,812	3	37
KEGG: Glycosaminoglycan biosynthesis	-0,3	0,965	1	14
Reactome: SUMOylation of DNA methylation proteins	-0,3	0,75	1	14
Reactome: Acetylcholine binding and downstream events	-0,3	0,654	1	14
WP: Cholesterol Biosynthesis	-0,3	0,704	1	14
WP: ERK Pathway in Huntington's Disease	-0,3	0,92	1	14
WP: GPR40 Pathway	-0,3	0,854	1	14
WP: Tryptophan catabolism leading to NAD+ production	-0,3	0,779	1	14
KEGG: Insulin secretion	-0,31	0,762	7	82
Reactome: Metabolism of polyamines	-0,31	0,767	7	82
KEGG: RNA degradation	-0,31	0,772	6	71
WP: Chemokine signaling pathway	-0,31	0,74	14	159
KEGG: Chemokine signaling pathway	-0,31	0,759	15	170
Reactome: Signaling by BMP	-0,32	0,765	2	26
WP: Nanoparticle-mediated activation of receptor signaling	-0,32	0,669	2	26
Reactome: Latent infection of Homo sapiens with Mycobacterium tuberculosis	-0,32	0,76	0	1
Reactome: Abnormal conversion of 2-oxoglutarate to 2-hydroxyglutarate	-0,32	0,829	0	1
Reactome: NADPH regeneration	-0,32	0,961	0	1
Reactome: Vitamin E	-0,32	0,689	0	1
WP: Proprotein convertase subtilisin/kexin type 9 (PCSK9) mediated LDL receptor degradation	-0,32	0,325	0	1
WP: mir34a and TGIF2 in osteoclastogenesis	-0,32	0,603	0	1
WP: Evolocumab Mechanism	-0,32	0,615	0	1
WP: Gut-Liver Indole Metabolism	-0,32	0,831	0	1
WP: Metabolism of Dichloroethylene by CYP450	-0,32	0,945	0	1
WP: Acrylamide Biotransformation and Exposure Biomarkers	-0,32	0,923	0	1
WP: FABP4 in ovarian cancer	-0,32	0,318	0	1

WP: Glucose Homeostasis	-0,32	0,769	0	1
WP: Regulation of toll-like receptor signaling pathway	-0,33	0,723	11	127
Reactome: Signaling by TGF-beta Receptor Complex	-0,34	0,73	3	38
WP: Common Pathways Underlying Drug Addiction	-0,34	0,738	3	38
WP: Pathways Affected in Adenoid Cystic Carcinoma	-0,35	0,725	5	61
KEGG: Estrogen signaling pathway	-0,36	0,689	11	128
Reactome: The citric acid (TCA) cycle and respiratory electron transport	-0,37	0,718	4	50
Reactome: Effects of PIP2 hydrolysis	-0,38	0,724	2	27
Reactome: TAK1 activates NFkB by phosphorylation and activation of IKKs complex	-0,38	0,766	2	27
Reactome: Glycogen metabolism	-0,38	0,72	2	27
KEGG: Glycosphingolipid biosynthesis	-0,38	0,938	1	15
KEGG: Glycosphingolipid biosynthesis	-0,38	0,936	1	15
Reactome: Metabolism of nitric oxide	-0,38	0,718	1	15
Reactome: Defensins	-0,38	0,715	1	15
Reactome: Metabolism of Angiotensinogen to Angiotensins	-0,38	0,712	1	15
Reactome: Regulation of RUNX1 Expression and Activity	-0,38	0,7	1	15
WP: Role of Osx and miRNAs in tooth development	-0,38	0,672	1	15
WP: Microglia Pathogen Phagocytosis Pathway	-0,39	0,706	3	39
WP: MECP2 and Associated Rett Syndrome	-0,39	0,674	5	62
Reactome: Signaling by NOTCH4	-0,41	0,71	7	85
WP: Wnt Signaling Pathway	-0,41	0,712	4	51
Reactome: RHO GTPases activate PKNs	-0,43	0,674	2	28
WP: miRs in Muscle Cell Differentiation	-0,43	0,698	2	28
KEGG: Hedgehog signaling pathway	-0,44	0,693	3	40
Reactome: Signaling by ERBB2	-0,44	0,666	3	40
Reactome: SUMOylation of transcription cofactors	-0,44	0,68	3	40
KEGG: Fc gamma R-mediated phagocytosis	-0,44	0,658	7	86
Reactome: Signaling by NODAL	-0,45	0,656	1	16
WP: Drug Induction of Bile Acid Pathway	-0,45	0,626	1	16
WP: TCA Cycle and Deficiency of Pyruvate Dehydrogenase complex (PDHc)	-0,45	0,609	1	16
WP: NOTCH1 regulation of human endothelial cell calcification	-0,45	0,833	1	16
WP: ID signaling pathway	-0,45	0,852	1	16
WP: Genotoxicity pathway	-0,45	0,673	4	52
Reactome: TCF dependent signaling in response to WNT	-0,45	0,628	16	187
Reactome: Pyrophosphate hydrolysis	-0,46	0,757	0	2
Reactome: Plasmalogen biosynthesis	-0,46	0,769	0	2
Reactome: Intestinal infectious diseases	-0,46	0,424	0	2
WP: Butyrate-induced histone acetylation	-0,46	0,81	0	2
WP: Hypoxia-mediated EMT and Stemness	-0,46	0,446	0	2
WP: Mevalonate arm of cholesterol biosynthesis pathway with inhibitors	-0,46	0,683	0	2
WP: Hormonal control of Pubertal Growth Spurt	-0,46	0,641	0	2
WP: Ultraconserved region 339 modulation of tumor suppressor microRNAs in cancer	-0,46	0,439	0	2
KEGG: Adherens junction	-0,47	0,635	5	64
Reactome: Plasma lipoprotein assembly, remodeling, and clearance	-0,47	0,653	5	64
WP: DNA Damage Response	-0,47	0,644	5	64
KEGG: Progesterone-mediated oocyte maturation	-0,47	0,65	7	87
KEGG: Antigen processing and presentation	-0,48	0,673	3	41
Reactome: Interleukin-2 family signaling	-0,48	0,66	2	29
WP: IL17 signaling pathway	-0,48	0,635	2	29
WP: Dopaminergic Neurogenesis	-0,48	0,638	2	29
WP: Ethanol effects on histone modifications	-0,48	0,649	2	29
WP: MAPK Cascade	-0,48	0,641	2	29
WP: Nonalcoholic fatty liver disease	-0,49	0,672	12	144
Reactome: Costimulation by the CD28 family	-0,49	0,618	4	53
KEGG: Fc epsilon RI signaling pathway	-0,5	0,608	5	65
KEGG: Primary bile acid biosynthesis	-0,51	0,618	1	17
KEGG: Mannose type O-glycan biosynthesis	-0,51	0,641	1	17
KEGG: 2-Oxocarboxylic acid metabolism	-0,51	0,618	1	17
WP: IL-9 Signaling Pathway	-0,51	0,787	1	17
WP: Simplified Depiction of MYD88 Distinct Input-Output Pathway	-0,51	0,753	1	17
WP: Mitochondrial Gene Expression	-0,51	0,721	1	17
KEGG: Th1 and Th2 cell differentiation	-0,52	0,615	6	77
Reactome: Platelet homeostasis	-0,52	0,596	6	77
KEGG: Fatty acid degradation	-0,53	0,604	3	42
KEGG: ABC transporters	-0,53	0,592	3	42
WP: Fas Ligand (FasL) pathway and Stress induction of Heat Shock Proteins (HSP) regulation	-0,53	0,598	3	42
WP: Cardiac Hypertrophic Response	-0,53	0,611	4	54
KEGG: Citrate cycle (TCA cycle)	-0,53	0,593	2	30
Reactome: Glyoxylate metabolism and glycine degradation	-0,53	0,604	2	30
WP: Toll-like Receptor Signaling	-0,53	0,614	2	30
Reactome: MAPK6/MAPK4 signaling	-0,54	0,612	7	89
WP: Human Thyroid Stimulating Hormone (TSH) signaling pathway	-0,54	0,609	5	66
WP: RAC1/PAK1/p38/MMP2 Pathway	-0,54	0,579	5	66
KEGG: D-Glutamine and D-glutamate metabolism	-0,56	0,773	0	3

Reactome: Insulin-like Growth Factor-2 mRNA Binding Proteins (IGF2BPs/IMPs/VICKZs) bind RNA	-0,56	0,713	0	3
Reactome: RHO GTPases regulate CFTR trafficking	-0,56	0,719	0	3
Reactome: Threonine catabolism	-0,56	0,669	0	3
Reactome: Metabolism of vitamin K	-0,56	0,726	0	3
Reactome: Transcription from mitochondrial promoters	-0,56	0,762	0	3
Reactome: Intracellular oxygen transport	-0,56	0,762	0	3
Reactome: rRNA modification in the mitochondrion	-0,56	0,77	0	3
WP: Nicotine Metabolism	-0,56	0,761	0	3
WP: Diclofenac Metabolic Pathway	-0,56	0,698	0	3
WP: Vitamins A and D - action mechanisms	-0,56	0,742	0	3
WP: Model for regulation of MSMP expression in cancer cells and its proangiogenic role in ovarian tumors	-0,56	0,272	0	3
WP: Amino acid conjugation of benzoic acid	-0,56	0,735	0	3
WP: TNF alpha Signaling Pathway	-0,57	0,6	7	90
KEGG: Vasopressin-regulated water reabsorption	-0,57	0,594	3	43
WP: Endoderm Differentiation	-0,57	0,561	11	136
KEGG: Other glycan degradation	-0,57	0,751	1	18
WP: Cell Differentiation - Index expanded	-0,57	0,582	1	18
WP: Inhibition of exosome biogenesis and secretion by Manumycin A in CRPC cells	-0,57	0,75	1	18
Reactome: Pre-NOTCH Expression and Processing	-0,58	0,565	5	67
KEGG: Propanoate metabolism	-0,58	0,559	2	31
WP: Fluoropyrimidine Activity	-0,58	0,562	2	31
WP: Gastric Cancer Network 2	-0,58	0,598	2	31
Reactome: L1CAM interactions	-0,6	0,579	7	91
KEGG: VEGF signaling pathway	-0,61	0,57	4	56
WP: Proteasome Degradation	-0,61	0,515	4	56
KEGG: Sphingolipid metabolism	-0,61	0,577	3	44
KEGG: Proteasome	-0,61	0,493	3	44
KEGG: Tight junction	-0,63	0,529	13	161
KEGG: Intestinal immune network for IgA production	-0,63	0,523	2	32
Reactome: Striated Muscle Contraction	-0,63	0,521	2	32
WP: Nuclear Receptors in Lipid Metabolism and Toxicity	-0,63	0,544	2	32
KEGG: Arginine biosynthesis	-0,63	0,49	1	19
WP: Apoptosis Modulation by HSP70	-0,63	0,653	1	19
WP: Ras Signaling	-0,65	0,523	14	173
KEGG: Long-term depression	-0,65	0,546	4	57
Reactome: Immunoregulatory interactions between a Lymphoid and a non-Lymphoid cell	-0,65	0,495	5	69
Reactome: RAF/MAP kinase cascade	-0,65	0,531	17	207
Reactome: TET1,2,3 and TDG demethylate DNA	-0,65	0,638	0	4
Reactome: Melanin biosynthesis	-0,65	0,495	0	4
Reactome: NR1D1 (REV-ERBA) represses gene expression	-0,65	0,255	0	4
Reactome: Wax biosynthesis	-0,65	0,649	0	4
Reactome: Lysosomal oligosaccharide catabolism	-0,65	0,608	0	4
Reactome: Signaling by MST1	-0,65	0,667	0	4
Reactome: rRNA processing	-0,65	0,539	0	4
WP: Peroxisomal beta-oxidation of tetracosanoyl-CoA	-0,65	0,622	0	4
WP: Secretion of Hydrochloric Acid in Parietal Cells	-0,65	0,598	0	4
WP: Oxytocin signaling	-0,65	0,408	0	4
WP: eIF5A regulation in response to inhibition of the nuclear export system	-0,65	0,201	0	4
WP: miR-222 in Exercise-Induced Cardiac Growth	-0,65	0,18	0	4
WP: Polyol Pathway	-0,65	0,537	0	4
KEGG: Sphingolipid signaling pathway	-0,65	0,521	9	116
WP: NO/cGMP/PKG mediated Neuroprotection	-0,65	0,557	3	45
KEGG: Endocytosis	-0,66	0,504	18	219
Reactome: MyD88 dependent cascade initiated on endosome	-0,68	0,487	2	33
Reactome: Transcriptional regulation of pluripotent stem cells	-0,68	0,521	2	33
WP: Alpha 6 Beta 4 signaling pathway	-0,68	0,523	2	33
WP: BDNF-TrkB Signaling	-0,68	0,522	2	33
Reactome: TNFR2 non-canonical NF-kB pathway	-0,68	0,513	4	58
Reactome: NIK-->noncanonical NF-kB signaling	-0,68	0,493	4	58
Reactome: Protein folding	-0,69	0,47	7	94
Reactome: Interleukin-20 family signaling	-0,69	0,521	1	20
WP: Triacylglyceride Synthesis	-0,69	0,522	1	20
WP: Serotonin Receptor 2 and ELK-SRF/GATA4 signaling	-0,69	0,622	1	20
KEGG: N-Glycan biosynthesis	-0,7	0,49	3	46
Reactome: Inositol phosphate metabolism	-0,7	0,49	3	46
WP: Energy Metabolism	-0,7	0,453	3	46
Reactome: Beta-catenin independent WNT signaling	-0,7	0,474	11	141
Reactome: Mitochondrial biogenesis	-0,71	0,502	5	71
Reactome: Regulation of mRNA stability by proteins that bind AU-rich elements	-0,71	0,473	6	83
WP: Androgen receptor signaling pathway	-0,71	0,485	6	83
Reactome: Integration of energy metabolism	-0,72	0,45	8	107
KEGG: Phenylalanine, tyrosine and tryptophan biosynthesis	-0,73	0,47	0	5
Reactome: Transmission across Electrical Synapses	-0,73	0,446	0	5
Reactome: FasL/ CD95L signaling	-0,73	0,46	0	5

Reactome: Protein repair	-0,73	0,571	0	5
Reactome: tRNA processing in the mitochondrion	-0,73	0,454	0	5
Reactome: MTF1 activates gene expression	-0,73	0,402	0	5
WP: exRNA mechanism of action and biogenesis	-0,73	0,235	0	5
WP: TCA Cycle Nutrient Utilization and Invasiveness of Ovarian Cancer	-0,73	0,356	0	5
WP: Synthesis and Degradation of Ketone Bodies	-0,73	0,398	0	5
WP: Dual hijack model of Vif in HIV infection	-0,73	0,3	0	5
WP: miR-517 relationship with ARCN1 and USP1	-0,73	0,204	0	5
WP: Lamin A-processing pathway	-0,73	0,233	0	5
WP: Catalytic cycle of mammalian Flavin-containing MonoOxygenases (FMOs)	-0,73	0,441	0	5
Reactome: Glycerophospholipid biosynthesis	-0,73	0,478	9	119
Reactome: DNA Damage Bypass	-0,74	0,437	3	47
KEGG: Renin-angiotensin system	-0,74	0,506	1	21
WP: NAD+ biosynthetic pathways	-0,74	0,468	1	21
KEGG: Inositol phosphate metabolism	-0,75	0,478	5	72
KEGG: Gastric acid secretion	-0,75	0,447	5	72
WP: GPCRs, Other	-0,75	0,446	5	72
Reactome: G alpha (i) signalling events	-0,76	0,448	18	224
Reactome: Apoptotic execution phase	-0,77	0,411	3	48
WP: Translation Factors	-0,77	0,4	3	48
Reactome: Signaling by VEGF	-0,78	0,456	7	97
Reactome: Synthesis of wybutosine at G37 of tRNA(Phe)	-0,8	0,476	0	6
Reactome: Fructose metabolism	-0,8	0,475	0	6
Reactome: Lipid particle organization	-0,8	0,479	0	6
Reactome: Choline catabolism	-0,8	0,569	0	6
Reactome: Aryl hydrocarbon receptor signalling	-0,8	0,506	0	6
Reactome: Galactose catabolism	-0,8	0,49	0	6
WP: Thyroxine (Thyroid Hormone) Production	-0,8	0,286	0	6
WP: Type III interferon signaling	-0,8	0,279	0	6
WP: miRNA Biogenesis	-0,8	0,194	0	6
WP: Gastric acid production	-0,8	0,494	0	6
WP: DDX1 as a regulatory component of the Drosha microprocessor	-0,8	0,231	0	6
WP: mir-124 predicted interactions with cell cycle and differentiation	-0,8	0,233	0	6
WP: Benzene metabolism	-0,8	0,38	0	6
WP: Somatroph axis (GH) and its relationship to dietary restriction and aging	-0,8	0,317	0	6
WP: Disorders of the Krebs cycle	-0,8	0,564	0	6
WP: Non-homologous end joining	-0,8	0,265	0	6
KEGG: Protein export	-0,8	0,527	1	22
Reactome: Interleukin-12 family signaling	-0,8	0,43	1	22
WP: Corticotropin-releasing hormone signaling pathway	-0,81	0,412	6	86
WP: Parkinsons Disease Pathway	-0,81	0,413	2	36
KEGG: Salivary secretion	-0,81	0,448	5	74
WP: Prader-Willi and Angelman Syndrome	-0,83	0,405	4	62
KEGG: Cholinergic synapse	-0,84	0,41	8	111
KEGG: Autophagy	-0,84	0,401	9	123
KEGG: GABAergic synapse	-0,84	0,425	6	87
KEGG: Glycosylphosphatidylinositol (GPI)-anchor biosynthesis	-0,85	0,505	1	23
KEGG: Folate biosynthesis	-0,85	0,413	1	23
Reactome: Incretin synthesis, secretion, and inactivation	-0,85	0,428	1	23
WP: Gastric Cancer Network 1	-0,85	0,517	1	23
WP: Photodynamic therapy-induced NFE2L2 (NRF2) survival signaling	-0,85	0,491	1	23
Reactome: HSP90 chaperone cycle for steroid hormone receptors (SHR)	-0,85	0,422	2	37
Reactome: Uptake and function of anthrax toxins	-0,86	0,386	0	7
Reactome: Interleukin-17 signaling	-0,86	0,299	0	7
Reactome: TYSND1 cleaves peroxisomal proteins	-0,86	0,206	0	7
WP: Pentose Phosphate Pathway	-0,86	0,374	0	7
WP: Glial Cell Differentiation	-0,86	0,195	0	7
WP: EV release from cardiac cells and their functional effects	-0,86	0,253	0	7
WP: MicroRNA for Targeting Cancer Growth and Vascularization in Glioblastoma	-0,86	0,209	0	7
WP: Mevalonate pathway	-0,86	0,305	0	7
WP: Pyrimidine metabolism and related diseases	-0,86	0,396	0	7
WP: Acetylcholine Synthesis	-0,86	0,26	0	7
Reactome: ABC-family proteins mediated transport	-0,86	0,388	7	100
WP: Leptin signaling pathway	-0,88	0,384	5	76
KEGG: Glycerolipid metabolism	-0,89	0,39	3	51
Reactome: Other interleukin signaling	-0,9	0,368	1	24
Reactome: SUMOylation of intracellular receptors	-0,9	0,356	1	24
Reactome: MAP kinase activation	-0,9	0,359	2	38
WP: Nuclear Receptors	-0,9	0,391	2	38
KEGG: GnRH signaling pathway	-0,9	0,361	6	89
KEGG: Pancreatic secretion	-0,9	0,39	6	89
Reactome: Cellular response to heat stress	-0,9	0,352	6	89
WP: Alzheimers Disease	-0,91	0,399	5	77
WP: Pyrimidine metabolism	-0,91	0,381	5	77

Reactome: Interleukin-1 family signaling	-0,92	0,393	10	138
KEGG: Riboflavin metabolism	-0,92	0,424	0	8
KEGG: Sulfur relay system	-0,92	0,264	0	8
Reactome: DNA Damage Reversal	-0,92	0,359	0	8
Reactome: mRNA Editing	-0,92	0,421	0	8
Reactome: Erythrocytes take up oxygen and release carbon dioxide	-0,92	0,434	0	8
Reactome: Ubiquinol biosynthesis	-0,92	0,387	0	8
Reactome: Base-Excision Repair, AP Site Formation	-0,92	0,372	0	8
Reactome: tRNA modification in the mitochondrion	-0,92	0,351	0	8
WP: TFs Regulate miRNAs related to cardiac hypertrophy	-0,92	0,214	0	8
WP: Metabolism of Spingolipids in ER and Golgi apparatus	-0,92	0,358	0	8
Reactome: Regulation of Apoptosis	-0,92	0,359	3	52
KEGG: Pyrimidine metabolism	-0,93	0,341	6	90
WP: G1 to S cell cycle control	-0,93	0,391	4	65
Reactome: Bile acid and bile salt metabolism	-0,94	0,378	2	39
Reactome: TNF signaling	-0,94	0,349	2	39
Reactome: Fc epsilon receptor (FCERI) signaling	-0,94	0,336	9	127
KEGG: Human cytomegalovirus infection	-0,94	0,355	15	198
KEGG: Glycosphingolipid biosynthesis	-0,94	0,4	1	25
Reactome: Signaling by ROBO receptors	-0,95	0,342	16	210
WP: AGE/RAGE pathway	-0,96	0,343	4	66
Reactome: Signal regulatory protein family interactions	-0,97	0,408	0	9
Reactome: Abacavir transport and metabolism	-0,97	0,346	0	9
Reactome: Neurotransmitter receptors and postsynaptic signal transmission	-0,97	0,335	0	9
Reactome: RHO GTPases Activate Rhotekin and Rhophilins	-0,97	0,384	0	9
Reactome: HDR through MMEJ (alt-NHEJ)	-0,97	0,366	0	9
Reactome: Mitochondrial calcium ion transport	-0,97	0,324	0	9
Reactome: Interleukin-9 signaling	-0,97	0,399	0	9
WP: Cytosine methylation	-0,97	0,229	0	9
WP: ATR Signaling	-0,97	0,201	0	9
WP: Mismatch repair	-0,97	0,207	0	9
KEGG: Serotonergic synapse	-0,97	0,338	7	104
Reactome: Kinesins	-0,98	0,341	2	40
Reactome: Signaling by Insulin receptor	-0,98	0,329	2	40
KEGG: Butanoate metabolism	-0,99	0,314	1	26
WP: EPO Receptor Signaling	-0,99	0,321	1	26
WP: MET in type 1 papillary renal cell carcinoma	-1	0,309	3	54
KEGG: Phosphatidylinositol signaling system	-1,01	0,337	6	93
Reactome: Signaling by ERBB4	-1,02	0,312	2	41
Reactome: Regulation of TP53 Expression and Degradation	-1,02	0,321	2	41
WP: Cell Cycle	-1,02	0,312	8	118
KEGG: Synthesis and degradation of ketone bodies	-1,03	0,16	0	10
Reactome: Platelet Adhesion to exposed collagen	-1,03	0,295	0	10
Reactome: Neurotoxicity of clostridium toxins	-1,03	0,306	0	10
Reactome: Mitochondrial iron-sulfur cluster biogenesis	-1,03	0,369	0	10
Reactome: SUMOylation	-1,03	0,296	0	10
Reactome: Caspase activation via Dependence Receptors in the absence of ligand	-1,03	0,273	0	10
WP: Interleukin-1 Induced Activation of NF-kappa-B	-1,03	0,197	0	10
WP: Leptin and adiponectin	-1,03	0,188	0	10
WP: IL-1 signaling pathway	-1,03	0,319	3	55
Reactome: mTOR signalling	-1,03	0,289	1	27
Reactome: Interleukin-3, Interleukin-5 and GM-CSF signaling	-1,05	0,282	2	42
Reactome: Histidine, lysine, phenylalanine, tyrosine, proline and tryptophan catabolism	-1,05	0,33	2	42
Reactome: TBC/RABGAPs	-1,05	0,292	2	42
KEGG: Peroxisome	-1,06	0,299	5	82
KEGG: RNA polymerase	-1,07	0,317	1	28
Reactome: Gap junction trafficking and regulation	-1,07	0,254	1	28
Reactome: Cristae formation	-1,07	0,334	1	28
WP: TLR4 Signaling and Tolerance	-1,07	0,315	1	28
WP: Lipid Metabolism Pathway	-1,07	0,35	1	28
KEGG: Ubiquinone and other terpenoid-quinone biosynthesis	-1,08	0,28	0	11
KEGG: Non-homologous end-joining	-1,08	0,123	0	11
Reactome: RHO GTPases activate KTN1	-1,08	0,144	0	11
Reactome: Vitamin D (calciferol) metabolism	-1,08	0,317	0	11
Reactome: SUMOylation of immune response proteins	-1,08	0,2	0	11
WP: RalA downstream regulated genes	-1,08	0,127	0	11
WP: PTF1A related regulatory pathway	-1,08	0,127	0	11
KEGG: B cell receptor signaling pathway	-1,09	0,253	4	70
Reactome: Degradation of beta-catenin by the destruction complex	-1,09	0,278	5	83
Reactome: TP53 Regulates Metabolic Genes	-1,09	0,272	5	83
WP: Integrated Cancer Pathway	-1,09	0,314	2	43
Reactome: Endosomal Sorting Complex Required For Transport (ESCRT)	-1,12	0,304	1	29
KEGG: Cell cycle	-1,12	0,29	8	122
Reactome: Presynaptic depolarization and calcium channel opening	-1,12	0,12	0	12

Reactome: Rap1 signalling	-1,12	0,299	0	12
Reactome: Erythrocytes take up carbon dioxide and release oxygen	-1,12	0,318	0	12
Reactome: RHO GTPases Activate NADPH Oxidases	-1,12	0,303	0	12
Reactome: Protein methylation	-1,12	0,193	0	12
Reactome: Processing of SMDT1	-1,12	0,215	0	12
WP: Homologous recombination	-1,12	0,12	0	12
WP: Cell-type Dependent Selectivity of CCK2R Signaling	-1,12	0,199	0	12
WP: MAPK and NFkB Signalling Pathways Inhibited by Yersinia YopJ	-1,12	0,139	0	12
Reactome: G alpha (z) signalling events	-1,13	0,257	2	44
Reactome: Cytosolic sensors of pathogen-associated DNA	-1,13	0,26	3	58
Reactome: Regulation of RUNX2 expression and activity	-1,15	0,243	4	72
KEGG: Autophagy	-1,16	0,229	1	30
Reactome: Host Interactions with Influenza Factors	-1,16	0,227	1	30
Reactome: RAB geranylgeranylation	-1,16	0,231	3	59
Reactome: Cytosolic iron-sulfur cluster assembly	-1,17	0,174	0	13
Reactome: Mitotic Telophase/Cytokinesis	-1,17	0,167	0	13
Reactome: Receptor-type tyrosine-protein phosphatases	-1,17	0,148	0	13
Reactome: Interleukin-15 signaling	-1,17	0,261	0	13
WP: MFAP5-mediated ovarian cancer cell motility and invasiveness	-1,17	0,145	0	13
WP: Disorders of Folate Metabolism and Transport	-1,17	0,295	0	13
Reactome: Hedgehog 'on' state	-1,18	0,242	5	86
Reactome: S Phase	-1,18	0,259	6	99
Reactome: Hedgehog 'off' state	-1,18	0,239	6	99
WP: Allograft Rejection	-1,18	0,229	4	73
WP: Ovarian Infertility Genes	-1,2	0,173	1	31
WP: Monoamine Transport	-1,2	0,268	1	31
Reactome: Potassium Channels	-1,2	0,225	3	60
Reactome: Selenoamino acid metabolism	-1,21	0,218	7	113
Reactome: Regulation of cholesterol biosynthesis by SREBP (SREBF)	-1,21	0,235	0	14
Reactome: Regulation of TP53 Activity through Association with Co-factors	-1,21	0,219	0	14
Reactome: Pentose phosphate pathway	-1,21	0,225	0	14
KEGG: Retrograde endocannabinoid signaling	-1,23	0,213	9	139
Reactome: Transcriptional regulation by the AP-2 (TFAP2) family of transcription factors	-1,23	0,22	2	47
Reactome: Fatty acyl-CoA biosynthesis	-1,23	0,197	1	32
Reactome: Regulation of DNA replication	-1,24	0,229	4	75
KEGG: Ascorbate and aldarate metabolism	-1,26	0,185	0	15
Reactome: Mismatch Repair	-1,26	0,206	0	15
Reactome: RUNX1 and FOXP3 control the development of regulatory T lymphocytes (Tregs)	-1,26	0,246	0	15
WP: Regulation of sister chromatid separation at the metaphase-anaphase transition	-1,26	0,137	0	15
WP: GPCRs, Class C Metabotropic glutamate, pheromone	-1,26	0,117	0	15
WP: T-Cell antigen Receptor (TCR) Signaling Pathway	-1,26	0,211	5	89
KEGG: Renin secretion	-1,26	0,206	3	62
Reactome: Mitochondrial protein import	-1,26	0,225	3	62
Reactome: DNA Double Strand Break Response	-1,27	0,2	2	48
Reactome: Clathrin-mediated endocytosis	-1,28	0,181	6	103
Reactome: YAP1- and WWTR1 (TAZ)-stimulated gene expression	-1,3	0,133	0	16
WP: Interactome of polycomb repressive complex 2 (PRC2)	-1,3	0,131	0	16
WP: NAD+ metabolism	-1,3	0,119	0	16
KEGG: Fatty acid metabolism	-1,3	0,189	2	49
Reactome: RHO GTPases Activate WASPs and WAVEs	-1,31	0,178	1	34
Reactome: Fanconi Anemia Pathway	-1,31	0,149	1	34
WP: GABA receptor Signaling	-1,31	0,243	1	34
WP: Integrated Breast Cancer Pathway	-1,31	0,187	10	155
Reactome: DNA Damage/Telomere Stress Induced Senescence	-1,33	0,168	2	50
WP: Synaptic Vesicle Pathway	-1,33	0,209	2	50
Reactome: Regulation of TP53 Activity through Methylation	-1,34	0,17	0	17
Reactome: Synaptic adhesion-like molecules	-1,34	0,139	0	17
KEGG: DNA replication	-1,35	0,119	1	35
Reactome: COPII-mediated vesicle transport	-1,35	0,179	3	65
Reactome: GABA synthesis, release, reuptake and degradation	-1,38	0,183	0	18
Reactome: RORA activates gene expression	-1,38	0,095	0	18
Reactome: SALM protein interactions at the synapse	-1,38	0,146	0	18
Reactome: SUMOylation of transcription factors	-1,38	0,147	0	18
Reactome: Digestion	-1,38	0,16	0	18
WP: Ganglio Sphingolipid Metabolism	-1,38	0,104	0	18
WP: TCA Cycle (aka Krebs or citric acid cycle)	-1,38	0,161	0	18
KEGG: Homologous recombination	-1,38	0,124	1	36
KEGG: Lysosome	-1,38	0,176	7	120
Reactome: Cardiac conduction	-1,38	0,172	8	133
Reactome: Deadenylation-dependent mRNA decay	-1,4	0,173	2	52
KEGG: Cardiac muscle contraction	-1,41	0,154	3	67
Reactome: Listeria monocytogenes entry into host cells	-1,42	0,155	0	19
Reactome: Class C/3 (Metabotropic glutamate/pheromone receptors)	-1,42	0,153	0	19
WP: Serotonin Receptor 4/6/7 and NR3C Signaling	-1,42	0,101	0	19

Reactome: Transcriptional regulation by RUNX1	-1,44	0,148	10	161
Reactome: SUMOylation of RNA binding proteins	-1,45	0,14	1	38
WP: ATM Signaling Pathway	-1,45	0,103	1	38
Reactome: POU5F1 (OCT4), SOX2, NANOG activate genes related to proliferation	-1,45	0,098	0	20
WP: Nicotine Activity on Dopaminergic Neurons	-1,45	0,087	0	20
WP: Glycerophospholipid Biosynthetic Pathway	-1,45	0,131	0	20
Reactome: M/G1 Transition	-1,46	0,131	4	83
Reactome: Transcriptional Regulation by MECP2	-1,46	0,148	4	83
Reactome: DNA Replication Pre-Initiation	-1,46	0,131	4	83
WP: Sterol Regulatory Element-Binding Proteins (SREBP) signalling	-1,47	0,144	3	69
WP: B Cell Receptor Signaling Pathway	-1,47	0,147	5	97
KEGG: Dopaminergic synapse	-1,48	0,138	7	124
KEGG: Terpenoid backbone biosynthesis	-1,49	0,137	0	21
Reactome: GABA receptor activation	-1,49	0,127	2	55
Reactome: trans-Golgi Network Vesicle Budding	-1,5	0,141	3	70
Reactome: Neurexins and neuroligins	-1,52	0,124	2	56
KEGG: Mismatch repair	-1,52	0,093	0	22
Reactome: Peroxisomal lipid metabolism	-1,52	0,126	0	22
Reactome: DNA methylation	-1,52	0,103	0	22
Reactome: C-type lectin receptors (CLRs)	-1,55	0,105	7	127
Reactome: RNA Polymerase III Transcription	-1,55	0,111	1	41
WP: DNA Replication	-1,55	0,08	1	41
Reactome: Activation of gene expression by SREBF (SREBP)	-1,55	0,119	2	57
Reactome: Activated PKN1 stimulates transcription of AR (androgen receptor) regulated genes KLK2 and KLK3	-1,56	0,133	0	23
WP: MTHFR deficiency	-1,56	0,088	0	23
WP: The effect of progerin on the involved genes in Hutchinson-Gilford Progeria Syndrome	-1,56	0,076	0	23
WP: Retinoblastoma Gene in Cancer	-1,57	0,098	4	87
WP: Heart Development	-1,58	0,074	1	42
Reactome: Mitophagy	-1,59	0,08	0	24
Reactome: Peroxisomal protein import	-1,61	0,095	2	59
Reactome: TCR signaling	-1,62	0,121	5	103
Reactome: Fertilization	-1,62	0,065	0	25
Reactome: MyD88 cascade initiated on plasma membrane	-1,62	0,09	0	25
Reactome: TP53 Regulates Transcription of Cell Cycle Genes	-1,64	0,1	2	60
Reactome: RUNX1 regulates genes involved in megakaryocyte differentiation and platelet function	-1,64	0,127	2	60
KEGG: Mucin type O-glycan biosynthesis	-1,66	0,074	0	26
KEGG: Phototransduction	-1,66	0,07	0	26
KEGG: Collecting duct acid secretion	-1,66	0,068	0	26
Reactome: SIRT1 negatively regulates rRNA expression	-1,66	0,064	0	26
Reactome: Integrin alphaIIb beta3 signaling	-1,66	0,08	0	26
WP: T-Cell antigen Receptor (TCR) pathway during Staphylococcus aureus infection	-1,66	0,067	2	61
KEGG: Natural killer cell mediated cytotoxicity	-1,67	0,083	4	91
KEGG: Oxytocin signaling pathway	-1,67	0,084	8	146
Reactome: Processing of Capped Intronless Pre-mRNA	-1,69	0,062	0	27
WP: Sphingolipid Metabolism	-1,69	0,085	0	27
Reactome: Asparagine N-linked glycosylation	-1,69	0,1	6	120
KEGG: Fanconi anemia pathway	-1,7	0,069	1	46
WP: Calcium Regulation in the Cardiac Cell	-1,71	0,086	8	148
Reactome: Signaling by the B Cell Receptor (BCR)	-1,72	0,069	5	107
Reactome: Regulation of TP53 Activity through Acetylation	-1,72	0,077	0	28
Reactome: G-protein beta:gamma signalling	-1,72	0,06	0	28
Reactome: SUMOylation of SUMOylation proteins	-1,72	0,058	0	28
WP: T-Cell Receptor and Co-stimulatory Signaling	-1,72	0,069	0	28
KEGG: Long-term potentiation	-1,72	0,061	2	63
Reactome: XBP1(S) activates chaperone genes	-1,72	0,103	4	93
Reactome: B-WICH complex positively regulates rRNA expression	-1,73	0,08	1	47
Reactome: Cargo recognition for clathrin-mediated endocytosis	-1,74	0,071	3	79
Reactome: mRNA Capping	-1,75	0,068	0	29
Reactome: Energy dependent regulation of mTOR by LKB1-AMPK	-1,75	0,05	0	29
Reactome: Protein ubiquitination	-1,75	0,062	0	29
WP: Initiation of transcription and translation elongation at the HIV-1 LTR	-1,75	0,064	0	29
KEGG: Valine, leucine and isoleucine degradation	-1,76	0,077	1	48
Reactome: Meiotic synapsis	-1,76	0,068	1	48
Reactome: tRNA processing in the nucleus	-1,76	0,076	1	48
Reactome: SUMOylation of chromatin organization proteins	-1,76	0,076	1	48
KEGG: Adrenergic signaling in cardiomyocytes	-1,77	0,071	7	137
Reactome: PRC2 methylates histones and DNA	-1,78	0,055	0	30
Reactome: Activation of kainate receptors upon glutamate binding	-1,78	0,051	0	30
WP: Tumor suppressor activity of SMARCB1	-1,78	0,063	0	30
Reactome: tRNA modification in the nucleus and cytosol	-1,81	0,071	0	31
Reactome: SUMOylation of ubiquitinylation proteins	-1,81	0,055	0	31
KEGG: Taste transduction	-1,83	0,07	2	67
Reactome: ROS, RNS production in phagocytes	-1,84	0,04	0	32

KEGG: Protein processing in endoplasmic reticulum	-1,84	0,06	8	154
KEGG: Human immunodeficiency virus 1 infection	-1,84	0,077	10	181
Reactome: MHC class II antigen presentation	-1,86	0,059	3	84
KEGG: Base excision repair	-1,87	0,052	0	33
KEGG: SNARE interactions in vesicular transport	-1,87	0,043	0	33
WP: Oxidative phosphorylation	-1,87	0,042	1	52
Reactome: Class I MHC mediated antigen processing & presentation	-1,87	0,064	23	348
KEGG: T cell receptor signaling pathway	-1,89	0,054	4	100
Reactome: Regulation of mitotic cell cycle	-1,89	0,053	3	85
KEGG: Ubiquitin mediated proteolysis	-1,89	0,051	6	129
Reactome: ERCC6 (CSB) and EHMT2 (G9a) positively regulate rRNA expression	-1,89	0,037	0	34
Reactome: Telomere Maintenance	-1,9	0,06	1	53
Reactome: Oncogene Induced Senescence	-1,92	0,049	0	35
Reactome: RNA Polymerase I Transcription	-1,92	0,064	1	54
WP: DNA IR-Double Strand Breaks (DSBs) and cellular response via ATM	-1,92	0,04	1	54
Reactome: Resolution of Abasic Sites (AP sites)	-1,95	0,053	0	36
Reactome: Keratinization	-1,95	0,049	1	55
Reactome: PTEN Regulation	-1,96	0,041	6	132
WP: Electron Transport Chain (OXPHOS system in mitochondria)	-1,96	0,054	3	88
Reactome: Host Interactions of HIV factors	-1,96	0,044	5	118
WP: Histone Modifications	-1,98	0,033	0	37
Reactome: Oxidative Stress Induced Senescence	-1,98	0,039	3	89
Reactome: Nonhomologous End-Joining (NHEJ)	-2,03	0,041	0	39
Reactome: SUMOylation of DNA replication proteins	-2,03	0,029	0	39
WP: DNA IR-damage and cellular response via ATR	-2,05	0,026	2	76
Reactome: Regulation of beta-cell development	-2,05	0,029	1	59
Reactome: Nucleosome assembly	-2,05	0,031	0	40
WP: Eukaryotic Transcription Initiation	-2,05	0,042	0	40
Reactome: Mitotic G1-G1/S phases	-2,07	0,031	8	166
KEGG: Basal transcription factors	-2,08	0,024	0	41
Reactome: Ion channel transport	-2,09	0,036	8	167
Reactome: Meiotic recombination	-2,11	0,033	0	42
KEGG: Oocyte meiosis	-2,11	0,037	4	110
KEGG: Synaptic vesicle cycle	-2,13	0,038	1	62
KEGG: Nucleotide excision repair	-2,13	0,027	0	43
WP: ATM Signaling Network in Development and Disease	-2,13	0,027	0	43
Reactome: NoRC negatively regulates rRNA expression	-2,15	0,027	1	63
Reactome: Transcriptional Regulation by E2F6	-2,15	0,031	0	44
Reactome: Neddylation	-2,16	0,044	12	225
Reactome: COPI-mediated anterograde transport	-2,17	0,027	2	81
Reactome: Macroautophagy	-2,17	0,028	1	64
Reactome: Metabolism of non-coding RNA	-2,2	0,027	0	46
Reactome: E3 ubiquitin ligases ubiquitinate target proteins	-2,23	0,021	0	47
Reactome: SUMOylation of DNA damage response and repair proteins	-2,24	0,024	1	67
Reactome: RNA polymerase II transcribes snRNA genes	-2,24	0,02	1	67
WP: Mitochondrial complex I assembly model OXPHOS system	-2,25	0,023	0	48
Reactome: Deubiquitination	-2,26	0,035	12	231
Reactome: Synthesis of DNA	-2,26	0,024	4	117
Reactome: Mitotic Prophase	-2,26	0,015	2	85
Reactome: Assembly of the primary cilium	-2,26	0,029	8	176
Reactome: Cilium Assembly	-2,26	0,031	8	176
Reactome: Mitotic G2-G2/M phases	-2,3	0,022	8	178
Reactome: TP53 Regulates Transcription of DNA Repair Genes	-2,31	0,016	1	70
KEGG: Thermogenesis	-2,31	0,018	10	207
KEGG: Lysine degradation	-2,32	0,022	0	51
Reactome: Mitochondrial translation	-2,37	0,027	2	90
Reactome: Activation of anterior HOX genes in hindbrain development during early embryogenesis	-2,37	0,012	2	90
KEGG: RNA transport	-2,52	0,007	5	146
KEGG: mRNA surveillance pathway	-2,53	0,013	1	80
Reactome: RAB GEFs exchange GTP for GDP on RABs	-2,59	0,012	1	83
WP: Cytoplasmic Ribosomal Proteins	-2,65	0,007	1	86
Reactome: Regulation of TP53 Activity through Phosphorylation	-2,71	0,015	1	89
Reactome: Eukaryotic Translation Elongation	-2,74	0,007	1	91
Reactome: RHO GTPases Activate Formins	-2,78	0,005	2	111
KEGG: Oxidative phosphorylation	-2,85	0,005	2	115
Reactome: Eukaryotic Translation Initiation	-2,87	0,005	2	116
Reactome: HDR through Homologous Recombination (HRR) or Single Strand Annealing (SSA)	-2,95	0,003	1	102
Reactome: Gene Silencing by RNA	-3	0,002	0	85
Reactome: Respiratory electron transport, ATP synthesis by chemiosmotic coupling, and heat production by uncoupling proteins.	-3,05	0,003	1	108
Reactome: Eukaryotic Translation Termination	-3,09	0,001	0	90
Reactome: HIV Life Cycle	-3,13	0,003	2	131
Reactome: Mitotic Prometaphase	-3,15	0,005	4	167
Reactome: Intra-Golgi and retrograde Golgi-to-ER traffic	-3,15	0,003	4	167

Reactome: Mitotic Metaphase and Anaphase	-3,18	0,003	4	169
Reactome: Major pathway of rRNA processing in the nucleolus and cytosol	-3,18	0,002	3	152
KEGG: Ribosome	-3,38	0	1	128
Reactome: Nucleotide Excision Repair	-3,38	0,002	0	108
Reactome: SRP-dependent cotranslational protein targeting to membrane	-3,4	0	0	109
Reactome: Nonsense-Mediated Decay (NMD)	-3,46	0,001	0	113
WP: mRNA Processing	-3,57	0,001	0	120
KEGG: Spliceosome	-3,58	0,001	0	121
Reactome: Influenza Life Cycle	-3,78	0	0	135
Reactome: RNA Polymerase II Transcription	-3,81	0,001	0	137
Reactome: Chromatin organization	-3,93	0	3	203
Reactome: Cell Cycle Checkpoints	-4,13	0,002	5	254
Reactome: G alpha (s) signalling events	-4,17	0	10	340
Reactome: Generic Transcription Pathway	-4,21	0	4	243
KEGG: Olfactory transduction	-4,58	0	3	256
Reactome: Processing of Capped Intron-Containing Pre-mRNA	-4,67	0	1	225
WP: Aripiprazole Metabolic Pathway	NaN	0	0	0
WP: Human metabolism overview	NaN	0	0	0
WP: Biochemical Pathways Part I	NaN	0	0	0
WP: Biosynthesis and regeneration of tetrahydrobiopterin (BH4) and catabolism of phenylalanine, including diseases	NaN	0	0	0
WP: GHB metabolic pathway	NaN	0	0	0
WP: Mevalonate arm of cholesterol biosynthesis pathway	NaN	0	0	0
WP: Phosphatidylcholine catabolism	NaN	0	0	0
WP: Amino acid conjugation	NaN	0	0	0

**Positive: the number of genes on the pathway that pass the statistical criteria (absolute logFC > 0.58 and p-value < 0.05)*
**Measured: the number of genes on the pathway that were measured in the dataset*
WP: WikiPathways
The red line indicates the end of the significantly changed pathways for this analysis
