# Supplementary material for: The Molecular Processes in the Trabecular Meshwork After Exposure to Corticosteroids and in Corticosteroid-Induced Ocular Hypertension
Source: Invest Ophthalmol Vis Sci. 2020 Apr 18;61(4):24. doi: 10.1167/iovs.61.4.24 (PMC7401422; doi:10.1167/iovs.61.4.24)
Supplement: Supplement 4 [file iovs-61-4-24_s004.pdf]

Appendix 4. Complete pathway results of responder vs. non-responder data

Pathway

WP: Cholesterol Biosynthesis
Reactome: Cholesterol biosynthesis
Reactome: Activation of gene expression by SREBF (SREBP)
WP: Mevalonate pathway
Reactome: Interferon alpha/beta signaling
KEGG: Steroid biosynthesis
WP: Differentiation Pathway
KEGG: Terpenoid backbone biosynthesis
WP: Lung fibrosis
WP: Extracellular vesicles in the crosstalk of cardiac cells
WP: Mevalonate arm of cholesterol biosynthesis pathway with inhibitors
WP: Sterol Regulatory Element-Binding Proteins (SREBP) signalling
Reactome: Mitotic G1-G1/S phases
WP: BMP2-WNT4-FOXO1 Pathway in Human Primary Endometrial Stromal Cell Differentiation
Reactome: Regulation of Insulin-like Growth Factor (IGF) transport and uptake by Insulin-like Growth Factor Binding Proteins (IGFBPs)
Reactome: Post-translational protein phosphorylation
WP: Oxidative Stress
WP: Cardiac Progenitor Differentiation
WP: SREBF and miR33 in cholesterol and lipid homeostasis
Reactome: Interconversion of nucleotide di- and triphosphates
WP: Angiogenesis
KEGG: Gap junction
WP: Overview of nanoparticle effects
Reactome: Latent infection of Homo sapiens with Mycobacterium tuberculosis
WP: Proprotein convertase subtilisin/kexin type 9 (PCSK9) mediated LDL receptor degradation
WP: Evolocumab Mechanism
Reactome: Interleukin-10 signaling
WP: Spinal Cord Injury
Reactome: Neurotransmitter uptake and metabolism In glial cells
Reactome: Biosynthesis of DPA-derived SPMs
WP: Matrix Metalloproteinases
Reactome: Assembly of collagen fibrils and other multimeric structures
WP: Senescence and Autophagy in Cancer
WP: Steroid Biosynthesis
Reactome: Signaling by Type 1 Insulin-like Growth Factor 1 Receptor (IGF1R)
KEGG: Biosynthesis of unsaturated fatty acids
Reactome: Collagen chain trimerization
Reactome: Extracellular matrix organization
WP: Gastric Cancer Network 1
WP: Fatty Acid Biosynthesis
KEGG: Glutathione metabolism
WP: Glucocorticoid and Mineralcorticoid Metabolism
WP: LncRNA involvement in canonical Wnt signaling and colorectal cancer
WP: ncRNAs involved in Wnt signaling in hepatocellular carcinoma
WP: Endochondral Ossification

Reactome: Signaling by PDGF
WP: Photodynamic therapy-induced NF-kB survival signaling
WP: Prader-Willi and Angelman Syndrome
WP: Vitamin D Receptor Pathway
Reactome: Interleukin-4 and Interleukin-13 signaling
WP: Simplified Interaction Map Between LOXL4 and Oxidative Stress Pathway
Reactome: Oncogene Induced Senescence
WP: Retinoblastoma Gene in Cancer
WP: G1 to S cell cycle control
KEGG: Drug metabolism
WP: Pyrimidine metabolism
WP: Nuclear Receptors Meta-Pathway
Reactome: Regulation of lipid metabolism by Peroxisome proliferator-activated receptor alpha (PPARalpha)
WP: Statin Pathway
Reactome: Collagen degradation
WP: Benzene metabolism
KEGG: Pyrimidine metabolism
WP: Photodynamic therapy-induced HIF-1 survival signaling
WP: Selenium Micronutrient Network
Reactome: Mitotic Prometaphase
Reactome: Dissolution of Fibrin Clot
WP: EDA Signalling in Hair Follicle Development
WP: Apoptosis-related network due to altered Notch3 in ovarian cancer
WP: Photodynamic therapy-induced AP-1 survival signaling.
Reactome: Collagen biosynthesis and modifying enzymes
Reactome: Biosynthesis of electrophilic Ï‰-3 PUFA oxo-derivatives
WP: Butyrate-induced histone acetylation
WP: Hormonal control of Pubertal Growth Spurt
KEGG: Cell cycle
WP: Pathogenic Escherichia coli infection
WP: LncRNA-mediated mechanisms of therapeutic resistance
Reactome: Elastic fibre formation
WP: The human immune response to tuberculosis
WP: Phytochemical activity on NRF2 transcriptional activation
WP: Osteoblast Signaling
WP: TGF-beta Receptor Signaling
WP: Wnt Signaling Pathway
WP: Eicosanoid Synthesis
WP: IL1 and megakaryocytes in obesity
WP: Prostaglandin Synthesis and Regulation
WP: miRNAs involved in DNA damage response
KEGG: p53 signaling pathway
WP: Cell Cycle
KEGG: Ovarian steroidogenesis
KEGG: TGF-beta signaling pathway
WP: Signaling Pathways in Glioblastoma
Reactome: Nicotinate metabolism
Reactome: Deregulated CDK5 triggers multiple neurodegenerative pathways in Alzheimer's disease models
WP: Hair Follicle Development: Cytodifferentiation (Part 3 of 3)
Reactome: Senescence-Associated Secretory Phenotype (SASP)

Reactome: Cell Cycle Checkpoints
Reactome: Degradation of the extracellular matrix
Reactome: RHO GTPases Activate Formins
WP: Glutathione metabolism
WP: Composition of Lipid Particles
KEGG: Fatty acid metabolism
WP: Vitamin B12 Metabolism
KEGG: Wnt signaling pathway
Reactome: Integrin cell surface interactions
WP: NRF2 pathway
Reactome: RHO GTPases activate CIT
WP: TGF-B Signaling in Thyroid Cells for Epithelial-Mesenchymal Transition
KEGG: Hepatocellular carcinoma
Reactome: Intestinal absorption
WP: Amino acid conjugation of benzoic acid
KEGG: Hematopoietic cell lineage
Reactome: Nucleosome assembly
KEGG: Synthesis and degradation of ketone bodies
WP: Liver X Receptor Pathway
Reactome: Amino acid synthesis and interconversion (transamination)
WP: BMP Signaling Pathway in Eyelid Development
WP: DNA Replication
Reactome: Retinoid metabolism and transport
KEGG: Histidine metabolism
KEGG: Other types of O-glycan biosynthesis
WP: Imatinib and Chronic Myeloid Leukemia
Reactome: Carboxyterminal post-translational modifications of tubulin
WP: Gastric Cancer Network 2
WP: Factors and pathways affecting insulin-like growth factor (IGF1)-Akt signaling
WP: Type II interferon signaling (IFNG)
Reactome: Peptide hormone biosynthesis
KEGG: Protein digestion and absorption
WP: Apoptosis
WP: Primary Focal Segmental Glomerulosclerosis FSGS
KEGG: Arachidonic acid metabolism
Reactome: RAF-independent MAPK1/3 activation
Reactome: Nucleotide salvage
Reactome: Fatty acyl-CoA biosynthesis
WP: Sudden Infant Death Syndrome (SIDS) Susceptibility Pathways
KEGG: Mineral absorption
WP: Pathways in clear cell renal cell carcinoma
KEGG: FoxO signaling pathway
WP: Adipogenesis
WP: Melatonin metabolism and effects
WP: ESC Pluripotency Pathways
WP: NRF2-ARE regulation
Reactome: Advanced glycosylation endproduct receptor signaling
Reactome: alpha-linolenic (omega3) and linoleic (omega6) acid metabolism
Reactome: Biosynthesis of DHA-derived SPMs
Reactome: Melanin biosynthesis

Reactome: NR1D1 (REV-ERBA) represses gene expression
WP: miR-222 in Exercise-Induced Cardiac Growth
WP: Serotonin Receptor 2 and STAT3 Signaling
WP: Apoptosis Modulation and Signaling
Reactome: Detoxification of Reactive Oxygen Species
WP: Copper homeostasis
WP: Hepatitis C and Hepatocellular Carcinoma
WP: Wnt Signaling in Kidney Disease
KEGG: Cushing syndrome
Reactome: Regulation of DNA replication
KEGG: PI3K-Akt signaling pathway
WP: Osteopontin Signaling
WP: Genes targeted by miRNAs in adipocytes
WP: ncRNAs involved in STAT3 signaling in hepatocellular carcinoma
KEGG: Phagosome
Reactome: Circadian Clock
Reactome: Binding and Uptake of Ligands by Scavenger Receptors
Reactome: Transcriptional regulation by RUNX3
KEGG: Cytokine-cytokine receptor interaction
WP: Folate Metabolism
WP: Parkin-Ubiquitin Proteasomal System pathway
WP: PI3K-Akt Signaling Pathway
Reactome: DNA Damage/Telomere Stress Induced Senescence
WP: Sleep regulation
WP: PPAR Alpha Pathway
Reactome: Cell junction organization
WP: Estrogen metabolism
Reactome: Protein folding
Reactome: Neutrophil degranulation
WP: Bladder Cancer
KEGG: Butanoate metabolism
Reactome: Metallothioneins bind metals
Reactome: Biosynthesis of EPA-derived SPMs
KEGG: Ferroptosis
Reactome: SUMOylation of DNA replication proteins
WP: Oxidative Damage
WP: Ferroptosis
KEGG: Focal adhesion
KEGG: HIF-1 signaling pathway
WP: Wnt Signaling Pathway and Pluripotency
Reactome: Regulation of TLR by endogenous ligand
WP: H19 action Rb-E2F1 signaling and CDK-Beta-catenin activity
WP: Regulation of sister chromatid separation at the metaphase-anaphase transition
KEGG: Hippo signaling pathway
Reactome: Activation of Matrix Metalloproteinases
WP: Aryl Hydrocarbon Receptor Pathway
KEGG: IL-17 signaling pathway
WP: Viral Acute Myocarditis
WP: TGF-beta Signaling Pathway
WP: Metabolic reprogramming in colon cancer

WP: Ectoderm Differentiation
Reactome: Metabolism of steroid hormones
Reactome: TNFs bind their physiological receptors
WP: Lipid Metabolism Pathway
KEGG: Nitrogen metabolism
WP: Osteoclast Signaling
WP: Complement and Coagulation Cascades
KEGG: Circadian rhythm
Reactome: Interferon gamma signaling
Reactome: Uptake and function of diphtheria toxin
Reactome: Aryl hydrocarbon receptor signalling
Reactome: Synthesis of Lipoxins (LX)
WP: Type III interferon signaling
WP: SCFA and skeletal muscle substrate metabolism
WP: ErbB Signaling Pathway
Reactome: Mitotic Metaphase and Anaphase
Reactome: ISG15 antiviral mechanism
WP: Regulation of Wnt/B-catenin Signaling by Small Molecule Compounds
WP: Amplification and Expansion of Oncogenic Pathways as Metastatic Traits
WP: Canonical and Non-Canonical TGF-B signaling
Reactome: Class B/2 (Secretin family receptors)
KEGG: Glyoxylate and dicarboxylate metabolism
WP: miRNA regulation of prostate cancer signaling pathways
WP: Oligodendrocyte Specification and differentiation(including remyelination), leading to Myelin Components for CNS
Reactome: Transcriptional Regulation by E2F6
WP: Exercise-induced Circadian Regulation
KEGG: JAK-STAT signaling pathway
WP: Aryl Hydrocarbon Receptor
WP: Endothelin Pathways
Reactome: TP53 Regulates Transcription of Cell Cycle Genes
WP: Focal Adhesion-PI3K-Akt-mTOR-signaling pathway
WP: Focal Adhesion
WP: Circadian rythm related genes
KEGG: Metabolic pathways
Reactome: Cell surface interactions at the vascular wall
KEGG: ECM-receptor interaction
Reactome: TRAIL signaling
Reactome: OAS antiviral response
WP: HIF1A and PPARG regulation of glycolysis
WP: Hypothetical Craniofacial Development Pathway
WP: Pyrimidine metabolism and related diseases
WP: Aflatoxin B1 metabolism
Reactome: Transcriptional regulation by the AP-2 (TFAP2) family of transcription factors
Reactome: Post-translational modification: synthesis of GPI-anchored proteins
Reactome: Synthesis, secretion, and deacylation of Ghrelin
WP: TP53 Network
WP: Nucleotide Metabolism
WP: Urea cycle and metabolism of amino groups
Reactome: Class A/1 (Rhodopsin-like receptors)

Reactome: DDX58/IFIH1-mediated induction of interferon-alpha/beta
WP: Human Complement System
Reactome: Phase I - Functionalization of compounds
WP: DNA Damage Response (only ATM dependent)
WP: DNA Damage Response
Reactome: Response to elevated platelet cytosolic Ca2+
KEGG: Alanine, aspartate and glutamate metabolism
KEGG: Melanogenesis
Reactome: Syndecan interactions
Reactome: Interleukin-20 family signaling
Reactome: Phase II - Conjugation of compounds
WP: Association Between Physico-Chemical Features and Toxicity Associated Pathways
KEGG: DNA replication
Reactome: Antimicrobial peptides
Reactome: PI3K Cascade
WP: Selenium Metabolism and Selenoproteins
Reactome: Erythrocytes take up oxygen and release carbon dioxide
WP: Caloric restriction and aging
WP: Methylation Pathways
KEGG: Ras signaling pathway
Reactome: M/G1 Transition
Reactome: DNA Replication Pre-Initiation
WP: Transcription factor regulation in adipogenesis
WP: Complement Activation
Reactome: Pre-NOTCH Expression and Processing
WP: miRNA Regulation of DNA Damage Response
WP: PPAR signaling pathway
KEGG: Fat digestion and absorption
KEGG: Signaling pathways regulating pluripotency of stem cells
Reactome: Arachidonic acid metabolism
Reactome: Transport of bile salts and organic acids, metal ions and amine compounds
KEGG: alpha-Linolenic acid metabolism
KEGG: Nicotinate and nicotinamide metabolism
Reactome: DNA methylation
WP: Methionine De Novo and Salvage Pathway
WP: Regulation of Apoptosis by Parathyroid Hormone-related Protein
WP: Mesodermal Commitment Pathway
Reactome: Formation of Fibrin Clot (Clotting Cascade)
WP: Non-genomic actions of 1,25 dihydroxyvitamin D3
KEGG: Regulation of lipolysis in adipocytes
WP: Epithelial to mesenchymal transition in colorectal cancer
Reactome: Signaling by Leptin
Reactome: Signaling by TGF-beta Receptor Complex
WP: Nanoparticle triggered autophagic cell death
WP: Photodynamic therapy-induced NFE2L2 (NRF2) survival signaling
WP: Cytokines and Inflammatory Response
Reactome: Gene and protein expression by JAK-STAT signaling after Interleukin-12 stimulation
Reactome: G alpha (q) signalling events
KEGG: Steroid hormone biosynthesis
KEGG: Fatty acid elongation

Reactome: Interleukin-6 family signaling
Reactome: SUMOylation of intracellular receptors
WP: Differentiation of white and brown adipocyte
WP: Amino Acid metabolism
Reactome: Kinesins
WP: Ebola Virus Pathway on Host
KEGG: Gastric cancer
Reactome: Platelet Aggregation (Plug Formation)
Reactome: Trafficking and processing of endosomal TLR
Reactome: POU5F1 (OCT4), SOX2, NANOG repress genes related to differentiation
WP: Valproic acid pathway
WP: Ethanol metabolism resulting in production of ROS by CYP2E1
KEGG: PPAR signaling pathway
Reactome: PIP3 activates AKT signaling
KEGG: Apoptosis
KEGG: Glycosphingolipid biosynthesis
Reactome: WNT ligand biogenesis and trafficking
WP: IL-7 Signaling Pathway
WP: Hypothesized Pathways in Pathogenesis of Cardiovascular Disease
Reactome: Signaling by Retinoic Acid
WP: TNF related weak inducer of apoptosis (TWEAK) Signaling Pathway
WP: Vitamin A and Carotenoid Metabolism
KEGG: Cellular senescence
Reactome: O-linked glycosylation
KEGG: Complement and coagulation cascades
KEGG: Mucin type O-glycan biosynthesis
Reactome: Signaling by BMP
WP: Wnt/beta-catenin Signaling Pathway in Leukemia
KEGG: Ubiquinone and other terpenoid-quinone biosynthesis
WP: Vitamin D Metabolism
WP: Gene regulatory network modelling somitogenesis
KEGG: Longevity regulating pathway
Reactome: Transcriptional regulation by RUNX2
WP: Integrated Cancer Pathway
WP: Glycolysis and Gluconeogenesis
WP: Integrin-mediated Cell Adhesion
Reactome: TAK1 activates NFkB by phosphorylation and activation of IKKs complex
KEGG: Drug metabolism
KEGG: Cholesterol metabolism
WP: Myometrial Relaxation and Contraction Pathways
KEGG: Renin secretion
Reactome: Passive transport by Aquaporins
Reactome: Erythrocytes take up carbon dioxide and release oxygen
Reactome: Reversible hydration of carbon dioxide
WP: Alanine and aspartate metabolism
WP: Bone Morphogenic Protein (BMP) Signalling and Regulation
WP: Iron metabolism in placenta
WP: Dopamine metabolism
WP: Purine metabolism
WP: Notch Signaling Pathway

Reactome: Synthesis of DNA
Reactome: TP53 Regulates Transcription of Cell Death Genes
KEGG: AMPK signaling pathway
KEGG: Metabolism of xenobiotics by cytochrome P450
Reactome: Signaling by PTK6
KEGG: ErbB signaling pathway
WP: Oncostatin M Signaling Pathway
KEGG: Apoptosis
Reactome: Nucleobase catabolism
WP: Dopaminergic Neurogenesis
Reactome: Transcriptional Regulation by MECP2
WP: MicroRNAs in cardiomyocyte hypertrophy
KEGG: Notch signaling pathway
Reactome: DNA Damage Bypass
Reactome: Intrinsic Pathway for Apoptosis
KEGG: Fatty acid biosynthesis
Reactome: Mitotic Telophase/Cytokinesis
Reactome: Signaling by Activin
Reactome: Regulated Necrosis
WP: Development of pulmonary dendritic cells and macrophage subsets
WP: Serotonin and anxiety-related events
KEGG: beta-Alanine metabolism
Reactome: Activation of kainate receptors upon glutamate binding
WP: PI3K-AKT-mTOR signaling pathway and therapeutic opportunities
WP: Development and heterogeneity of the ILC family
KEGG: Cytosolic DNA-sensing pathway
WP: Mitochondrial complex I assembly model OXPHOS system
Reactome: Regulation of mitotic cell cycle
WP: Metapathway biotransformation Phase I and II
KEGG: Propanoate metabolism
WP: Fluoropyrimidine Activity
WP: Type 2 papillary renal cell carcinoma
WP: Monoamine GPCRs
Reactome: Netrin-1 signaling
KEGG: Rap1 signaling pathway
KEGG: Glycosaminoglycan biosynthesis
Reactome: Regulation of cholesterol biosynthesis by SREBP (SREBF)
Reactome: Caspase activation via Death Receptors in the presence of ligand
Reactome: Acetylcholine binding and downstream events
WP: Glucuronidation
KEGG: Bile secretion
KEGG: TNF signaling pathway
KEGG: Tyrosine metabolism
KEGG: Intestinal immune network for IgA production
KEGG: Selenocompound metabolism
Reactome: Metabolism of nitric oxide
Reactome: Defensins
Reactome: Metabolism of Angiotensinogen to Angiotensins
Reactome: Nucleobase biosynthesis
Reactome: Regulation of RUNX1 Expression and Activity

WP: GPCRs, Class B Secretin-like
WP: Role of Osx and miRNAs in tooth development
WP: Biogenic Amine Synthesis
WP: Sulfation Biotransformation Reaction
Reactome: Oxidative Stress Induced Senescence
WP: Alpha 6 Beta 4 signaling pathway
WP: Genotoxicity pathway
KEGG: mTOR signaling pathway
KEGG: Regulation of actin cytoskeleton
Reactome: Signaling by FGFR3
Reactome: BMAL1:CLOCK,NPAS2 activates circadian gene expression
Reactome: Smooth Muscle Contraction
Reactome: Triglyceride metabolism
KEGG: Inflammatory mediator regulation of TRP channels
WP: Chromosomal and microsatellite instability in colorectal cancer
WP: Interferon type I signaling pathways
KEGG: Phenylalanine metabolism
Reactome: Metabolism of porphyrins
WP: Cori Cycle
WP: TCA Cycle and Deficiency of Pyruvate Dehydrogenase complex (PDHc)
WP: NOTCH1 regulation of human endothelial cell calcification
WP: ID signaling pathway
WP: Breast cancer pathway
WP: Cardiac Hypertrophic Response
WP: Hematopoietic Stem Cell Differentiation
Reactome: Gastrin-CREB signalling pathway via PKC and MAPK
Reactome: Aflatoxin activation and detoxification
Reactome: Cellular hexose transport
WP: Leptin Insulin Overlap
WP: Serotonin and anxiety
WP: miR-509-3p alteration of YAP1/ECM axis
Reactome: Transport of vitamins, nucleosides, and related molecules
Reactome: Gamma carboxylation, hypusine formation and arylsulfatase activation
Reactome: Signaling by FGFR4
Reactome: RET signaling
WP: Target Of Rapamycin (TOR) Signaling
WP: Striated Muscle Contraction
KEGG: RIG-I-like receptor signaling pathway
KEGG: Tryptophan metabolism
Reactome: Neurotransmitter release cycle
WP: Fibrin Complement Receptor 3 Signaling Pathway
WP: Hedgehog Signaling Pathway
Reactome: RORA activates gene expression
Reactome: Amine-derived hormones
Reactome: SUMOylation of transcription factors
WP: Farnesoid X Receptor Pathway
WP: 4-hydroxytamoxifen, Dexamethasone, and Retinoic Acids Regulation of p27 Expression
WP: RIG-I-like Receptor Signaling
KEGG: Pyruvate metabolism
WP: Nuclear Receptors

WP: ATM Signaling Pathway
WP: PDGF Pathway
Reactome: Iron uptake and transport
Reactome: Mitotic G2-G2/M phases
KEGG: Cell adhesion molecules (CAMs)
KEGG: Arginine biosynthesis
Reactome: RHO GTPases Activate ROCKs
WP: Platelet-mediated interactions with vascular and circulating cells
Reactome: S Phase
KEGG: Tight junction
KEGG: Cortisol synthesis and secretion
KEGG: Hedgehog signaling pathway
Reactome: Signaling by Insulin receptor
WP: Neural Crest Differentiation
WP: Hypertrophy Model
WP: Serotonin Receptor 2 and ELK-SRF/GATA4 signaling
KEGG: Neuroactive ligand-receptor interaction
Reactome: ESR-mediated signaling
Reactome: Transport of inorganic cations/anions and amino acids/oligopeptides
KEGG: Antigen processing and presentation
Reactome: Regulation of TP53 Expression and Degradation
WP: MECP2 and Associated Rett Syndrome
WP: Nonalcoholic fatty liver disease
WP: VEGFA-VEGFR2 Signaling Pathway
KEGG: Renin-angiotensin system
Reactome: Growth hormone receptor signaling
Reactome: RHO GTPases activate PAKs
Reactome: Signaling by NTRK2 (TRKB)
WP: miRNA targets in ECM and membrane receptors
WP: PI3K/AKT/mTOR - VitD3 Signalling
KEGG: ABC transporters
Reactome: Meiotic recombination
Reactome: Histidine, lysine, phenylalanine, tyrosine, proline and tryptophan catabolism
WP: Splicing factor NOVA regulated synaptic proteins
WP: Regulation of Actin Cytoskeleton
KEGG: Serotonergic synapse
KEGG: Glycolysis / Gluconeogenesis
WP: Interleukin-11 Signaling Pathway
WP: IL-6 signaling pathway
WP: ATM Signaling Network in Development and Disease
WP: Tryptophan metabolism
KEGG: Proximal tubule bicarbonate reclamation
Reactome: Interleukin-12 family signaling
WP: Globo Sphingolipid Metabolism
Reactome: Plasma lipoprotein assembly, remodeling, and clearance
WP: AMP-activated Protein Kinase (AMPK) Signaling
WP: Non-small cell lung cancer
KEGG: Retinol metabolism
Reactome: Signaling by EGFR
WP: Thymic Stromal LymphoPoietin (TSLP) Signaling Pathway

WP: Oxidation by Cytochrome P450
Reactome: Incretin synthesis, secretion, and inactivation
Reactome: Activated PKN1 stimulates transcription of AR (androgen receptor) regulated genes KLK2 and KLK3
Reactome: Branched-chain amino acid catabolism
WP: EBV LMP1 signaling
WP: Blood Clotting Cascade
WP: MTHFR deficiency
WP: The effect of progerin on the involved genes in Hutchinson-Gilford Progeria Syndrome
KEGG: Progesterone-mediated oocyte maturation
WP: Pancreatic adenocarcinoma pathway
Reactome: Signaling by FGFR2
WP: Human Thyroid Stimulating Hormone (TSH) signaling pathway
WP: AGE/RAGE pathway
WP: RAC1/PAK1/p38/MMP2 Pathway
WP: Electron Transport Chain (OXPHOS system in mitochondria)
KEGG: Linoleic acid metabolism
KEGG: Vitamin digestion and absorption
Reactome: Mitophagy
Reactome: Other interleukin signaling
Reactome: Signaling by Erythropoietin
WP: Physiological and Pathological Hypertrophy of the Heart
KEGG: Arginine and proline metabolism
WP: Regulation of Microtubule Cytoskeleton
Reactome: Interleukin-7 signaling
Reactome: Insulin processing
Reactome: MyD88 cascade initiated on plasma membrane
KEGG: Ribosome biogenesis in eukaryotes
KEGG: Calcium signaling pathway
Reactome: Eukaryotic Translation Elongation
Reactome: Abnormal conversion of 2-oxoglutarate to 2-hydroxyglutarate
Reactome: NADPH regeneration
Reactome: Vitamin E
WP: Influenza A virus infection
WP: Colchicine Metabolic Pathway
WP: mir34a and TGIF2 in osteoclastogenesis
WP: Gut-Liver Indole Metabolism
WP: Metabolism of Dichloroethylene by CYP450
WP: Metabolism of Tetrahydrocannabinol (THC)
WP: Acrylamide Biotransformation and Exposure Biomarkers
WP: FABP4 in ovarian cancer
WP: Glucose Homeostasis
KEGG: Valine, leucine and isoleucine degradation
Reactome: DNA Double Strand Break Response
Reactome: Selenoamino acid metabolism
KEGG: Collecting duct acid secretion
Reactome: SIRT1 negatively regulates rRNA expression
Reactome: Integrin alphaIIb beta3 signaling
WP: Nanoparticle-mediated activation of receptor signaling
WP: EPO Receptor Signaling

Reactome: Mitochondrial biogenesis
WP: Arrhythmogenic Right Ventricular Cardiomyopathy
Reactome: Complement cascade
KEGG: Phospholipase D signaling pathway
WP: Endoderm Differentiation
KEGG: Oxidative phosphorylation
Reactome: Glycogen metabolism
WP: Sphingolipid Metabolism
WP: Photodynamic therapy-induced unfolded protein response
KEGG: Gastric acid secretion
WP: GPCRs, Other
WP: Preimplantation Embryo
WP: One carbon metabolism and related pathways
KEGG: Thyroid hormone synthesis
Reactome: Glycosaminoglycan metabolism
KEGG: Caffeine metabolism
Reactome: Transport of glycerol from adipocytes to the liver by Aquaporins
Reactome: Pyrophosphate hydrolysis
Reactome: Plasmalogen biosynthesis
Reactome: Intestinal infectious diseases
WP: Lidocaine metabolism
WP: Felbamate Metabolism
WP: Hypoxia-mediated EMT and Stemness
WP: Caffeine and Theobromine metabolism
WP: Ultraconserved region 339 modulation of tumor suppressor microRNAs in cancer
KEGG: Lysine degradation
Reactome: RHO GTPases activate PKNs
WP: TLR4 Signaling and Tolerance
KEGG: Purine metabolism
Reactome: Signaling by NTRK1 (TRKA)
WP: IL-4 Signaling Pathway
Reactome: Interleukin-2 family signaling
Reactome: Protein ubiquitination
WP: PDGFR-beta pathway
WP: Ethanol effects on histone modifications
WP: Inflammatory Response Pathway
Reactome: RAF/MAP kinase cascade
WP: DNA IR-damage and cellular response via ATR
KEGG: D-Glutamine and D-glutamate metabolism
Reactome: Insulin-like Growth Factor-2 mRNA Binding Proteins (IGF2BPs/IMPs/VICKZs) bind RNA
Reactome: RHO GTPases regulate CFTR trafficking
Reactome: Threonine catabolism
Reactome: Metabolism of vitamin K
Reactome: Transcription from mitochondrial promoters
Reactome: Intracellular oxygen transport
Reactome: rRNA modification in the mitochondrion
Reactome: Lactose synthesis
WP: Nicotine Metabolism
WP: Diclofenac Metabolic Pathway
WP: Heroin metabolism

WP: Vitamins A and D - action mechanisms
WP: Model for regulation of MSMP expression in cancer cells and its proangiogenic role in ovarian tumors
WP: Arylamine metabolism
KEGG: Citrate cycle (TCA cycle)
KEGG: Porphyrin and chlorophyll metabolism
KEGG: Autophagy
Reactome: Host Interactions with Influenza Factors
Reactome: PRC2 methylates histones and DNA
Reactome: Cargo concentration in the ER
WP: Extracellular vesicle-mediated signaling in recipient cells
WP: Zinc homeostasis
WP: Tumor suppressor activity of SMARCB1
Reactome: TCF dependent signaling in response to WNT
WP: DNA IR-Double Strand Breaks (DSBs) and cellular response via ATM
Reactome: MAPK targets/ Nuclear events mediated by MAP kinases
Reactome: Thrombin signalling through proteinase activated receptors (PARs)
Reactome: DAG and IP3 signaling
Reactome: DAP12 interactions
WP: Ovarian Infertility Genes
WP: White fat cell differentiation
WP: Monoamine Transport
Reactome: Visual phototransduction
Reactome: GABA receptor activation
KEGG: Relaxin signaling pathway
Reactome: TET1,2,3 and TDG demethylate DNA
Reactome: Wax biosynthesis
Reactome: Lysosomal oligosaccharide catabolism
Reactome: Signaling by MST1
Reactome: rRNA processing
Reactome: NGF processing
WP: Nicotine Activity on Chromaffin Cells
WP: Peroxisomal beta-oxidation of tetracosanoyl-CoA
WP: Secretion of Hydrochloric Acid in Parietal Cells
WP: Cocaine metabolism
WP: Oxytocin signaling
WP: eIF5A regulation in response to inhibition of the nuclear export system
WP: Neurotransmitter Disorders
WP: MicroRNA network associated with chronic lymphocytic leukemia
WP: Polyol Pathway
KEGG: C-type lectin receptor signaling pathway
KEGG: Starch and sucrose metabolism
Reactome: ROS, RNS production in phagocytes
Reactome: Striated Muscle Contraction
KEGG: SNARE interactions in vesicular transport
Reactome: MyD88 dependent cascade initiated on endosome
Reactome: Sialic acid metabolism
WP: GPCRs, Class A Rhodopsin-like
KEGG: Phenylalanine, tyrosine and tryptophan biosynthesis
KEGG: Phosphonate and phosphinate metabolism
KEGG: Neomycin, kanamycin and gentamicin biosynthesis

Reactome: Transmission across Electrical Synapses
Reactome: FasL/ CD95L signaling
Reactome: Protein repair
Reactome: tRNA processing in the mitochondrion
Reactome: MTF1 activates gene expression
Reactome: Formation of xylulose-5-phosphate
Reactome: Reelin signalling pathway
WP: Sulindac Metabolic Pathway
WP: exRNA mechanism of action and biogenesis
WP: TCA Cycle Nutrient Utilization and Invasiveness of Ovarian Cancer
WP: Synthesis and Degradation of Ketone Bodies
WP: Dual hijack model of Vif in HIV infection
WP: miR-517 relationship with ARCN1 and USP1
WP: Vitamin B6-dependent and responsive disorders
WP: Lamin A-processing pathway
WP: Arachidonate Epoxygenase / Epoxide Hydrolase
WP: Catalytic cycle of mammalian Flavin-containing MonoOxygenases (FMOs)
KEGG: Axon guidance
Reactome: Signaling by FGFR1
KEGG: Peroxisome
Reactome: Metabolism of polyamines
WP: Ras Signaling
Reactome: MyD88:MAL(TIRAP) cascade initiated on plasma membrane
Reactome: ERCC6 (CSB) and EHMT2 (G9a) positively regulate rRNA expression
WP: Fatty Acid Beta Oxidation
WP: GABA receptor Signaling
WP: TYROBP Causal Network
WP: Androgen receptor signaling pathway
KEGG: MAPK signaling pathway
KEGG: Vitamin B6 metabolism
Reactome: Synthesis of wybutosine at G37 of tRNA(Phe)
Reactome: Fructose metabolism
Reactome: Lipid particle organization
Reactome: Choline catabolism
Reactome: Galactose catabolism
WP: Thyroxine (Thyroid Hormone) Production
WP: Metastatic brain tumor
WP: miRNA Biogenesis
WP: Gastric acid production
WP: DDX1 as a regulatory component of the Drosha microprocessor
WP: let-7 inhibition of ES cell reprogramming
WP: mir-124 predicted interactions with cell cycle and differentiation
WP: Robo4 and VEGF Signaling Pathways Crosstalk
WP: Somatroph axis (GH) and its relationship to dietary restriction and aging
WP: Disorders of the Krebs cycle
WP: Non-homologous end joining
Reactome: Mitochondrial Fatty Acid Beta-Oxidation
WP: Nucleotide-binding Oligomerization Domain (NOD) pathway
Reactome: Potassium Channels
KEGG: Human cytomegalovirus infection

WP: Angiopoietin Like Protein 8 Regulatory Pathway
KEGG: Glutamatergic synapse
Reactome: Mitotic Prophase
Reactome: Semaphorin interactions
WP: Pathways Affected in Adenoid Cystic Carcinoma
WP: Parkinsons Disease Pathway
WP: miRNAs involvement in the immune response in sepsis
KEGG: Apelin signaling pathway
WP: Integrated Breast Cancer Pathway
Reactome: Interleukin-1 processing
Reactome: Uptake and function of anthrax toxins
Reactome: Interleukin-17 signaling
Reactome: TYSND1 cleaves peroxisomal proteins
WP: Pentose Phosphate Pathway
WP: Effects of Nitric Oxide
WP: Glial Cell Differentiation
WP: EV release from cardiac cells and their functional effects
WP: FTO Obesity Variant Mechanism
WP: MicroRNA for Targeting Cancer Growth and Vascularization in Glioblastoma
WP: Tgif disruption of Shh signaling
WP: Hfe effect on hepcidin production
WP: Acetylcholine Synthesis
WP: Benzo(a)pyrene metabolism
KEGG: Toll-like receptor signaling pathway
KEGG: Oocyte meiosis
Reactome: G alpha (i) signalling events
KEGG: Aldosterone-regulated sodium reabsorption
KEGG: Longevity regulating pathway
Reactome: EPH-Ephrin signaling
KEGG: Necroptosis
WP: Endometrial cancer
KEGG: Riboflavin metabolism
KEGG: Sulfur metabolism
KEGG: Sulfur relay system
Reactome: DNA Damage Reversal
Reactome: GP1b-IX-V activation signalling
Reactome: mRNA Editing
Reactome: Ubiquinol biosynthesis
Reactome: Base-Excision Repair, AP Site Formation
Reactome: tRNA modification in the mitochondrion
WP: Phase I biotransformations, non P450
WP: TFs Regulate miRNAs related to cardiac hypertrophy
WP: Folate-Alcohol and Cancer Pathway Hypotheses
WP: NAD Biosynthesis II (from tryptophan)
WP: NLR Proteins
WP: Insulin signalling in human adipocytes (normal condition)
WP: Insulin signalling in human adipocytes (diabetic condition)
WP: ApoE and miR-146 in inflammation and atherosclerosis
WP: Metabolism of Spingolipids in ER and Golgi apparatus
Reactome: Signaling by SCF-KIT

Reactome: MAP kinase activation
Reactome: Activation of NMDA receptors and postsynaptic events
WP: Toll-like Receptor Signaling Pathway
Reactome: Macroautophagy
KEGG: Pancreatic secretion
Reactome: Bile acid and bile salt metabolism
Reactome: Nonhomologous End-Joining (NHEJ)
WP: EGF/EGFR Signaling Pathway
Reactome: Neurotransmitter clearance
Reactome: Signal regulatory protein family interactions
Reactome: Abacavir transport and metabolism
Reactome: Neurotransmitter receptors and postsynaptic signal transmission
Reactome: RHO GTPases Activate Rhotekin and Rhophilins
Reactome: HDR through MMEJ (alt-NHEJ)
Reactome: Mitochondrial calcium ion transport
Reactome: Butyrophilin (BTN) family interactions
Reactome: Interleukin-9 signaling
Reactome: Serine biosynthesis
WP: Codeine and Morphine Metabolism
WP: Cytosine methylation
WP: ATR Signaling
WP: Macrophage markers
WP: Degradation pathway of sphingolipids, including diseases
WP: Thiamine metabolic pathways
WP: Mismatch repair
WP: Heme Biosynthesis
KEGG: Aldosterone synthesis and secretion
Reactome: Eukaryotic Translation Termination
KEGG: NOD-like receptor signaling pathway
KEGG: Ether lipid metabolism
KEGG: Carbohydrate digestion and absorption
Reactome: SUMOylation of transcription cofactors
Reactome: Deubiquitination
Reactome: Platelet Adhesion to exposed collagen
Reactome: Neurotoxicity of clostridium toxins
Reactome: Mitochondrial iron-sulfur cluster biogenesis
Reactome: SUMOylation
Reactome: Caspase activation via Dependence Receptors in the absence of ligand
Reactome: RHO GTPases activate IQGAPs
Reactome: Ketone body metabolism
Reactome: Tetrahydrobiopterin (BH4) synthesis, recycling, salvage and regulation
WP: SRF and miRs in Smooth Muscle Differentiation and Proliferation
WP: Irinotecan Pathway
WP: Trans-sulfuration pathway
WP: Interleukin-1 Induced Activation of NF-kappa-B
WP: Leptin and adiponectin
KEGG: Cardiac muscle contraction
WP: Peptide GPCRs
WP: Glycogen Metabolism
WP: Brain-Derived Neurotrophic Factor (BDNF) signaling pathway

KEGG: Taurine and hypotaurine metabolism
KEGG: Non-homologous end-joining
Reactome: RHO GTPases activate KTN1
Reactome: LGI-ADAM interactions
Reactome: Vitamin D (calciferol) metabolism
Reactome: SUMOylation of immune response proteins
WP: Serotonin Transporter Activity
WP: Fatty Acid Omega Oxidation
WP: RalA downstream regulated genes
WP: NAD metabolism, sirtuins and aging
WP: PTF1A related regulatory pathway
WP: Nucleotide GPCRs
KEGG: Fatty acid degradation
WP: Heart Development
Reactome: Host Interactions of HIV factors
KEGG: Prolactin signaling pathway
Reactome: Immunoregulatory interactions between a Lymphoid and a non-Lymphoid cell
KEGG: Cysteine and methionine metabolism
Reactome: Vasopressin regulates renal water homeostasis via Aquaporins
Reactome: Presynaptic depolarization and calcium channel opening
Reactome: Rap1 signalling
Reactome: Prolactin receptor signaling
Reactome: RHO GTPases Activate NADPH Oxidases
Reactome: Import of palmitoyl-CoA into the mitochondrial matrix
Reactome: Protein methylation
Reactome: Processing of SMDT1
WP: Homologous recombination
WP: Cell Differentiation - Index
WP: Nanoparticle triggered regulated necrosis
WP: Cell-type Dependent Selectivity of CCK2R Signaling
WP: MAPK and NFkB Signalling Pathways Inhibited by Yersinia YopJ
WP: Transcriptional cascade regulating adipogenesis
Reactome: Metabolism of water-soluble vitamins and cofactors
KEGG: Sphingolipid metabolism
KEGG: Biosynthesis of amino acids
KEGG: Thiamine metabolism
Reactome: Cytosolic iron-sulfur cluster assembly
Reactome: Receptor-type tyrosine-protein phosphatases
Reactome: Interleukin-15 signaling
WP: Estrogen Receptor Pathway
WP: MFAP5-mediated ovarian cancer cell motility and invasiveness
WP: Kennedy pathway from Sphingolipids
WP: Disorders of Folate Metabolism and Transport
WP: Vitamin B12 Disorders
WP: Hedgehog Signaling Pathway
WP: Tamoxifen metabolism
WP: NO/cGMP/PKG mediated Neuroprotection
Reactome: Unfolded Protein Response (UPR)
Reactome: ATF6 (ATF6-alpha) activates chaperone genes
Reactome: Regulation of TP53 Activity through Association with Co-factors

Reactome: Pentose phosphate pathway
Reactome: SUMOylation of DNA methylation proteins
WP: ERK Pathway in Huntington's Disease
WP: GPR40 Pathway
WP: Tryptophan catabolism leading to NAD+ production
WP: Allograft Rejection
KEGG: N-Glycan biosynthesis
Reactome: Amyloid fiber formation
KEGG: Salivary secretion
KEGG: Ascorbate and aldarate metabolism
KEGG: Glycosphingolipid biosynthesis
KEGG: Glycosphingolipid biosynthesis
Reactome: Mismatch Repair
Reactome: Metabolism of folate and pterines
Reactome: RUNX1 and FOXP3 control the development of regulatory T lymphocytes (Tregs)
Reactome: Signaling by NTRK3 (TRKC)
WP: GPCRs, Class C Metabotropic glutamate, pheromone
WP: ACE Inhibitor Pathway
Reactome: B-WICH complex positively regulates rRNA expression
Reactome: E3 ubiquitin ligases ubiquitinate target proteins
Reactome: ABC-family proteins mediated transport
Reactome: Apoptotic execution phase
Reactome: Meiotic synapsis
Reactome: tRNA processing in the nucleus
WP: Translation Factors
Reactome: Nephrin family interactions
Reactome: Signaling by NODAL
Reactome: YAP1- and WWTR1 (TAZ)-stimulated gene expression
WP: Drug Induction of Bile Acid Pathway
WP: Deregulation of Rab and Rab Effector Genes in Bladder Cancer
WP: Interactome of polycomb repressive complex 2 (PRC2)
WP: NAD+ metabolism
WP: Regulation of toll-like receptor signaling pathway
WP: Leptin signaling pathway
Reactome: Transcriptional activity of SMAD2/SMAD3:SMAD4 heterotrimer
Reactome: Nucleotide-binding domain, leucine rich repeat containing receptor (NLR) signaling pathways
KEGG: Primary bile acid biosynthesis
KEGG: Mannose type O-glycan biosynthesis
KEGG: 2-Oxocarboxylic acid metabolism
Reactome: Regulation of TP53 Activity through Methylation
Reactome: Synaptic adhesion-like molecules
WP: IL-9 Signaling Pathway
WP: Mitochondrial LC-Fatty Acid Beta-Oxidation
WP: Simplified Depiction of MYD88 Distinct Input-Output Pathway
WP: Mitochondrial Gene Expression
WP: Alzheimers Disease
Reactome: The citric acid (TCA) cycle and respiratory electron transport
WP: Synaptic Vesicle Pathway
WP: Phosphodiesterases in neuronal function
KEGG: Ubiquitin mediated proteolysis

KEGG: Other glycan degradation
KEGG: One carbon pool by folate
KEGG: Pantothenate and CoA biosynthesis
Reactome: GABA synthesis, release, reuptake and degradation
Reactome: Signaling by Hippo
Reactome: SALM protein interactions at the synapse
Reactome: Digestion
WP: Ganglio Sphingolipid Metabolism
WP: Cell Differentiation - Index expanded
WP: Hematopoietic Stem Cell Gene Regulation by GABP alpha/beta Complex
WP: Inhibition of exosome biogenesis and secretion by Manumycin A in CRPC cells
WP: TCA Cycle (aka Krebs or citric acid cycle)
Reactome: Translocation of SLC2A4 (GLUT4) to the plasma membrane
WP: Wnt Signaling Pathway
Reactome: Sphingolipid metabolism
Reactome: Cargo recognition for clathrin-mediated endocytosis
KEGG: Glycosaminoglycan degradation
Reactome: Listeria monocytogenes entry into host cells
Reactome: Class C/3 (Metabotropic glutamate/pheromone receptors)
WP: Small Ligand GPCRs
WP: Apoptosis Modulation by HSP70
WP: Serotonin Receptor 4/6/7 and NR3C Signaling
WP: Oxidative phosphorylation
Reactome: Integration of energy metabolism
KEGG: Glycosaminoglycan biosynthesis
Reactome: POU5F1 (OCT4), SOX2, NANOG activate genes related to proliferation
Reactome: rRNA modification in the nucleus and cytosol
WP: Nicotine Activity on Dopaminergic Neurons
WP: Nanomaterial induced apoptosis
WP: Glycerophospholipid Biosynthetic Pathway
WP: Triacylglyceride Synthesis
WP: Hereditary leiomyomatosis and renal cell carcinoma pathway
Reactome: Telomere Maintenance
Reactome: Respiratory electron transport, ATP synthesis by chemiosmotic coupling, and heat production by uncoupling proteins.
KEGG: Insulin secretion
Reactome: RNA Polymerase I Transcription
Reactome: Signaling by MET
KEGG: Pentose and glucuronate interconversions
WP: Type II diabetes mellitus
WP: NAD+ biosynthetic pathways
Reactome: SRP-dependent cotranslational protein targeting to membrane
KEGG: Carbon metabolism
Reactome: Signaling by ROBO receptors
KEGG: Protein export
KEGG: Mismatch repair
Reactome: Peroxisomal lipid metabolism
WP: miRNA regulation of p53 pathway in prostate cancer
WP: Estrogen signaling pathway
Reactome: MHC class II antigen presentation

KEGG: Leukocyte transendothelial migration
KEGG: VEGF signaling pathway
Reactome: Neurexins and neuroligins
WP: Proteasome Degradation
KEGG: Glycosylphosphatidylinositol (GPI)-anchor biosynthesis
KEGG: Folate biosynthesis
Reactome: Signaling by NOTCH4
Reactome: Gene Silencing by RNA
Reactome: Interleukin-1 family signaling
KEGG: Long-term depression
KEGG: NF-kappa B signaling pathway
KEGG: Fc gamma R-mediated phagocytosis
WP: Corticotropin-releasing hormone signaling pathway
WP: Cytoplasmic Ribosomal Proteins
Reactome: Nonsense-Mediated Decay (NMD)
KEGG: Retrograde endocannabinoid signaling
KEGG: Glycosaminoglycan biosynthesis
Reactome: Miscellaneous transport and binding events
WP: Kit receptor signaling pathway
KEGG: Thyroid hormone signaling pathway
KEGG: GABAergic synapse
Reactome: Fertilization
Reactome: Glutamate binding, activation of AMPA receptors and synaptic plasticity
WP: Follicle Stimulating Hormone (FSH) signaling pathway
WP: Signal Transduction of S1P Receptor
WP: Pregnane X Receptor pathway
Reactome: RAB geranylgeranylation
WP: Notch Signaling Pathway
KEGG: Vascular smooth muscle contraction
KEGG: Phototransduction
Reactome: Toll-like Receptor Cascades
Reactome: Sulfur amino acid metabolism
WP: Constitutive Androstane Receptor Pathway
Reactome: Eukaryotic Translation Initiation
Reactome: RUNX1 regulates genes involved in megakaryocyte differentiation and platelet function
Reactome: MAPK6/MAPK4 signaling
Reactome: Cellular response to heat stress
KEGG: Endocytosis
KEGG: Pentose phosphate pathway
KEGG: Hippo signaling pathway
Reactome: Effects of PIP2 hydrolysis
Reactome: Processing of Capped Intronless Pre-mRNA
Reactome: mTOR signalling
WP: Cannabinoid receptor signaling
WP: TNF alpha Signaling Pathway
Reactome: L1CAM interactions
KEGG: Mitophagy
KEGG: Synaptic vesicle cycle
KEGG: RNA polymerase
Reactome: Gap junction trafficking and regulation

Reactome: Myogenesis
Reactome: Surfactant metabolism
Reactome: Regulation of TP53 Activity through Acetylation
Reactome: Cristae formation
Reactome: G-protein beta:gamma signalling
Reactome: SUMOylation of SUMOylation proteins
WP: miRs in Muscle Cell Differentiation
WP: One Carbon Metabolism
WP: T-Cell Receptor and Co-stimulatory Signaling
KEGG: Th17 cell differentiation
KEGG: Oxytocin signaling pathway
Reactome: NoRC negatively regulates rRNA expression
Reactome: mRNA Capping
Reactome: Energy dependent regulation of mTOR by LKB1-AMPK
Reactome: ATF4 activates genes
Reactome: Endosomal Sorting Complex Required For Transport (ESCRT)
WP: IL17 signaling pathway
WP: Trans-sulfuration and one carbon metabolism
WP: Initiation of transcription and translation elongation at the HIV-1 LTR
WP: Canonical and Non-canonical Notch signaling
WP: MAPK Cascade
Reactome: Hedgehog ligand biogenesis
KEGG: Galactose metabolism
Reactome: Glyoxylate metabolism and glycine degradation
WP: Toll-like Receptor Signaling
WP: Prion disease pathway
Reactome: COPII-mediated vesicle transport
KEGG: Fructose and mannose metabolism
Reactome: GPVI-mediated activation cascade
Reactome: Signal amplification
Reactome: tRNA modification in the nucleus and cytosol
Reactome: SUMOylation of ubiquitinylation proteins
KEGG: Autophagy
WP: Nuclear Receptors in Lipid Metabolism and Toxicity
Reactome: SUMOylation of DNA damage response and repair proteins
Reactome: Signaling by VEGF
KEGG: Base excision repair
Reactome: Transcriptional regulation of pluripotent stem cells
WP: Signaling of Hepatocyte Growth Factor Receptor
WP: BDNF-TrkB Signaling
Reactome: RHO GTPases Activate WASPs and WAVEs
Reactome: Fanconi Anemia Pathway
WP: p38 MAPK Signaling Pathway
KEGG: Adipocytokine signaling pathway
Reactome: Signaling by NOTCH2
Reactome: Toll Like Receptor 3 (TLR3) Cascade
KEGG: Ribosome
Reactome: trans-Golgi Network Vesicle Budding
KEGG: Homologous recombination
Reactome: MyD88-independent TLR4 cascade

Reactome: Resolution of Abasic Sites (AP sites)
Reactome: NCAM signaling for neurite out-growth
Reactome: HSP90 chaperone cycle for steroid hormone receptors (SHR)
WP: Histone Modifications
WP: Amyotrophic lateral sclerosis (ALS)
WP: G13 Signaling Pathway
Reactome: Clathrin-mediated endocytosis
Reactome: SUMOylation of RNA binding proteins
WP: Common Pathways Underlying Drug Addiction
WP: Chemokine signaling pathway
KEGG: Glycine, serine and threonine metabolism
Reactome: TNF signaling
WP: Microglia Pathogen Phagocytosis Pathway
Reactome: Cardiac conduction
KEGG: cGMP-PKG signaling pathway
KEGG: Insulin signaling pathway
Reactome: Signaling by ERBB2
WP: IL-5 Signaling Pathway
WP: Eukaryotic Transcription Initiation
Reactome: Influenza Life Cycle
WP: Prolactin Signaling Pathway
KEGG: Basal transcription factors
Reactome: tRNA Aminoacylation
Reactome: Signaling by ERBB4
Reactome: RNA Polymerase III Transcription
WP: IL-2 Signaling Pathway
KEGG: Th1 and Th2 cell differentiation
Reactome: Factors involved in megakaryocyte development and platelet production
Reactome: Platelet homeostasis
KEGG: Aminoacyl-tRNA biosynthesis
Reactome: Interleukin-3, Interleukin-5 and GM-CSF signaling
Reactome: TBC/RABGAPs
WP: Fas Ligand (FasL) pathway and Stress induction of Heat Shock Proteins (HSP) regulation
KEGG: Nucleotide excision repair
KEGG: Vasopressin-regulated water reabsorption
Reactome: Opioid Signalling
Reactome: Fcgamma receptor (FCGR) dependent phagocytosis
KEGG: Proteasome
KEGG: Endocrine and other factor-regulated calcium reabsorption
Reactome: G alpha (z) signalling events
KEGG: mRNA surveillance pathway
Reactome: Glucose metabolism
KEGG: Cholinergic synapse
Reactome: Beta-catenin independent WNT signaling
KEGG: Fanconi anemia pathway
Reactome: Metabolism of non-coding RNA
Reactome: Inositol phosphate metabolism
WP: Energy Metabolism
Reactome: Regulation of mRNA stability by proteins that bind AU-rich elements
Reactome: TP53 Regulates Metabolic Genes

KEGG: Amino sugar and nucleotide sugar metabolism
Reactome: SUMOylation of chromatin organization proteins
WP: IL-3 Signaling Pathway
KEGG: Platelet activation
KEGG: RNA transport
WP: Structural Pathway of Interleukin 1 (IL-1)
KEGG: Glycerophospholipid metabolism
Reactome: Hedgehog 'on' state
KEGG: Glycerolipid metabolism
Reactome: Assembly of the primary cilium
Reactome: Cilium Assembly
WP: Calcium Regulation in the Cardiac Cell
Reactome: Glycerophospholipid biosynthesis
Reactome: Regulation of Apoptosis
Reactome: Deadenylation-dependent mRNA decay
Reactome: Asparagine N-linked glycosylation
Reactome: Costimulation by the CD28 family
Reactome: Regulation of TP53 Activity through Phosphorylation
WP: T-Cell antigen Receptor (TCR) Signaling Pathway
KEGG: Thermogenesis
Reactome: Activation of anterior HOX genes in hindbrain development during early embryogenesis
WP: G Protein Signaling Pathways
WP: MET in type 1 papillary renal cell carcinoma
Reactome: Major pathway of rRNA processing in the nucleolus and cytosol
KEGG: Natural killer cell mediated cytotoxicity
Reactome: Signaling by NOTCH3
Reactome: Keratinization
WP: IL-1 signaling pathway
WP: RANKL/RANK (Receptor activator of NFKB (ligand)) Signaling Pathway
KEGG: Human immunodeficiency virus 1 infection
KEGG: Circadian entrainment
Reactome: p75 NTR receptor-mediated signalling
Reactome: Cytosolic sensors of pathogen-associated DNA
Reactome: TNFR2 non-canonical NF-kB pathway
Reactome: NIK-->noncanonical NF-kB signaling
KEGG: Glucagon signaling pathway
Reactome: Regulation of beta-cell development
Reactome: Peroxisomal protein import
Reactome: Signaling by Rho GTPases
WP: MAPK Signaling Pathway
Reactome: Class I MHC mediated antigen processing & presentation
WP: T-Cell antigen Receptor (TCR) pathway during Staphylococcus aureus infection
KEGG: cAMP signaling pathway
Reactome: Mitochondrial protein import
Reactome: Transcriptional regulation by RUNX1
KEGG: Long-term potentiation
KEGG: Adherens junction
KEGG: Fc epsilon RI signaling pathway
Reactome: HDR through Homologous Recombination (HRR) or Single Strand Annealing (SSA)
KEGG: Taste transduction

Reactome: RNA polymerase II transcribes snRNA genes
KEGG: Parathyroid hormone synthesis, secretion and action
Reactome: Ion channel transport
KEGG: Adrenergic signaling in cardiomyocytes
KEGG: B cell receptor signaling pathway
Reactome: TP53 Regulates Transcription of DNA Repair Genes
KEGG: Chemokine signaling pathway
KEGG: RNA degradation
KEGG: Inositol phosphate metabolism
Reactome: Regulation of RUNX2 expression and activity
Reactome: Signaling by NOTCH1
Reactome: Regulation of Hypoxia-inducible Factor (HIF) by oxygen
Reactome: G alpha (12/13) signalling events
Reactome: PI Metabolism
KEGG: Osteoclast differentiation
KEGG: Neurotrophin signaling pathway
KEGG: Sphingolipid signaling pathway
Reactome: COPI-mediated anterograde transport
KEGG: Lysosome
WP: mRNA Processing
Reactome: Degradation of beta-catenin by the destruction complex
Reactome: RAB GEFs exchange GTP for GDP on RABs
KEGG: Protein processing in endoplasmic reticulum
KEGG: Spliceosome
WP: Insulin Signaling
KEGG: GnRH signaling pathway
Reactome: C-type lectin receptors (CLRs)
Reactome: Mitochondrial translation
KEGG: Estrogen signaling pathway
Reactome: Transcriptional regulation of white adipocyte differentiation
Reactome: Neddylation
KEGG: Phosphatidylinositol signaling system
Reactome: XBP1(S) activates chaperone genes
Reactome: HIV Life Cycle
Reactome: Intra-Golgi and retrograde Golgi-to-ER traffic
WP: B Cell Receptor Signaling Pathway
Reactome: Hedgehog 'off' state
KEGG: T cell receptor signaling pathway
Reactome: TCR signaling
Reactome: Signaling by the B Cell Receptor (BCR)
Reactome: Nucleotide Excision Repair
Reactome: Generic Transcription Pathway
KEGG: Dopaminergic synapse
Reactome: Fc epsilon receptor (FCERI) signaling
Reactome: PTEN Regulation
Reactome: RNA Polymerase II Transcription
Reactome: Chromatin organization
KEGG: Olfactory transduction
Reactome: Processing of Capped Intron-Containing Pre-mRNA
Reactome: G alpha (s) signalling events

WP: Aripiprazole Metabolic Pathway
WP: Human metabolism overview
WP: Biochemical Pathways Part I
WP: Biosynthesis and regeneration of tetrahydrobiopterin (BH4) and catabolism of phenylalanine, including diseases
WP: GHB metabolic pathway
WP: Mevalonate arm of cholesterol biosynthesis pathway
WP: Phosphatidylcholine catabolism
WP: Amino acid conjugation

**Positive: the number of genes on the pathway that pass the statistical criteria (absolute logFC > 0.58 and p-value < 0.05)*
**Measured: the number of genes on the pathway that were measured in the dataset*
WP: WikiPathways
The red line indicates the end of the significantly changed pathways for this analysis

Z Score	Permuted p-value	Positive*	Measured*
16,48	< 0,0001	14	14
15,13	< 0,0001	16	21
14,86	< 0,0001	27	57
11,65	< 0,0001	7	7
10,14	< 0,0001	21	66
8,86	< 0,0001	9	18
7,34	< 0,0001	13	46
7,04	< 0,0001	8	21
6,88	< 0,0001	14	57
6,45	< 0,0001	7	19
6,23	< 0,0001	2	2
5,92	< 0,0001	14	69
5,72	< 0,0001	24	166
5,6	0,001	5	13
5,39	< 0,0001	18	114
5,15	< 0,0001	16	100
5,06	< 0,0001	7	27
4,95	< 0,0001	10	50
4,88	< 0,0001	5	16
4,56	< 0,0001	6	24
4,56	0,003	6	24
4,54	< 0,0001	13	83
4,49	< 0,0001	5	18
4,4	< 0,0001	1	1
4,4	< 0,0001	1	1
4,4	< 0,0001	1	1
4,4	< 0,0001	12	76
4,21	< 0,0001	15	111
4,17	0,006	2	4
4,17	0,001	2	4
4,17	0,004	6	27
4,16	< 0,0001	8	43
4,13	< 0,0001	14	102
3,95	0,001	3	9
3,9	< 0,0001	4	15
3,87	0,002	5	22
3,77	0,001	7	39
3,77	0,001	11	78
3,74	0,003	5	23
3,74	0,002	5	23
3,7	< 0,0001	8	49
3,63	0,005	2	5
3,62	0,002	12	92
3,62	0,001	11	81
3,57	0,003	9	61

3,53	0,001	6	33
3,53	0,006	6	33
3,51	< 0,0001	9	62
3,48	0,001	17	156
3,42	< 0,0001	20	197
3,4	0,004	4	18
3,35	< 0,0001	6	35
3,35	0,002	11	87
3,34	0,007	9	65
3,31	0,002	8	55
3,29	0,002	10	77
3,28	0,005	26	287
3,28	0,005	16	150
3,28	0,01	5	27
3,27	0,002	6	36
3,22	0,006	2	6
3,22	0,004	11	90
3,19	0,007	6	37
3,18	0,005	9	68
3,17	0,003	17	167
3,03	0,01	3	13
3,03	0,019	3	13
2,98	0,009	7	50
2,98	0,011	7	50
2,97	0,004	8	61
2,95	0,013	1	2
2,95	0,016	1	2
2,95	0,01	1	2
2,95	0,008	13	122
2,92	0,013	7	51
2,9	0,02	2	7
2,89	0,009	6	41
2,88	0,018	4	22
2,86	0,019	3	14
2,86	0,025	3	14
2,86	0,013	7	52
2,82	0,008	12	113
2,77	0,01	4	23
2,77	0,02	4	23
2,75	0,019	6	43
2,71	0,011	3	15
2,67	0,013	8	67
2,65	0,012	12	118
2,62	0,01	6	45
2,59	0,016	9	81
2,59	0,01	9	81
2,57	0,007	4	25
2,57	0,014	4	25
2,55	0,008	9	82
2,53	0,016	8	70

2,49	0,017	21	254
2,48	0,014	8	71
2,45	0,022	11	111
2,43	0,022	3	17
2,4	0,048	2	9
2,38	0,014	6	49
2,38	0,013	6	49
2,38	0,013	13	141
2,38	0,01	7	61
2,35	0,018	12	128
2,31	0,028	3	18
2,31	0,033	3	18
2,31	0,02	14	158
2,28	0,041	1	3
2,28	0,028	1	3
2,23	0,022	8	77
2,22	0,028	5	40
2,21	0,048	2	10
2,21	0,054	2	10
2,2	0,02	3	19
2,2	0,055	3	19
2,16	0,031	5	41
2,14	0,031	4	30
2,09	0,031	3	20
2,09	0,043	3	20
2,09	0,048	3	20
2,06	0,042	4	31
2,06	0,039	4	31
2,06	0,037	4	31
2,06	0,033	4	31
2,04	0,036	2	11
2,04	0,043	8	82
2,04	0,037	8	82
2,02	0,046	7	69
2,01	0,024	6	56
1,99	0,037	3	21
1,99	0,035	3	21
1,99	0,039	4	32
1,99	0,029	13	156
1,98	0,043	5	44
1,96	0,052	8	84
1,94	0,045	11	128
1,94	0,037	11	128
1,92	0,041	4	33
1,92	0,045	10	114
1,9	0,057	3	22
1,89	0,053	2	12
1,89	0,068	2	12
1,89	0,032	2	12
1,86	0,058	1	4

1,86	0,067	1	4
1,86	0,082	1	4
1,86	0,071	1	4
1,86	0,045	8	87
1,85	0,048	4	34
1,82	0,057	5	47
1,82	0,049	5	47
1,79	0,067	4	35
1,78	0,073	12	149
1,78	0,047	7	75
1,76	0,067	23	329
1,75	0,074	2	13
1,75	0,055	2	13
1,75	0,061	2	13
1,74	0,059	10	120
1,74	0,069	6	62
1,72	0,067	4	36
1,71	0,08	9	106
1,71	0,092	18	249
1,7	0,069	6	63
1,7	0,059	6	63
1,69	0,08	22	317
1,67	0,059	5	50
1,66	0,084	4	37
1,64	0,086	3	25
1,63	0,108	7	79
1,62	0,06	2	14
1,62	0,114	8	94
1,61	0,104	28	426
1,6	0,079	4	38
1,57	0,074	3	26
1,56	0,091	1	5
1,56	0,071	1	5
1,55	0,083	4	39
1,55	0,079	4	39
1,55	0,083	4	39
1,55	0,089	4	39
1,54	0,105	14	192
1,53	0,103	8	97
1,53	0,081	8	97
1,51	0,086	2	15
1,51	0,124	2	15
1,51	0,12	2	15
1,5	0,131	11	145
1,49	0,085	3	27
1,49	0,081	4	40
1,49	0,092	7	83
1,49	0,105	7	83
1,45	0,155	10	131
1,44	0,081	4	41

1,42	0,156	10	132
1,42	0,176	3	28
1,42	0,099	3	28
1,42	0,101	3	28
1,41	0,117	2	16
1,41	0,088	2	16
1,39	0,096	5	56
1,36	0,124	3	29
1,35	0,202	6	72
1,33	0,084	1	6
1,33	0,091	1	6
1,33	0,129	1	6
1,33	0,157	1	6
1,33	0,102	1	6
1,33	0,183	7	88
1,33	0,194	12	169
1,31	0,2	5	58
1,31	0,101	2	17
1,31	0,133	2	17
1,31	0,141	2	17
1,29	0,16	7	89
1,29	0,2	3	30
1,29	0,111	3	30
1,29	0,123	3	30
1,29	0,212	4	44
1,29	0,116	4	44
1,25	0,177	10	139
1,24	0,109	4	45
1,23	0,122	3	31
1,23	0,208	5	60
1,23	0,216	19	295
1,2	0,262	13	192
1,2	0,233	13	192
1,18	0,229	66	1172
1,18	0,226	8	109
1,17	0,312	6	77
1,15	0,118	1	7
1,15	0,141	1	7
1,15	0,125	1	7
1,15	0,173	1	7
1,15	0,138	1	7
1,15	0,158	1	7
1,14	0,209	4	47
1,14	0,229	6	78
1,13	0,132	2	19
1,13	0,145	2	19
1,13	0,125	2	19
1,13	0,272	2	19
1,12	0,25	18	284

1,11	0,263	5	63
1,11	0,257	7	95
1,11	0,25	6	79
1,1	0,279	8	112
1,08	0,36	5	64
1,07	0,294	8	113
1,06	0,294	3	34
1,05	0,305	7	97
1,05	0,307	2	20
1,05	0,27	2	20
1,04	0,267	6	81
1	0,391	5	66
1	0,151	3	35
1	0,323	3	35
1	0,403	3	35
1	0,346	3	35
0,99	0,117	1	8
0,99	0,191	1	8
0,99	0,198	1	8
0,99	0,311	14	221
0,98	0,309	6	83
0,98	0,309	6	83
0,98	0,163	2	21
0,98	0,204	2	21
0,97	0,33	5	67
0,97	0,312	5	67
0,97	0,425	5	67
0,95	0,432	3	36
0,95	0,345	9	135
0,93	0,348	4	52
0,92	0,344	6	85
0,91	0,451	2	22
0,91	0,412	2	22
0,91	0,468	2	22
0,91	0,428	2	22
0,91	0,218	2	22
0,9	0,388	9	137
0,9	0,357	3	37
0,9	0,396	5	69
0,89	0,467	4	53
0,87	0,397	10	156
0,86	0,538	1	9
0,85	0,417	3	38
0,84	0,236	2	23
0,84	0,264	2	23
0,84	0,247	2	23
0,83	0,375	5	71
0,81	0,425	10	159
0,81	0,426	3	39
0,78	0,476	2	24

0,78	0,414	2	24
0,78	0,425	2	24
0,78	0,321	2	24
0,77	0,416	6	90
0,76	0,384	3	40
0,76	0,496	7	108
0,75	0,491	9	144
0,75	0,383	1	10
0,75	0,559	1	10
0,75	0,338	1	10
0,75	0,579	1	10
0,75	0,435	1	10
0,74	0,461	5	74
0,73	0,484	7	109
0,73	0,512	8	127
0,72	0,518	2	25
0,72	0,603	2	25
0,72	0,495	2	25
0,72	0,568	2	25
0,71	0,487	3	41
0,71	0,628	3	41
0,71	0,555	3	41
0,71	0,462	9	146
0,69	0,47	6	93
0,68	0,445	5	76
0,66	0,689	2	26
0,66	0,5	2	26
0,66	0,689	2	26
0,64	0,539	1	11
0,64	0,594	1	11
0,64	0,22	1	11
0,63	0,586	4	60
0,63	0,548	4	60
0,63	0,629	3	43
0,63	0,562	3	43
0,61	0,55	6	96
0,6	0,634	2	27
0,59	0,548	3	44
0,59	0,629	3	44
0,58	0,571	9	152
0,56	0,505	4	62
0,55	0,791	1	12
0,55	0,711	1	12
0,55	0,891	1	12
0,55	0,606	1	12
0,55	0,388	1	12
0,55	0,554	1	12
0,55	0,634	1	12
0,55	0,561	1	12
0,55	0,655	3	45

0,54	0,612	7	117
0,53	0,597	4	63
0,52	0,598	7	118
0,51	0,605	3	46
0,51	0,638	3	46
0,5	0,592	5	82
0,5	0,609	4	64
0,5	0,859	2	29
0,5	0,633	2	29
0,5	0,884	2	29
0,47	0,661	5	83
0,47	0,691	5	83
0,47	0,651	3	47
0,47	0,69	3	47
0,47	0,59	3	47
0,46	0,612	1	13
0,46	0,923	1	13
0,46	0,705	1	13
0,46	0,61	1	13
0,46	0,645	1	13
0,46	0,77	1	13
0,45	0,608	2	30
0,45	0,678	2	30
0,45	0,915	2	30
0,45	0,914	2	30
0,43	0,635	3	48
0,43	0,623	3	48
0,42	0,713	5	85
0,4	0,683	8	142
0,4	0,691	2	31
0,4	0,654	2	31
0,4	0,759	2	31
0,4	0,757	2	31
0,39	0,692	3	49
0,39	0,696	11	200
0,39	0,908	1	14
0,39	0,676	1	14
0,39	0,671	1	14
0,39	0,963	1	14
0,39	0,769	1	14
0,37	0,72	4	68
0,36	0,713	6	106
0,35	0,733	2	32
0,35	0,668	2	32
0,32	0,775	1	15
0,32	0,792	1	15
0,32	0,803	1	15
0,32	0,681	1	15
0,32	0,803	1	15
0,32	0,793	1	15

0,32	0,998	1	15
0,32	0,875	1	15
0,32	0,937	1	15
0,32	0,98	1	15
0,31	0,738	5	89
0,31	0,961	2	33
0,29	0,707	3	52
0,28	0,817	8	148
0,27	0,802	11	207
0,26	0,773	2	34
0,26	0,809	2	34
0,26	0,681	2	34
0,26	0,783	2	34
0,26	0,799	5	91
0,25	0,867	4	72
0,25	0,672	3	53
0,25	0,882	1	16
0,25	0,893	1	16
0,25	0,689	1	16
0,25	0,909	1	16
0,25	0,997	1	16
0,25	0,992	1	16
0,22	0,813	8	151
0,22	0,703	3	54
0,19	0,86	3	55
0,19	0,859	1	17
0,19	0,923	1	17
0,19	0,728	1	17
0,19	0,999	1	17
0,19	0,999	1	17
0,19	0,999	1	17
0,18	0,859	2	36
0,18	0,861	2	36
0,18	0,852	2	36
0,18	0,839	2	36
0,18	0,939	2	36
0,18	0,961	2	36
0,16	0,873	3	56
0,14	0,829	2	37
0,14	0,88	2	37
0,14	0,905	2	37
0,14	0,903	2	37
0,13	0,995	1	18
0,13	0,976	1	18
0,13	0,823	1	18
0,13	0,999	1	18
0,13	0,993	1	18
0,12	0,872	3	57
0,1	0,909	2	38
0,1	0,928	2	38

0,1	0,88	2	38
0,1	0,849	2	38
0,09	0,938	3	58
0,09	0,912	9	178
0,09	0,956	6	118
0,07	0,946	1	19
0,07	0,967	1	19
0,07	0,999	1	19
0,07	0,927	5	99
0,04	0,954	8	161
0,03	0,986	3	60
0,03	0,974	2	40
0,03	0,949	2	40
0,02	0,983	5	101
0,02	0,999	1	20
0,02	0,999	1	20
0,01	0,988	13	264
0,01	0,988	6	122
0	0,996	5	102
-0,01	0,999	2	41
-0,01	0,977	2	41
-0,03	0,99	3	62
-0,03	0,98	7	144
-0,03	0,983	11	226
-0,03	0,999	1	21
-0,03	0,981	1	21
-0,03	0,931	1	21
-0,03	0,95	1	21
-0,03	0,958	1	21
-0,03	0,996	1	21
-0,04	0,953	2	42
-0,04	0,99	2	42
-0,04	0,954	2	42
-0,04	0,971	2	42
-0,04	0,949	7	145
-0,05	0,974	5	104
-0,05	0,969	3	63
-0,08	0,945	2	43
-0,08	0,933	2	43
-0,08	0,91	2	43
-0,08	0,929	2	43
-0,08	0,982	1	22
-0,08	0,923	1	22
-0,08	0,998	1	22
-0,08	0,938	3	64
-0,11	0,956	3	65
-0,11	0,956	3	65
-0,11	0,949	2	44
-0,11	0,902	2	44
-0,11	0,875	2	44

-0,11	0,951	2	44
-0,12	0,881	1	23
-0,12	0,917	1	23
-0,12	0,869	1	23
-0,12	0,999	1	23
-0,12	0,999	1	23
-0,12	0,824	1	23
-0,12	0,999	1	23
-0,13	0,894	4	87
-0,13	0,937	4	87
-0,14	0,878	3	66
-0,14	0,844	3	66
-0,14	0,827	3	66
-0,14	0,896	3	66
-0,16	0,828	4	88
-0,17	0,763	1	24
-0,17	0,761	1	24
-0,17	0,759	1	24
-0,17	0,84	1	24
-0,17	0,939	1	24
-0,17	0,999	1	24
-0,18	0,833	2	46
-0,18	0,849	2	46
-0,21	0,884	1	25
-0,21	0,703	1	25
-0,21	0,921	1	25
-0,22	0,823	3	69
-0,22	0,837	8	176
-0,23	0,829	4	91
-0,23	0,831	0	1
-0,23	0,967	0	1
-0,23	0,699	0	1
-0,23	0,992	0	1
-0,23	0,595	0	1
-0,23	0,594	0	1
-0,23	0,861	0	1
-0,23	0,962	0	1
-0,23	0,938	0	1
-0,23	0,928	0	1
-0,23	0,318	0	1
-0,23	0,998	0	1
-0,24	0,899	2	48
-0,24	0,797	2	48
-0,24	0,802	5	113
-0,25	0,952	1	26
-0,25	0,684	1	26
-0,25	0,874	1	26
-0,25	0,928	1	26
-0,25	0,989	1	26

-0,27	0,796	3	71
-0,27	0,839	3	71
-0,27	0,807	2	49
-0,27	0,812	6	136
-0,27	0,804	6	136
-0,28	0,782	5	115
-0,29	0,766	1	27
-0,29	0,664	1	27
-0,29	0,995	1	27
-0,29	0,85	3	72
-0,29	0,76	3	72
-0,3	0,685	2	50
-0,3	0,835	2	50
-0,32	0,813	3	73
-0,32	0,728	5	117
-0,32	0,993	0	2
-0,32	0,498	0	2
-0,32	0,757	0	2
-0,32	0,99	0	2
-0,32	0,405	0	2
-0,32	0,819	0	2
-0,32	0,869	0	2
-0,32	0,433	0	2
-0,32	0,97	0	2
-0,32	0,39	0	2
-0,33	0,733	2	51
-0,33	0,659	1	28
-0,33	0,97	1	28
-0,33	0,754	7	161
-0,34	0,746	3	74
-0,35	0,673	2	52
-0,36	0,715	1	29
-0,36	0,652	1	29
-0,36	0,974	1	29
-0,36	0,668	1	29
-0,36	0,934	1	29
-0,38	0,719	9	207
-0,39	0,753	3	76
-0,39	0,901	0	3
-0,39	0,975	0	3
-0,39	0,695	0	3
-0,39	0,974	0	3
-0,39	0,974	0	3
-0,39	0,771	0	3
-0,39	0,906	0	3
-0,39	0,889	0	3
-0,39	0,749	0	3
-0,39	0,927	0	3
-0,39	0,722	0	3
-0,39	0,363	0	3

-0,39	0,785	0	3
-0,39	0,193	0	3
-0,39	0,704	0	3
-0,4	0,646	1	30
-0,4	0,701	1	30
-0,4	0,885	1	30
-0,4	0,631	1	30
-0,4	0,625	1	30
-0,4	0,667	1	30
-0,4	0,944	1	30
-0,4	0,824	1	30
-0,4	0,886	1	30
-0,4	0,668	8	187
-0,41	0,629	2	54
-0,43	0,622	1	31
-0,43	0,616	1	31
-0,43	0,676	1	31
-0,43	0,635	1	31
-0,43	0,897	1	31
-0,43	0,88	1	31
-0,43	0,686	1	31
-0,44	0,669	2	55
-0,44	0,656	2	55
-0,45	0,641	5	124
-0,45	0,959	0	4
-0,45	0,948	0	4
-0,45	0,924	0	4
-0,45	0,858	0	4
-0,45	0,841	0	4
-0,45	0,855	0	4
-0,45	0,356	0	4
-0,45	0,801	0	4
-0,45	0,729	0	4
-0,45	0,923	0	4
-0,45	0,341	0	4
-0,45	0,125	0	4
-0,45	0,965	0	4
-0,45	0,333	0	4
-0,45	0,56	0	4
-0,46	0,693	4	102
-0,47	0,598	1	32
-0,47	0,631	1	32
-0,47	0,698	1	32
-0,5	0,86	1	33
-0,5	0,698	1	33
-0,5	0,613	1	33
-0,51	0,615	9	216
-0,51	0,754	0	5
-0,51	0,624	0	5
-0,51	0,537	0	5

-0,51	0,468	0	5
-0,51	0,624	0	5
-0,51	0,888	0	5
-0,51	0,635	0	5
-0,51	0,433	0	5
-0,51	0,897	0	5
-0,51	0,551	0	5
-0,51	0,275	0	5
-0,51	0,151	0	5
-0,51	0,292	0	5
-0,51	0,395	0	5
-0,51	0,201	0	5
-0,51	0,114	0	5
-0,51	0,906	0	5
-0,51	0,157	0	5
-0,51	0,717	0	5
-0,51	0,483	0	5
-0,51	0,634	7	172
-0,52	0,604	2	58
-0,52	0,569	3	82
-0,52	0,602	3	82
-0,53	0,627	7	173
-0,53	0,652	1	34
-0,53	0,616	1	34
-0,53	0,584	1	34
-0,53	0,63	1	34
-0,54	0,641	2	59
-0,55	0,54	3	83
-0,55	0,594	12	285
-0,56	0,775	0	6
-0,56	0,707	0	6
-0,56	0,714	0	6
-0,56	0,666	0	6
-0,56	0,803	0	6
-0,56	0,757	0	6
-0,56	0,245	0	6
-0,56	0,844	0	6
-0,56	0,124	0	6
-0,56	0,786	0	6
-0,56	0,128	0	6
-0,56	0,529	0	6
-0,56	0,128	0	6
-0,56	0,113	0	6
-0,56	0,252	0	6
-0,56	0,794	0	6
-0,56	0,151	0	6
-0,56	0,59	1	35
-0,56	0,699	1	35
-0,57	0,619	2	60
-0,57	0,596	8	198

-0,58	0,564	5	131
-0,58	0,639	4	108
-0,59	0,545	3	85
-0,59	0,556	2	61
-0,59	0,544	2	61
-0,59	0,507	1	36
-0,59	0,594	1	36
-0,6	0,538	5	132
-0,6	0,54	6	155
-0,6	0,497	0	7
-0,6	0,613	0	7
-0,6	0,498	0	7
-0,6	0,323	0	7
-0,6	0,572	0	7
-0,6	0,338	0	7
-0,6	0,136	0	7
-0,6	0,445	0	7
-0,6	0,175	0	7
-0,6	0,141	0	7
-0,6	0,145	0	7
-0,6	0,122	0	7
-0,6	0,47	0	7
-0,6	0,408	0	7
-0,61	0,561	3	86
-0,62	0,547	4	110
-0,62	0,49	9	224
-0,62	0,656	1	37
-0,63	0,481	3	87
-0,63	0,511	3	87
-0,63	0,532	5	134
-0,64	0,556	2	63
-0,64	0,662	0	8
-0,64	0,689	0	8
-0,64	0,39	0	8
-0,64	0,624	0	8
-0,64	0,592	0	8
-0,64	0,671	0	8
-0,64	0,649	0	8
-0,64	0,554	0	8
-0,64	0,617	0	8
-0,64	0,206	0	8
-0,64	0,3	0	8
-0,64	0,592	0	8
-0,64	0,679	0	8
-0,64	0,183	0	8
-0,64	0,302	0	8
-0,64	0,226	0	8
-0,64	0,163	0	8
-0,64	0,578	0	8
-0,65	0,541	1	38

-0,65	0,469	1	38
-0,65	0,487	1	38
-0,65	0,548	3	88
-0,66	0,52	2	64
-0,67	0,549	3	89
-0,68	0,513	1	39
-0,68	0,461	1	39
-0,68	0,483	6	160
-0,68	0,639	0	9
-0,68	0,651	0	9
-0,68	0,616	0	9
-0,68	0,485	0	9
-0,68	0,59	0	9
-0,68	0,621	0	9
-0,68	0,522	0	9
-0,68	0,619	0	9
-0,68	0,617	0	9
-0,68	0,651	0	9
-0,68	0,521	0	9
-0,68	0,39	0	9
-0,68	0,17	0	9
-0,68	0,15	0	9
-0,68	0,633	0	9
-0,68	0,604	0	9
-0,68	0,176	0	9
-0,68	0,558	0	9
-0,69	0,53	3	90
-0,69	0,524	3	90
-0,7	0,516	5	138
-0,71	0,526	1	40
-0,71	0,523	1	40
-0,71	0,464	1	40
-0,72	0,473	9	231
-0,72	0,509	0	10
-0,72	0,443	0	10
-0,72	0,625	0	10
-0,72	0,543	0	10
-0,72	0,462	0	10
-0,72	0,516	0	10
-0,72	0,645	0	10
-0,72	0,568	0	10
-0,72	0,286	0	10
-0,72	0,297	0	10
-0,72	0,423	0	10
-0,72	0,2	0	10
-0,72	0,234	0	10
-0,73	0,436	2	67
-0,73	0,447	2	67
-0,73	0,621	1	41
-0,75	0,507	5	141

-0,75	0,436	0	11
-0,75	0,204	0	11
-0,75	0,272	0	11
-0,75	0,325	0	11
-0,75	0,614	0	11
-0,75	0,338	0	11
-0,75	0,232	0	11
-0,75	0,284	0	11
-0,75	0,212	0	11
-0,75	0,228	0	11
-0,75	0,18	0	11
-0,75	0,24	0	11
-0,76	0,397	1	42
-0,76	0,604	1	42
-0,77	0,437	4	118
-0,77	0,455	2	69
-0,77	0,447	2	69
-0,78	0,397	1	43
-0,78	0,428	1	43
-0,79	0,234	0	12
-0,79	0,465	0	12
-0,79	0,603	0	12
-0,79	0,594	0	12
-0,79	0,516	0	12
-0,79	0,278	0	12
-0,79	0,28	0	12
-0,79	0,223	0	12
-0,79	0,571	0	12
-0,79	0,257	0	12
-0,79	0,3	0	12
-0,79	0,205	0	12
-0,79	0,216	0	12
-0,8	0,423	2	70
-0,81	0,481	1	44
-0,82	0,425	2	71
-0,82	0,565	0	13
-0,82	0,289	0	13
-0,82	0,256	0	13
-0,82	0,569	0	13
-0,82	0,244	0	13
-0,82	0,254	0	13
-0,82	0,336	0	13
-0,82	0,541	0	13
-0,82	0,347	0	13
-0,82	0,232	0	13
-0,82	0,33	0	13
-0,83	0,552	1	45
-0,85	0,452	0	14
-0,85	0,244	0	14
-0,85	0,44	0	14

-0,85	0,454	0	14
-0,85	0,251	0	14
-0,85	0,241	0	14
-0,85	0,272	0	14
-0,85	0,409	0	14
-0,86	0,366	2	73
-0,86	0,545	1	46
-0,86	0,439	1	46
-0,88	0,376	2	74
-0,88	0,422	0	15
-0,88	0,207	0	15
-0,88	0,205	0	15
-0,88	0,465	0	15
-0,88	0,424	0	15
-0,88	0,505	0	15
-0,88	0,472	0	15
-0,88	0,207	0	15
-0,88	0,166	0	15
-0,88	0,472	1	47
-0,88	0,359	1	47
-0,89	0,351	3	100
-0,91	0,375	1	48
-0,91	0,394	1	48
-0,91	0,352	1	48
-0,91	0,481	1	48
-0,91	0,458	0	16
-0,91	0,473	0	16
-0,91	0,256	0	16
-0,91	0,352	0	16
-0,91	0,218	0	16
-0,91	0,213	0	16
-0,91	0,189	0	16
-0,92	0,409	4	127
-0,92	0,427	2	76
-0,93	0,346	1	49
-0,93	0,357	1	49
-0,94	0,439	0	17
-0,94	0,16	0	17
-0,94	0,371	0	17
-0,94	0,392	0	17
-0,94	0,275	0	17
-0,94	0,206	0	17
-0,94	0,177	0	17
-0,94	0,194	0	17
-0,94	0,2	0	17
-0,94	0,341	2	77
-0,95	0,359	1	50
-0,95	0,449	1	50
-0,95	0,433	1	50
-0,95	0,322	4	129

-0,96	0,235	0	18
-0,96	0,151	0	18
-0,96	0,293	0	18
-0,96	0,375	0	18
-0,96	0,432	0	18
-0,96	0,311	0	18
-0,96	0,338	0	18
-0,96	0,178	0	18
-0,96	0,457	0	18
-0,96	0,186	0	18
-0,96	0,23	0	18
-0,96	0,33	0	18
-0,98	0,339	1	51
-0,98	0,347	1	51
-0,98	0,34	2	79
-0,98	0,326	2	79
-0,99	0,186	0	19
-0,99	0,353	0	19
-0,99	0,312	0	19
-0,99	0,193	0	19
-0,99	0,209	0	19
-0,99	0,204	0	19
-1	0,405	1	52
-1,01	0,286	3	107
-1,02	0,126	0	20
-1,02	0,217	0	20
-1,02	0,288	0	20
-1,02	0,115	0	20
-1,02	0,13	0	20
-1,02	0,343	0	20
-1,02	0,144	0	20
-1,02	0,186	0	20
-1,02	0,279	1	53
-1,03	0,302	3	108
-1,04	0,365	2	82
-1,04	0,303	1	54
-1,04	0,314	1	54
-1,04	0,359	0	21
-1,04	0,121	0	21
-1,04	0,132	0	21
-1,04	0,249	3	109
-1,06	0,286	3	110
-1,06	0,304	7	210
-1,07	0,13	0	22
-1,07	0,155	0	22
-1,07	0,269	0	22
-1,07	0,146	0	22
-1,07	0,132	0	22
-1,07	0,292	2	84

-1,08	0,295	3	111
-1,08	0,282	1	56
-1,08	0,269	1	56
-1,08	0,321	1	56
-1,09	0,139	0	23
-1,09	0,389	0	23
-1,09	0,24	2	85
-1,09	0,264	2	85
-1,1	0,263	4	138
-1,1	0,287	1	57
-1,11	0,342	2	86
-1,11	0,306	2	86
-1,11	0,325	2	86
-1,11	0,359	2	86
-1,11	0,235	3	113
-1,11	0,285	4	139
-1,11	0,114	0	24
-1,11	0,203	0	24
-1,12	0,177	1	58
-1,13	0,248	3	114
-1,13	0,33	2	87
-1,14	0,122	0	25
-1,14	0,194	0	25
-1,14	0,115	0	25
-1,14	0,134	0	25
-1,14	0,116	0	25
-1,14	0,304	1	59
-1,14	0,168	1	59
-1,14	0,234	3	115
-1,16	0,117	0	26
-1,16	0,297	0	26
-1,16	0,213	0	26
-1,16	0,123	0	26
-1,16	0,28	3	116
-1,16	0,214	1	60
-1,16	0,256	2	89
-1,16	0,26	2	89
-1,18	0,232	7	219
-1,18	0,138	0	27
-1,18	0,101	0	27
-1,18	0,127	0	27
-1,18	0,137	0	27
-1,18	0,217	0	27
-1,18	0,109	0	27
-1,18	0,298	2	90
-1,2	0,195	2	91
-1,2	0,157	1	62
-1,2	0,198	1	62
-1,2	0,115	0	28
-1,2	0,272	0	28

-1,2	0,139	0	28
-1,2	0,26	0	28
-1,2	0,166	0	28
-1,2	0,121	0	28
-1,2	0,148	0	28
-1,2	0,151	0	28
-1,2	0,14	0	28
-1,2	0,111	0	28
-1,2	0,149	0	28
-1,22	0,253	2	92
-1,22	0,244	4	146
-1,22	0,283	1	63
-1,22	0,136	0	29
-1,22	0,107	0	29
-1,22	0,126	0	29
-1,22	0,117	0	29
-1,22	0,098	0	29
-1,22	0,144	0	29
-1,22	0,13	0	29
-1,22	0,099	0	29
-1,22	0,124	0	29
-1,24	0,202	1	64
-1,25	0,158	0	30
-1,25	0,269	0	30
-1,25	0,115	0	30
-1,25	0,14	0	30
-1,26	0,238	1	65
-1,27	0,169	0	31
-1,27	0,245	0	31
-1,27	0,12	0	31
-1,27	0,218	0	31
-1,27	0,126	0	31
-1,27	0,228	3	123
-1,29	0,118	0	32
-1,3	0,23	1	67
-1,3	0,191	2	97
-1,31	0,102	0	33
-1,31	0,11	0	33
-1,31	0,111	0	33
-1,31	0,132	0	33
-1,33	0,1	0	34
-1,33	0,132	0	34
-1,33	0,116	0	34
-1,33	0,096	1	69
-1,35	0,117	0	35
-1,35	0,223	0	35
-1,35	0,173	3	128
-1,35	0,179	1	70
-1,36	0,111	0	36
-1,36	0,196	0	36

-1,36	0,18	0	36
-1,38	0,158	0	37
-1,38	0,101	0	37
-1,38	0,107	0	37
-1,38	0,1	0	37
-1,38	0,114	0	37
-1,4	0,139	2	103
-1,4	0,102	0	38
-1,4	0,102	0	38
-1,4	0,153	4	159
-1,42	0,092	0	39
-1,42	0,194	0	39
-1,42	0,086	0	39
-1,42	0,152	3	133
-1,43	0,146	4	161
-1,44	0,175	3	134
-1,44	0,12	0	40
-1,44	0,084	0	40
-1,44	0,102	0	40
-1,45	0,148	3	135
-1,45	0,09	1	76
-1,46	0,103	0	41
-1,46	0,13	0	41
-1,46	0,166	0	41
-1,46	0,103	0	41
-1,46	0,088	0	41
-1,47	0,075	1	77
-1,47	0,111	1	77
-1,47	0,119	1	77
-1,47	0,125	0	42
-1,47	0,163	0	42
-1,47	0,087	0	42
-1,47	0,093	0	42
-1,49	0,085	0	43
-1,49	0,094	0	43
-1,5	0,114	1	79
-1,5	0,135	1	79
-1,51	0,082	0	44
-1,51	0,102	0	44
-1,51	0,078	0	44
-1,52	0,087	1	80
-1,52	0,126	1	80
-1,52	0,127	2	111
-1,53	0,122	3	141
-1,54	0,071	0	46
-1,54	0,072	0	46
-1,54	0,117	0	46
-1,54	0,091	0	46
-1,56	0,118	1	83
-1,56	0,109	1	83

-1,58	0,12	0	48
-1,58	0,063	0	48
-1,58	0,077	0	48
-1,59	0,102	2	116
-1,6	0,109	3	146
-1,61	0,073	0	50
-1,61	0,109	1	86
-1,61	0,117	1	86
-1,62	0,096	0	51
-1,63	0,087	4	176
-1,63	0,092	4	176
-1,63	0,118	3	148
-1,63	0,082	2	119
-1,64	0,076	0	52
-1,64	0,079	0	52
-1,65	0,097	2	120
-1,66	0,113	0	53
-1,66	0,088	1	89
-1,66	0,084	1	89
-1,67	0,1	5	207
-1,67	0,085	1	90
-1,67	0,067	1	90
-1,67	0,076	0	54
-1,68	0,101	3	152
-1,69	0,071	1	91
-1,69	0,064	0	55
-1,69	0,099	0	55
-1,69	0,078	0	55
-1,69	0,064	0	55
-1,69	0,081	4	181
-1,7	0,067	1	92
-1,7	0,081	1	92
-1,73	0,073	0	58
-1,73	0,051	0	58
-1,73	0,067	0	58
-1,74	0,06	1	95
-1,75	0,055	0	59
-1,75	0,075	0	59
-1,76	0,084	2	128
-1,76	0,066	6	242
-1,77	0,074	10	348
-1,78	0,06	0	61
-1,78	0,077	4	189
-1,79	0,051	0	62
-1,8	0,079	3	161
-1,81	0,067	0	63
-1,82	0,045	0	64
-1,83	0,047	0	65
-1,84	0,054	1	102
-1,86	0,051	0	67

-1,86	0,046	0	67
-1,87	0,053	1	104
-1,87	0,043	3	167
-1,87	0,056	2	137
-1,9	0,037	0	70
-1,9	0,04	0	70
-1,91	0,044	3	170
-1,92	0,045	0	71
-1,93	0,045	0	72
-1,93	0,039	0	72
-1,97	0,042	0	75
-1,97	0,035	0	75
-1,98	0,047	0	76
-2,01	0,05	0	78
-2,01	0,038	1	115
-2,01	0,045	1	115
-2,02	0,039	1	116
-2,05	0,036	0	81
-2,07	0,037	1	120
-2,07	0,031	1	120
-2,07	0,029	0	83
-2,07	0,041	0	83
-2,08	0,037	2	154
-2,08	0,031	1	121
-2,13	0,025	2	158
-2,15	0,036	0	89
-2,16	0,034	1	127
-2,16	0,024	0	90
-2,17	0,036	1	128
-2,18	0,024	0	92
-2,19	0,025	4	225
-2,2	0,042	0	93
-2,2	0,025	0	93
-2,2	0,018	1	131
-2,23	0,028	2	167
-2,24	0,026	0	97
-2,27	0,018	0	99
-2,28	0,027	0	100
-2,31	0,013	0	103
-2,36	0,019	0	107
-2,37	0,021	0	108
-2,37	0,015	4	243
-2,54	0,018	0	124
-2,57	0,006	0	127
-2,62	0,009	0	132
-2,67	0,006	0	137
-2,93	0,007	1	203
-3,08	0,003	2	256
-3,12	0,002	1	225
-3,21	0,003	4	340

NaN	0	0	0
NaN	0	0	0
NaN	0	0	0
NaN	0	0	0
NaN	0	0	0
NaN	0	0	0
NaN	0	0	0
NaN	0	0	0
